# Total Syntheses of (±)-Lepadiformine B and C

**DOI:** 10.1021/acs.joc.5c00738

**Published:** 2025-07-04

**Authors:** Wei-Ting Hsiao, Jui-Lin Wu, Wen-Hua Chiou

**Affiliations:** Department of Chemistry, 34916 National Chung-Hsing University, Taichung 402 ,Taiwan, ROC

## Abstract

Total syntheses of (±)-lepadiformine B and C are presented. The key theme in our approach is the stereodivergent synthesis of both *cis*- and *trans*- *N*-acetyl-2-alkyl-8a-cyanodecahydroquinoline, which are effectively prepared through deprotection-initiated alkylative/reductive cyclization of sterically well-defined α-aminonitriles bearing a masked carbonyl group. After the Dieckmann-type condensation to a tricyclic lactam, lepadiformine B and its analogues can be achieved through the Ir-catalyzed reductive cyanation and subsequent hydrolysis, reduction, and Bruylants reactions.

## Graphical abstract

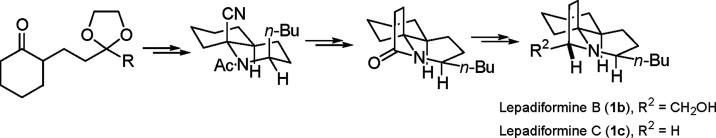

In 2006, Sauviat et al. reported the isolation of marine alkaloids lepadiformine B (**1b**) and lepadiformine C (**1c**) (Figure 1) from the tunicate *Clavelina moluccensis* Sluiter collected in Djibouti waters.^1^ Lepadiformine B (**1b**) and C (**1c**) are tricyclic alkaloids that possess a tricyclic perhydropyrido­[2,1-*j*]-quinolone structure bearing an *n*-butyl group at the C2 position, and their difference is the presence of the hydroxymethyl group at the C13 position in **1b**. Lepadiformine B (**1b**) shows moderate inhibition of the cardiac inward rectifier K^+^ channel compared to its analogue lepadiformine A (**1a**) bearing an *n*-hexyl group, while **1c** shows poor inhibition activity. These findings suggest that the length of the C2 side chain and the presence of the C13 hydroxymethyl group do matter. In addition, these lepadiformine alkaloids also exhibit moderate cytotoxicity against several tumor cell lines.^2^ As the first reported member in the lepadiformine family, lepadiformine A (**1a**) was isolated by Biard in 1994, from the marine ascidian Müller in Tunisia.^3^ The correct structure was confirmed through total synthesis by Kibayashi and co-workers in 2000.^4^ The interesting structure and biological activities have prompted chemists to develop various intellectual and elegant strategies, simply classified as racemate syntheses,^5^ enantiomeric syntheses,^4,6^ and reviews.^7^

**Figure 1:**
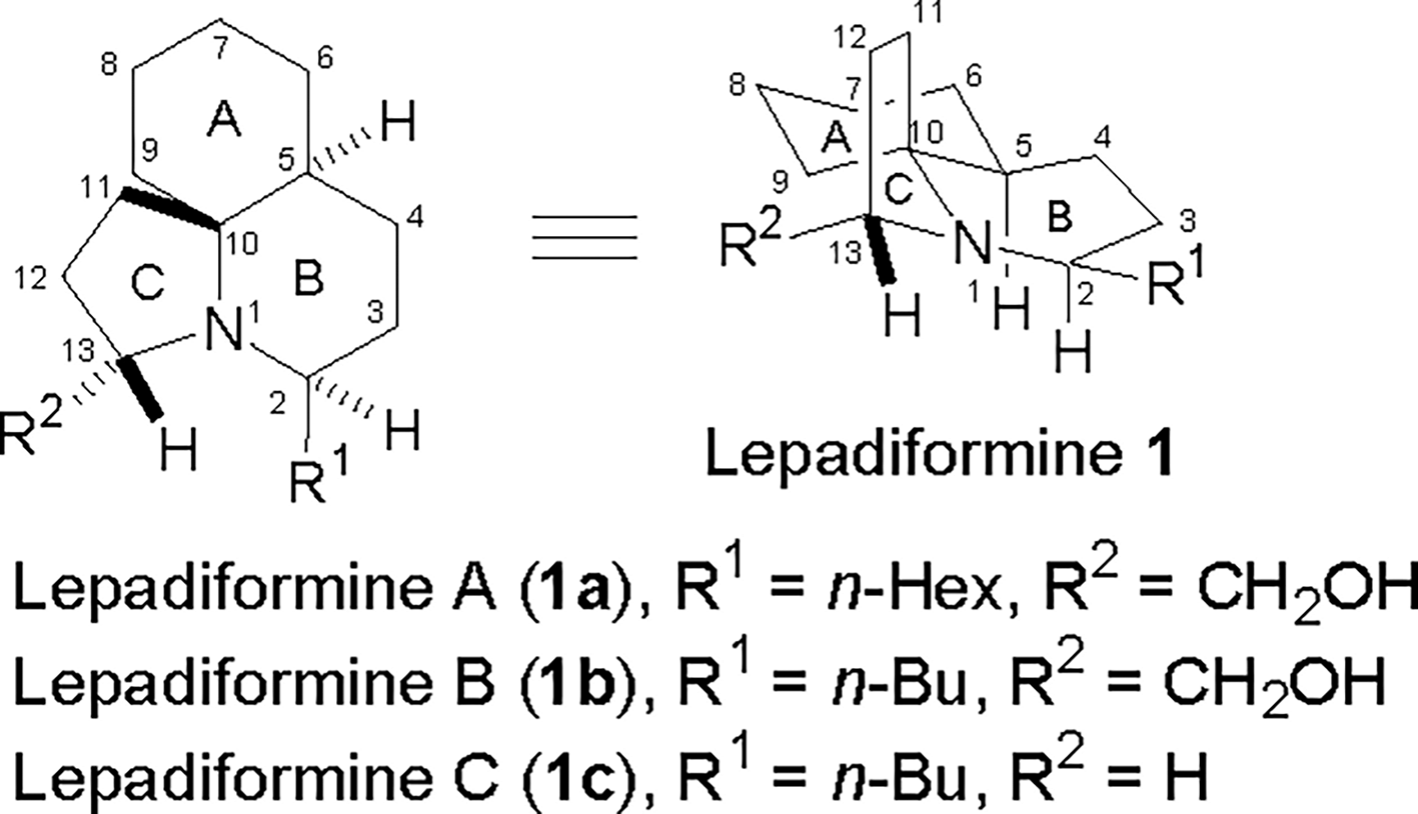
Structures of the lepadiformine family.

Recently, we developed a divergent approach to both *cis*- and *trans*-*N*-acetyl-2-alkyl-8a-cyano-decahydroquinoline (8a-CDHQ) structures via a double consecutive epimerization from the same intermediate Cbz-protected *cis*-2-allyl-8a-CDHQ. Subsequent base-mediated Dieckmann-type condensation of the *N*-acetyl-2-alkyl-8a-CDHQs furnished a tricyclic lactam with either *cis* or *trans* configuration in the B ring.^8^ Through this strategy, we completed the synthesis of fasicularin, a tunicate alkaloid.^9^ In this article, we describe the extension of this work, intended for use in the total syntheses of (±)-lepadiformine B (**1b**) and C (**1c**).

Our retrosynthetic analysis is shown in Scheme 1. The hydroxymethyl group at C13 can be introduced by a one-carbon elongation of tertiary lactam **2** through Dixon’s reductive cyanation^10^ and subsequent transformations. A Dieckmann-type condensation between the angular nitrile with the acetyl group in *cis*-acetamide **3** can generate the pyrrolidine C ring of lactam **2**. Different from the approach to fasicularin, in which the necessary *trans* configuration in the B ring is achieved through epimerization, conversion to *cis*-acetamide **3** from the known Cbz-protected *cis*-2-allyl-8a-cyanodecahydroquinoline (**4**) involves relative configuration retention. Although *cis*-8a-CDHQ **4** can be available based on our previously established method, an improved procedure has been developed for more efficient preparation. If the α-aminonitrile derivative **5a** bearing a propyl substituent with a masked aldehyde group, i.e., a terminal ethylene acetal, is reacted with Lewis acid and allyltrimethylsilane, spontaneous cyclization to 8a-CDHQ **4** may proceed in a domino manner: simultaneous cleavage of the dioxolane moiety and formation of a transient piperidinium, followed by addition with allyltrimethylsilane. Similarly, as an α-aminonitrile bearing an ethylene ketal group with the correct position and side chain length is reacted within Lewis acid and triethylsilane, it will result in the formation of 8a-CDHQ with the opposite configuration at the C2 position, as long as both additions follow the identical mechanism. Such a deprotection-initiated alkylative/reductive cyclization approach may save manipulations for the construction of the B ring and the side chain and provide divergent syntheses for both *cis*- and *trans*-8a-CDHQ. The sterically well-defined α-aminonitrile **5** can be obtained by a Strecker reaction of the α-monosubstituted cyclohexanone derivative **6**.

**Scheme 1:**
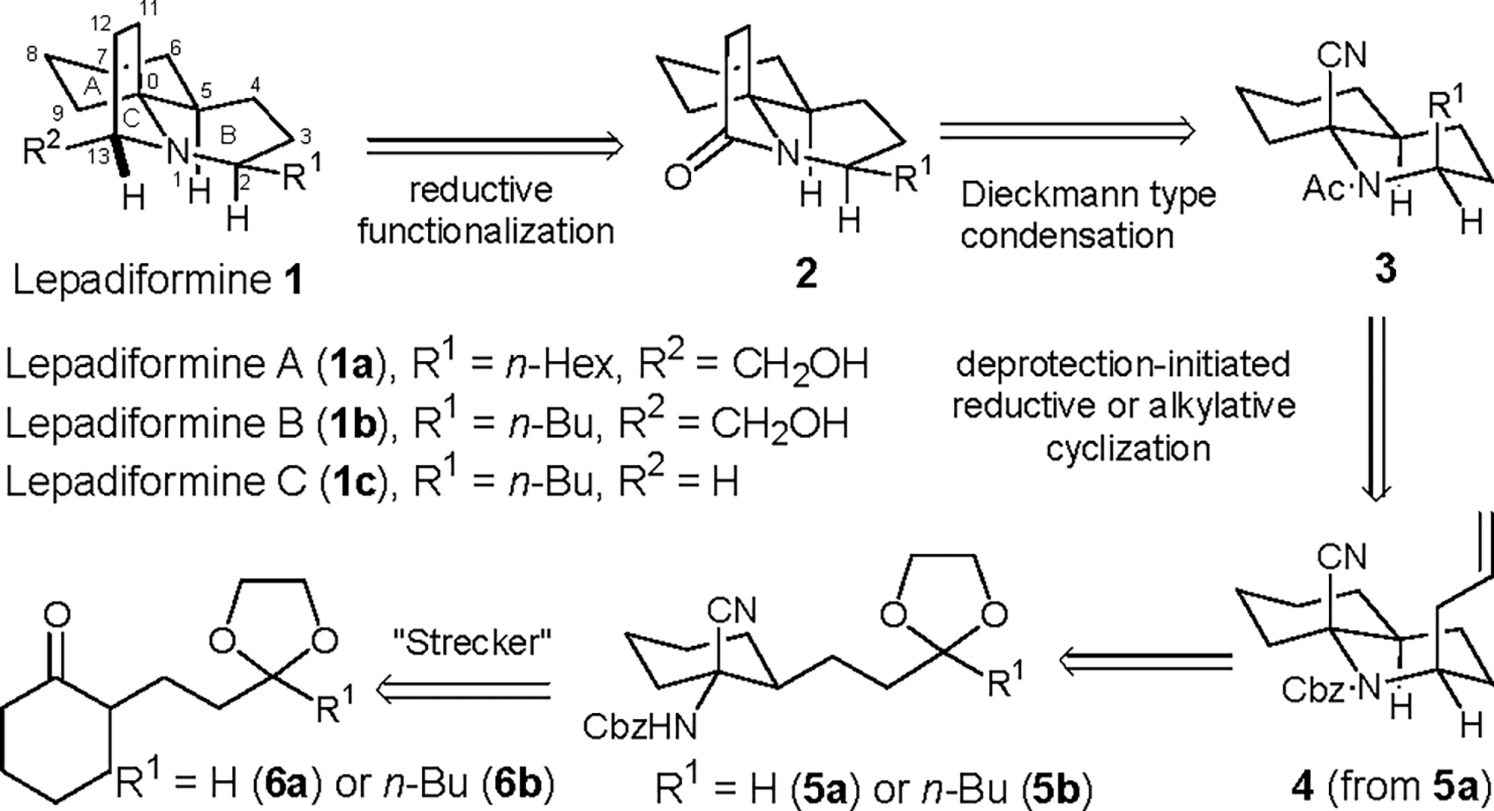
Retrosynthetic Analysis of Lepadiformine **1**

Our synthesis commences with the preparation of **6** through the α-alkylation of cyclohexanone. To find the optimal α-alkylation conditions, commercially available 1-bromo-3,3-ethylenedioxypropane (**7a**) was chosen for the optimization of the conditions of alkylation based on Kita’s protocol^11^ (see Supporting Information). Treatment of bromide **7a** with 3 equiv of cyclohexanone and LiHMDS in DMF at −50 °C proceeded smoothly to give 81% yield of the desired product **6a** (Scheme 2). The analogue **6b** was also achieved in 72% isolated yield with bromide **7b**. The subsequent Strecker reaction of **6a**, i.e., with KCN in ammonia at room temperature, and CbzCl protection proceeded readily to deliver single α-aminonitrile **5a** in 91% yield. This tactic was employed to obtain **5b** in 84% yield.

**Scheme 2:**
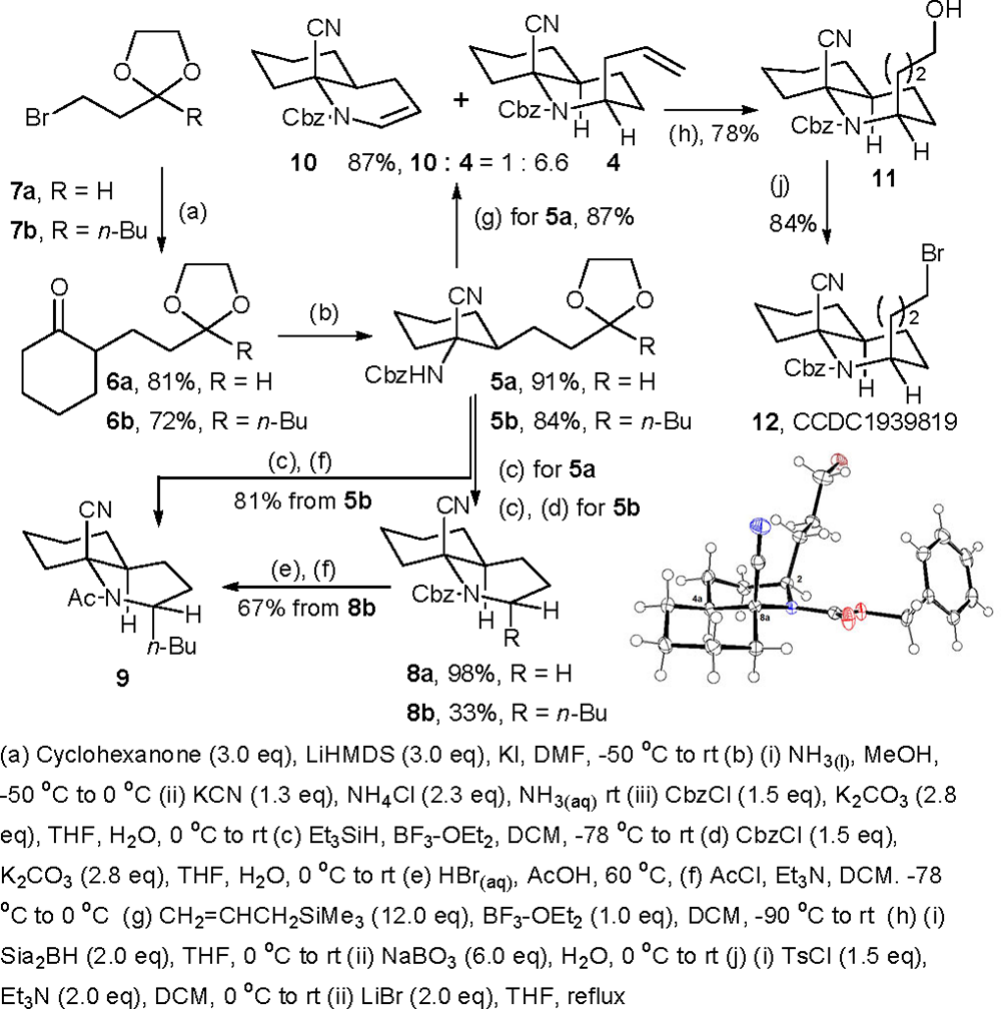
Improved Synthesis toward *cis-* and *trans*-8a-CDHQ **4** and **9**

The conditions for the deprotectively reductive cyclization were investigated (see Supporting Information for more details); we found that the treatment of α-aminonitrile **5a** with 1.0 equiv of BF_3_·OEt_2_ and triethylsilane afforded the cyclized product **8a** in excellent yield. The application of the conditions on aminonitrile **5b** did not yield the expected cyclized product, but a Cbz-free uncyclized product, which was then subjected to protection with CbzCl back to the original **5b**. Treatment with 1.3 equiv of BF_3_·OEt_2_ and triethylsilane resulted the formation of a Cbz-free cyclized product, which was protected with CbzCl to produce the cyclized product **8b** in 33% yield. The results showed substantial reactivity difference between acetal **5a** and ketal **5b**. For acetal **5a**, cleavage of the dioxolane group proceeds first, followed by reductive cyclization, while the removal of the Cbz group proceeds first for ketal **5b**, followed by the cleavage of dioxolane and reductive cyclization. Such a series of reactions could be viewed as a domino process.^12^ However, to our dismay, the NMR spectra of **8b** were *not* identical to those of the known Cbz-protected *cis*-2-butyl-8a-CDHQ, and this might suggest a *trans* product. To verify the supposition, the Cbz group was transformed to an acetyl group using the protocol of HBr/AcOH deprotection and then acetylation. The spectra of the resulting product were consistent with those of known *trans*-N-acetyl-2-*n*-butyl-8a-CDHQ (**9**). Hence, the results confirm that the deprotectively reductive cyclization of **5b** affords the product with the *trans* configuration rather than the desired *cis* configuration. Even so, as a crucial intermediate for the synthesis of fasicularin, the yield of the *trans* acetyl product **9** was improved up to 81% through direct treatment of **5b** with 2 equiv BF_3_·OEt_2_ and triethylsilane followed by acetylation. This concise approach allows the rapid preparation of *trans*-**9** within three steps from **7b** through simple operations.

Since deprotectively reductive cyclization of ketal **5b** affords the *trans* product, we may infer that deprotectively allylative cyclization of acetal **5a** will result in the formation of the *cis* product. Thus, treatment of **5a** with BF_3_·OEt_2_ and allyltrimethylsilane triggered a deprotection-initiated allylative cyclization and resulted in an inseparable mixture of the desired *cis*-2-allyl-8a-CDHQ (**4**) and enecarbamate **10** (∼6.6:1) in 87% combined yield (for optimization of the deprotection-initiated allylative cyclization, see Supporting Information). Subjecting the mixture to Sia_2_BH-mediated hydroboration/oxidation could yield primary alcohol **11** in 78% yield and enecarbamate **10** in 16% yield. The spectral data of the products were consistent with our previously published values. Moreover, to our delight, simple treatment of primary alcohol **11** with TsCl followed by substitution with LiBr gave a yield of 84% of crystalline bromide **12**, whose structure was established unequivocally by X-ray analysis (CCDC No. 1939819), corroborating the present *cis* configuration in primary alcohol **11** (Scheme 2). Thus, we accomplished divergent syntheses for the rapid construction of both *cis-* and *trans*-2-alkyl 8a-CDHQs: a *cis*-8a-CDHQ, e.g., **4**, could be achieved through BF_3_·OEt_2_-mediated deprotectively *allylative* cyclization of acetal **5a**, while a *trans*-8a-CDHQ, such as **9**, could be achieved through BF_3_·OEt_2_-mediated deprotectively *reductive* cyclization of ketal **5b**.

The results could be rationalized by the Fürst–Plattner rule,^13^ in which the nucleophile proceeds in an axial orientation to avoid a boat-like transition state (Scheme 3).^14^ The conformation of the B ring in **8** should take a boat form to reduce the A^1,3^ strain of the carbamate from the *n*-butyl group, which has been observed in the X-ray structures of many analogues in our previous findings.^8,9^ The synthetic route provides a divergent route for the rapid construction of both *cis* and *trans*-2-alkyl CDHQs **4** and **9**.

**Scheme 3:**
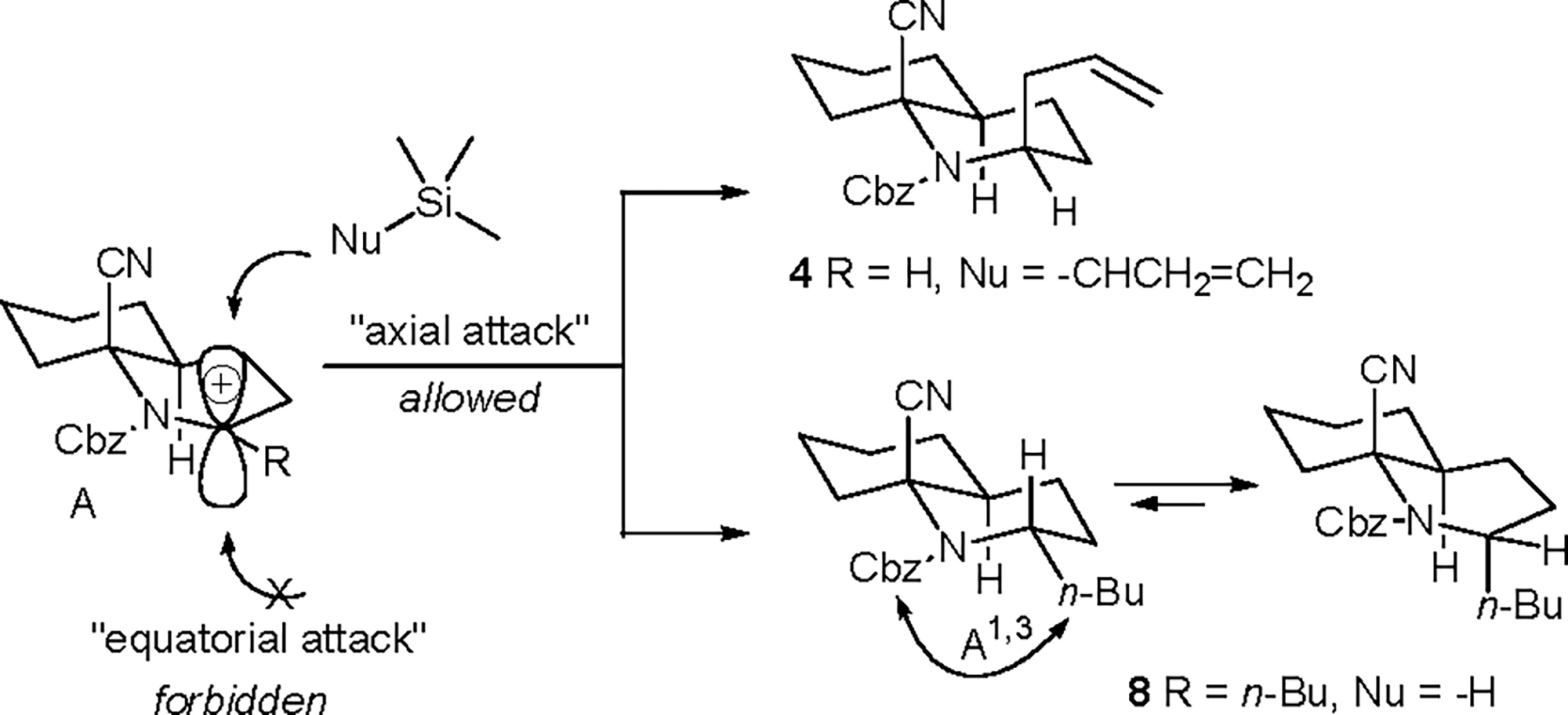
Proposed Rationale for the Formation of **4** and **8**

With alcohol **11** in hand, the *n*-butyl appendage could be achieved according to a three-step protocol (Scheme 4): the Parikh–Doering oxidation to aldehyde **13** in 66% yield, the Wittig olefination with Ph_3_P=CH_2_ to olefin **14** in 85% yield, and flavin-mediated aerobic hydrogenation conditions to **15** in 99% yield.^15^ Aerobic hydrogenation conditions only effect saturation of the alkene portion but do not remove the Cbz group, which avoids the double consecutive epimerization initiated by Pd-catalyzed hydrogenation conditions. The Cbz portion of **15** was removed within HBr in acetic acid followed by acetylation to furnish acetamide **3** in 71% overall yield. Treatment of acetamide **3** with LiHMDS followed by acidic workup afforded tricyclic ketone **16** in 77% yield. After the reduction of the ketone group of **16** with NaBH_4_, the resulting alcohol was mesylated and then eliminated by DBU to furnish conjugated lactam **17** in 87% yield over three steps. Catalytic hydrogenation at ambient pressure saturated the olefin to tricyclic lactam **2** in 90% yield. LiAlH_4_ reduction of lactam **2** afforded a 76% yield of lepadiformine C (**1c**) whose spectral properties were in accordance with the reported data.^6l^

**Scheme 4:**
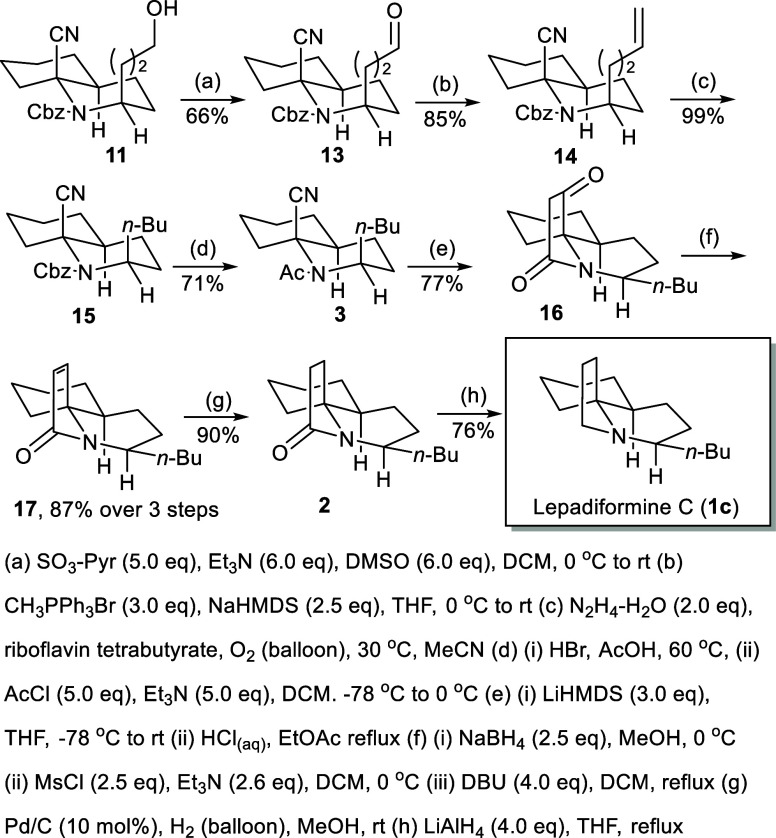
Synthesis of Lepadiformine C (**1c**)

With access to the key tricyclic lactam **2**, the introduction of a substituent on the γ-lactam could be addressed through Dixon’s Ir-catalyzed hydrosilylation–cyanation protocol (Scheme 5).^10^ Lactam **2** was able to undergo reductive cyanation to provide aminonitrile **18**, as demonstrated by our successful transformations in the synthesis of fasicularin (**I** to **II**, eq 1; Scheme 5). However, unlike tricyclic α-aminonitrile bearing a *trans*-B ring, the tricyclic *cis-*α-aminonitrile **18** appeared to be labile and easily oxidized back to lactam **2**, especially after column chromatography or a longer standing. Therefore, we envisioned that the conversion of crude tricyclic *cis-*α-aminonitrile **18** to α-substituted amines could be achieved through the Bruylants reaction.^16^ This strategy was not only used to examine the quality of the product but also provided a route to lepadiformine analogues. Thus, direct treatment of the crude aminonitrile **18** with Grignard reagents, ethynylmagnesium chloride (HC≡CMgCl), and trimethylsilylmethylmagnesium chloride (TMSCH_2_MgCl) afforded the α-substituted amines adducts **19** in 34% yield and **20** in 57% yield. Subsequent hydrolysis of α-amino nitrile **18** to the corresponding acid or carboxyl derivative turned out to be more challenging than expected. A number of hydrolysis conditions including acid, basic, or transition-metal-mediated or even under sealed or microwave-assisted forced conditions were examined (see Supporting Information), but none of these conditions could provide the desired products. Eventually, we found that Rychnovsky’s conditions of acidic methanolysis^5j^ were effective to form methyl ester **21** in 33% overall yield from tricyclic lactam **2**. Completion of the synthesis was accomplished by LiAlH_4_ reduction of methyl ester **21** to produce alcohol **1b** in 90% yield, whose spectral data were in accordance with the reported values of lepadiformine B.^6l^

**Scheme 5:**
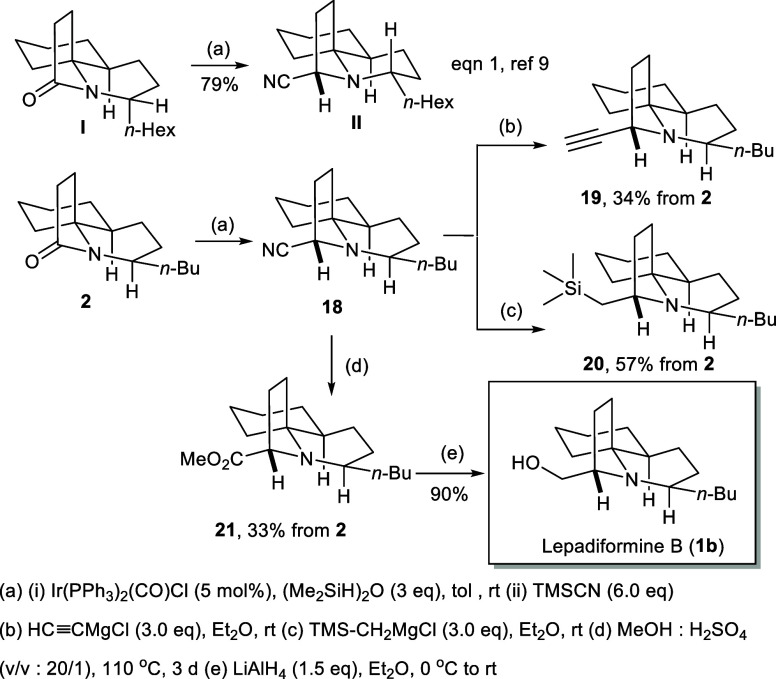
Conversion to Lepadiformine (**1b**) and Its Analogues

In conclusion, we developed an efficient protocol for the preparation of both *cis*- and *trans*-2-alkyl-8a-CDHQ, key intermediates for the syntheses of lepadiformine and fasicularin, through BF_3_·OEt_2_-initiated deprotectively silane-mediated alkylative/reductive cyclization. Cbz-protected *cis*-8a-2-allylCDHQ (**4**) can be achieved in 3 steps with 64% yield, while acetyl-protected *trans*-8a-2-*n*-butylCDHQ (**9**) can be achieved in 4 steps with 49% yield. Compared with our previous results in which **4** was obtained in 37% yield over 5 steps and **9** in 20% yield over 9 steps, this methodology facilitates the rapid and efficient assembly of two 2-alkyl-8a-CDHQ systems for the total synthesis of marine tricyclic alkaloids lepadiformine and fasicularin.

It usage was illustrated by the total syntheses of (±)-lepadiformine B and C. Lepadiformine B (**1b**) can be accomplished in 14 steps with 3.5% overall yield from cyclohexanone, while lepadiformine C (**1c**) can be accomplished in 12 steps with 9% overall yield. In addition to more efficient transformations, these reactions are readily scalable and operationally simple, not requiring harsh conditions. Various lepadiformine B analogues, such as **19** and **20**, are also achieved, as demonstrated. The work provides a new approach toward the ascidian family alkaloids, and these results will be reported in due course.

## Supplementary Material



## Data Availability

The data underlying this study are available in the published article and its Supporting Information.

## Notes

Experimental procedures, spectroscopic data of compounds, and NMR spectra of compounds; ^1^H and ^13^C NMR spectra of all compounds (PDF)

## Notes

Dedicated to Professor Iwao Ojima on occasion of his 80th birthday.
