# Supplementary material for: Total Syntheses of (±)-Lepadiformine B and C
Source: J Org Chem. 2025 Jul 4;90(28):10104–8. doi: 10.1021/acs.joc.5c00738 (PMC12281558; doi:10.1021/acs.joc.5c00738)
Supplement: Supplementary file 1 [file jo5c00738_si_001.pdf]

Supporting Information

Total Syntheses of (±)-Lepadiformine B and C

Wei-Ting Hsiao, Jui-Lin Wu and Wen-Hua Chiou*

Department of Chemistry, National Chung Hsing University,

Taichung, Taiwan, R.O.C.

e-mail: wchiou@dragon.nchu.edu.tw

Table of Contents

Investigation on preparation of 2-substituted cyclohexanone SI-1

Investigation on the Deprotectively Reductive Cyclization SI-2

Investigation on the Deprotectively Allylative Cyclization SI-3

Investigation on hydrolysis of tricyclic α-aminonitrile **18** SI-5

The $^{13}C\{^{1}H\}$ NMR comparison of lepadiformine B (**1b**), C (**1c**), **8**, **9** and **21** with literature spectra, and summaries of **1b** and **1c** SI-7~SI-12

Crystal data and structure refinement and ORTEP Drawing of compound **12** SI-13~SI-14

Experimental section SI-15~SI-33

^{1}H NMR and $^{13}C\{^{1}H\}$ NMR spectra of all compounds SI-34~SI-86

References SI-87

1. Investigation on preparation of 2-substituted cyclohexanone 6a and 6b:

Commercially available bromide **7a** was used as the substrate for investigation on alkylation on cyclohexanone. Stork enamine alkylation was carried out, but resulted in 14% yield (equation 1). Thus, we switched to direct alkylation strategy using a strong base, and the results were described as follows.

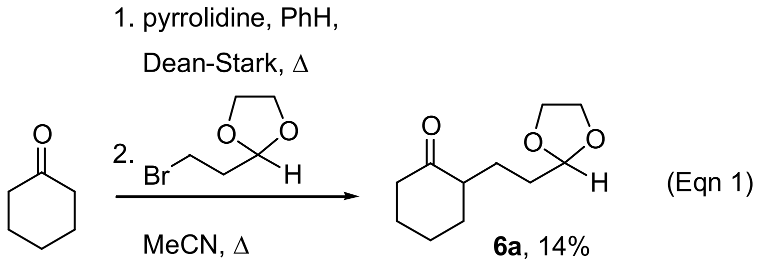

Table S1. Optimization on the alkylation of cyclohexanone with bromide **7a**:

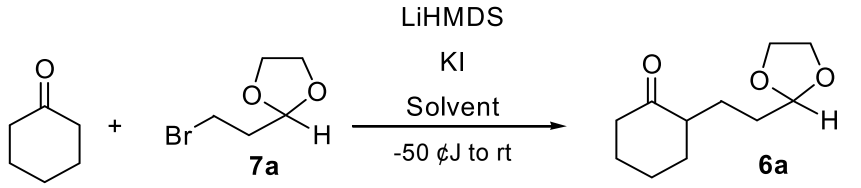

Entry[a]	Cyclohexanone (eq)	**7a** (eq)	LiHMDS (eq)	KI (eq)	Solvent (1 M)	Results[b]
1	1.0	1.1	1.1	0	THF [c]	4%
2	1.0	1.1	1.1	0	DMF	32%
3	1.0	1.1	1.1	10 mol%	DMF	35%
4	1.5	1.0	1.5	10 mol%	DMF	45%
5	2.0	1.0	2.0	10 mol%	DMF	61%
6	2.5	1.0	2.5	10 mol%	DMF	69%
7[d]	3.0	1.0	3.0	10 mol%	DMF	81%
8	3.0	1.0	3.0	10 mol%	DMF/THF (2:1)	59%

a: All reactions were conducted on 10 mmol scale. b: Isolated yield. c: -78 °C to rt. d: 30 mmol scale.

Treatment of 1.0 equivalent of cyclohexanone with 1.1 equivalent of LiHMDS and 1.1 equivalent of bromide in THF only gave poor yield (Entry 1). Switch of the solvent to DMF according to the Kita's procedure, [1] the yield was improved to 32% yield (Entry 2). Addition of 10 mol% of KI just improved the yield a little bit (35%, Entry 3). When the bromide was set as the reactant, reacting with 1.5 equivalent of cyclohexanone and 1.5 equivalent of LiHMDS in the presence of 10 mol% of KI, the yield was improved to 45% yield (Entry 4). We found the more lithium enolate, the higher the yield (Entry 4 ~7). As the THF was added as a co-solvent, the yield was decreased (Entry 8).

2. Investigation on the Deprotectively Reductive Cyclization: A short cut to *trans*-2-*n*-butyl-CDHQ:

Acetal **5a** was used as the substrate for investigation on deprotection-initiated reductive cyclization. The reaction commenced coordination of the dioxolane moiety with Lewis acid $BF_3 \cdot OEt_2$, resulting in cleavage of the ethylene diol moiety and cyclization of the carbamate to give a transient *N*-acyl iminium ion. Subsequent reducing agent follows to give the CDHQ. Various combinations of the amount of $BF_3 \cdot OEt_2$ and Et_3SiH were used, and the results were described as follows (Table S2).

Table S2. Deprotectively Reductive Cyclization of acetal **5a**

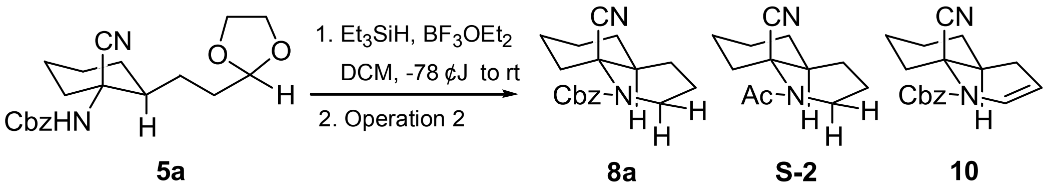

Entry[a]	BF_3OEt_2 (eq)	Et_3SiH (eq)	Operation 2	Results[b]
1	0.0	1.0	None	**5a**, 99%
2	1.0	0.0	None	**10**, 96%
3	1.0	1.0	None	**8a**, 98%
4	1.3	1.3	None	**8a**, 66%
5	2.0	2.0	AcCl (3.5 eq), Et_3N(3.5 eq), DCM, -78 ℃	**S-2**, 86%

a: All reactions were conducted on 1 to 2 mmol scale. b: Isolated yield.

Treatment with 1.0 equivalent of triethylsilane only gave starting material **5a**, indicating Lewis acid was necessary for the transformation (Entry 1). Treatment with 1.0 equivalent of $BF_3 \cdot OEt_2$ afforded enecarbamate **10** in 96% yield (Entry 2). It suggested that addition of $BF_3 \cdot OEt_2$ will result in the cleavage of the dioxolane and formation the piperidenium ion, which was deprotonated to yield enecarbamate **10**. As 1.0 equivalents of triethylsilane and 1.0 equivalents of $BF_3 \cdot OEt_2$ were added, the yield of the cyclized product **8a** could be up to 98% isolated yield (Entry 3). The result demonstrated deprotectively reductive cyclization has been realized. However, slight more addition of silane and Lewis acid (1.3 equivalents each) resulted in a lower yield of 66% (Entry 4), accompanied with some side product without the Cbz protecting group. The results implied the more than one equivalent of $BF_3 \cdot OEt_2$ might trigger the deprotection of the Cbz group. Therefore, treatment with 2.0 equivalents of triethylsilane and 2.0 equivalents of $BF_3 \cdot OEt_2$, followed by addition of excess acetylation produced acetamide **S-2** in 86% yield (Entry 5). It disclosed that deprotection the dioxolane group, reductive cyclization to form the 2nd ring structure, and switch the Cbz group to the acetyl group could be finished in one operation. Next

ketal **5b** was examined for the deprotectively reductive cyclization (Table S3).

Table S3. Deprotectively Reductive Cyclization of ketal **5b**

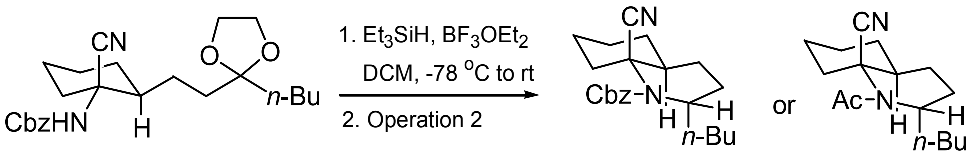

5b **8b** **9**

Entry[a]	BF_3OEt_2 (eq)	Et_3SiH (eq)	Operation 2	Results[b]
1	0.0	1.0	None	**5b**, 99%
2	1.0	0.0	None	**5b**, 98%
3	1.0	1.0	CbzCl (1.5 eq), K_2CO_3(2.8 eq), THF-H_2O 0 ℃ to rt	**5b**, 91%
4	1.3	1.3	CbzCl (1.5 eq), K_2CO_3(2.8 eq), THF-H_2O 0 ℃ to rt	**8b**, 33%
5	2.0	2.0	AcCl (3.5 eq), Et_3N(3.5 eq), DCM, -78 ℃	**9**, 81%

a: All reactions were conducted on 1 to 2 mmol scale. b: Isolated yield.

Reaction with 1.0 equivalent of triethylsilane did not proceed either (Entry 1). Different from the same treatment of acetal **5a**, which gave elimination product **10**, treatment of ketal **5b** with 1.0 equivalent of $BF_3{\cdot}OEt_2$, only gave starting material **5b**. It suggested reactivity difference of acetal **5a** from that of ketal **5b** (Entry 2). Surprisingly, as reaction with 1.0 equivalents of triethylsilane and 1.0 equivalents of $BF_3{\cdot}OEt_2$, protecting group-free product bearing with the dioxolane group was found. Thus, protection with the Cbz group was carried out, and obtained the starting material **5b** as the product in 91% yield (Entry 3). The results implied 1.0 equivalent of $BF_3{\cdot}OEt_2$, only enhanced the reactivity of the carbamate in **5b**, and facilitated the deprotection of the benzyl group with triethylsilane, rather than cleavage of the dioxolane part. Therefore, the combination, i.e. triethylsilane and $BF_3{\cdot}OEt_2$ (1.0 equivalents each) could be viewed as a novel way for deprotection of the Cbz group in presence of a ketal.

Treatment of slightly more silane and Lewis acid (1.3 equivalents each) resulted in mixture of the cyclized product and some side product without the Cbz protecting group. The results indicated the amount of $BF_3{\cdot}OEt_2$ more than 1.0 equivalent could prompt the dioxolane cleavage and the resulting cyclization, giving the protecting group-free cyclized product. After the Cbz protection, cyclized product **8b** was obtained in 33% isolated yield (Entry 4). Since the Cbz group has been removed in the cyclized conditions, direct treatment of triethylsilane and $BF_3{\cdot}OEt_2$ (2.0 equivalents each), followed by addition of excess acetylation produced *trans*-acetamide **9** in 81% yield (Entry 5).

3. Investigation on the Deprotectively *Allylative* Cyclization: A short cut to *cis*-2-allyl-8a-CDHQ:

Sterically well-defined α-aminonitrile **5a** was used as the substrate for investigation on deprotection-initiated *allylative* cyclization. Similar to the reductive cyclization, the addition of trimethylallylsilane would afford the adduct, which was assigned on basis of the spectra of the known compound. Various combinations of the amount of BF_3·OEt_2 and $Me_3SiCH_2CH=CH_2$ were used, and the results were described as follows (Table S4).

Treatment with 1.0 equivalent of trimethylallylsilane and 1.0 equivalents of BF_3·OEt_2 resulted in an inseparable mixture of the desired *cis*-2-allyl-8a-CDHQ (**4**) and enecarbamate **10** (~ 1.4 : 1) in 58% combined yield (Entry 1).[2] It clearly showed that nuclephilicity of trimethylallylsilane should be less than that of triethylsilane, suggesting more trimethylallylsilane might benefit the reaction. Addition of 3.0 equivalents of trimethylallylsilane in the presence of 1.0 equivalents of BF_3·OEt_2, did not improve the yield significantly, but gave a better ratio (~ 5 : 1, Entry 2). Addition of 1.5 equivalents of BF_3·OEt_2 in the presence of 3.0 equivalents of trimethylallylsilane gave a similar results as that in Entry 2 (Entry 3). However, reduction of the amount of BF_3·OEt_2 to 0.5 equivalents resulted in the recovery of starting material **5a** in 41% (Entry 4), which implied 1.0 equivalent of BF_3·OEt_2 might be the minimum demand. More trimethylallylsilane (6.0 equivalents) gave a 75% yield, and a little better ratio of (6.3 to 1, Entry 5). Finally, addition of 12 equivalents of trimethylallylsilane and 1.0 equivalents of BF_3·OEt_2 gave a 87% combine yield with the ratio (~ 6.6 : 1, Entry 6). Use of 3.0 equivalent of trimethylallylsilane and 1.0 equivalents of $TiCl_4$ gave 63% combined yield with a poor ratio (1.9 : 1).

Table S4. Deprotectively Allylative Cyclization of **5a**:

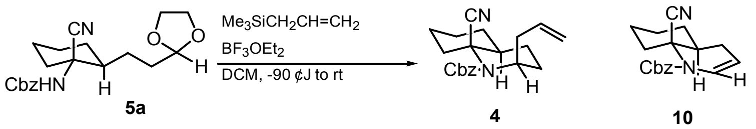

Entry[a]	BF_3OEt_2 (eq)	$AllylSiMe_3$ (eq)	Results[b]
**1**	1.0	1.0	58%, ~ 1.4 : 1
**2**	1.0	3.0	60%, ~ 5.0 : 1
**3**	1.5	3.0	60%, ~ 5.0 : 1
**4**	0.5	3.0	36%, ~ 5.3 : 1 ; **5a** : 41%
**5**	1.0	6.0	75%, ~ 6.3 : 1
**6**	1.0	12.0	87%, ~ 6.6 : 1
**7**	1.0 ($TiCl_4$)	3.0	63%, ~ 1.9 : 1

a: All reactions were conducted on 1 to 2 mmol scale. b: Isolated yield.

4. Investigation on hydrolysis of tricyclic α-aminonitrile 18

With tricyclic α-aminonitrile **18** in hand, we planned to hydrolyze the nitrile group to a carboxylic acid or carboxamide. These methods were described as follows (Table S5, Entry 1~7). First of all, common hydrochloric acid hydrolysis under reflux or microwave irradiation led to a decomposed residue (Entry 1 and 2). The conditions using AcOH/H_2SO_4 or TFA/H_2SO_4, [3,4] which worked in our synthesis for fasicularin, did not give the desired product (Entry 3 and 4). Addition of HFIP for promote the hydrolysis was not successful (Entry 5). [5] Treatment with H_2SO_4 in CH_2Cl_2 under reflux only led to recovery of starting material (Entry 6). [6] Methanol reflux conditions with 10 equivalents of TfOH yielded the same starting material recovery (Entry 7), but gave a decomposed residue in HFIP (Entry 8).

Table S5. Transformations of tricyclic α-aminonitrile **18**

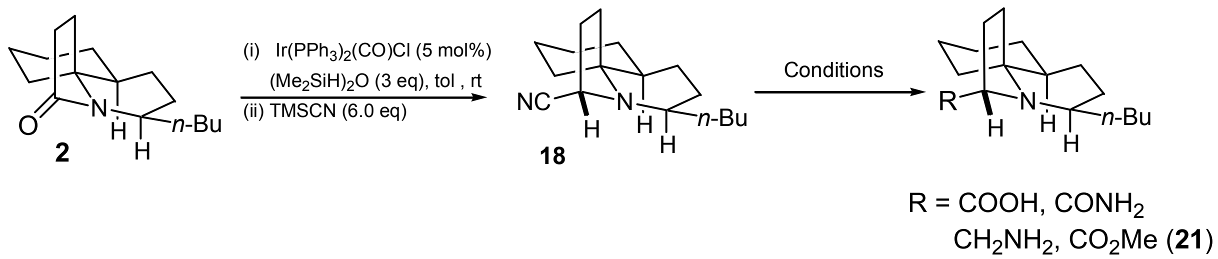

Entry[a]	Reagents	Solvents	T (°C)	Results[b]
1	HCl (6 N), AcOH	---	100 °C	dec.
2	HCl (6 N), AcOH, μ-wave	---	130 °C	dec.
3	conc H_2SO_4, AcOH	---	0 °C to rt	dec.
4	conc H_2SO_4, TFA	---	0 °C to rt	dec.
5	TFA, HFIP, H_2SO_4 (4:2:1)	---	0 °C to rt	dec.
6	conc H_2SO_4	DCM	reflux	**18**, 81% [c]
7	TfOH (10.0 eq)	MeOH	reflux	**18**, 75% [c]
8	TfOH (10.0 eq)	HFIP	reflux	dec.
9	K_2CO_3 (1.0 eq), 30% H_2O_2 (3.0 eq)	DMSO	50 °C	**2**, 63%
10	UHP (H_2NCONH_2-H_2O_2)	---	85 °C	dec.
11	$Cu(OAc)_2$ (10 mol%), NEt_2OH (5.0 eq)	THF	rt	dec.
12	$Rh(PPh_3)_3Cl$ (1.0 mol%), $CH_3CH{=}NOH$ (5.0 eq)	Tol	110 °C	**18**, 90%[c]
13	$PdCl_2$ (10 mol%), CH_3CONH_2 (4.0 eq)	THF/H_2O	rt	**2**, 95%
14	PtO_2 (10 mol%), H_2 (3.0 atm), conc HCl	EtOH	rt	dec.
15	MeOH : conc H_2SO_4 = 20 : 1 (0.17 M), 3 d	---	110 °C	**21**, 33% from **2**,

a: All reactions were conducted on 1 to 2 mmol scale. b: Isolated yield. c: crude yield.

On the other hand, common hydrogen peroxide mediated basic hydrolysis in DMSO resulted in the formation of the lactam **2** (Entry 9), while UHP-mediated

hydrolysis gave a decomposed residue (Entry 10). [7] Treatment with Cu(II)-hydroxylamine conditions did not the desired product either (Entry 11). [8] Rh-catalyzed hydration using aldoximes only gave the starting marteial recovery. (Entry 12). [9] Pd-catalyzed hydration in aqueous acetamide yielded the lactam **2** (Entry 13). [10] Moreover, PtO_2 catalyzed hydrogenation under H_2 in acidic EtOH led to a decomposed residue (Entry 14). [11]

Finally, Rychnovsky's acidic methanolysis could furnish the desired methyl ester **21** in 33% yield over two steps. [12]

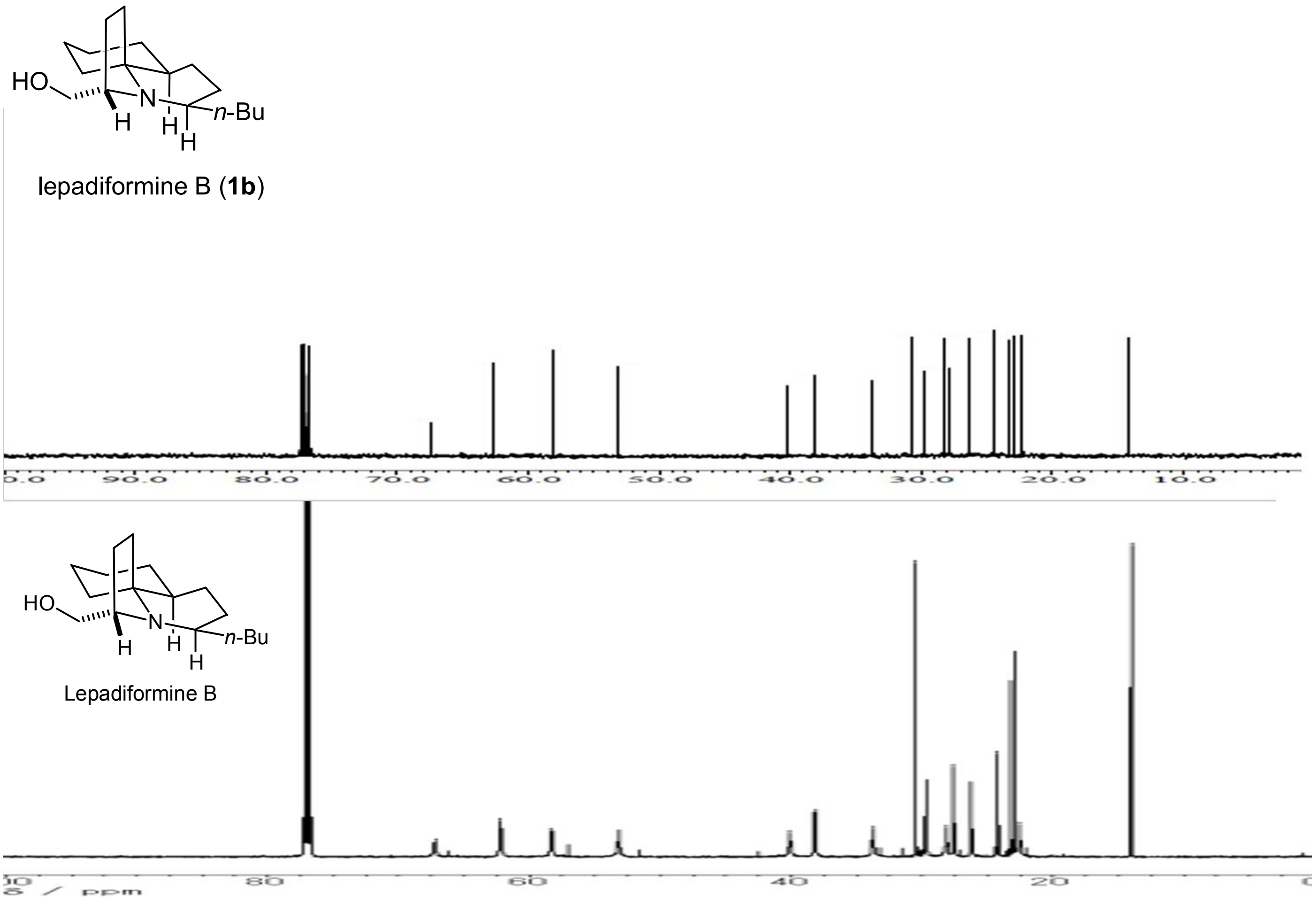

Figure S1. Comparison of the $^{13}C\{^1H\}$ NMR spectra of lepadiformine (**1b**) with a literature spectrum (lower, Morimoto et al. *Chem. Eur. J.* **2017**, *23*, 9535–9545). [13]

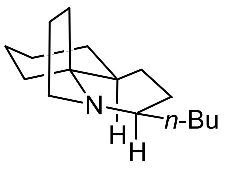

lepadiformine C (**1c**)

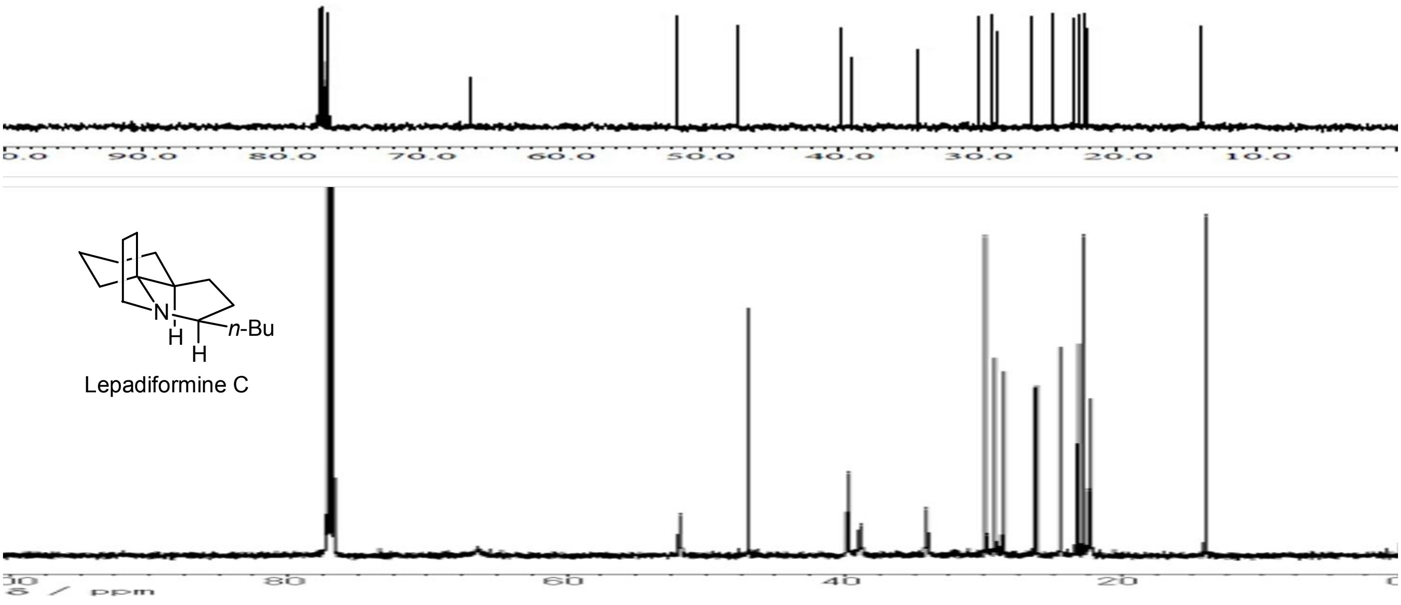

Figure S2. Comparison of the $^{13}C\{^{1}H\}$ NMR spectra of lepadiformine (**1c**) with a literature spectrum (lower, Morimoto et al. *Chem. Eur. J.* **2017**, *23*, 9535–9545). [13]

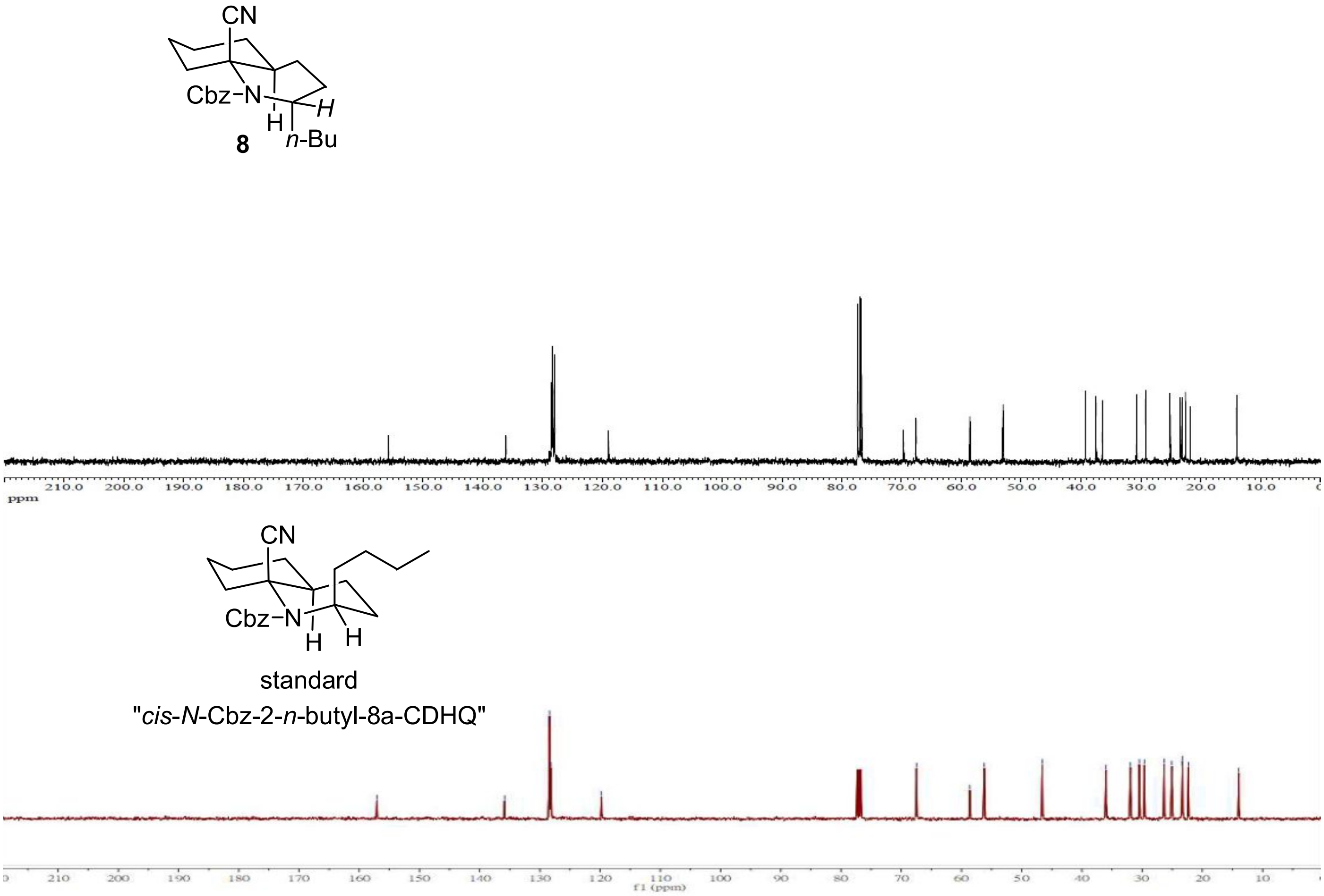

Figure S3. Comparison of the $^{13}C\{^{1}H\}$ NMR spectra of **8** with a literature spectrum of the *cis* isomer (lower one, from Chiou et al. *J. Org Chem.* **2020**, *85*, 9051–9063).[14]

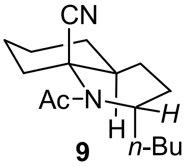

9

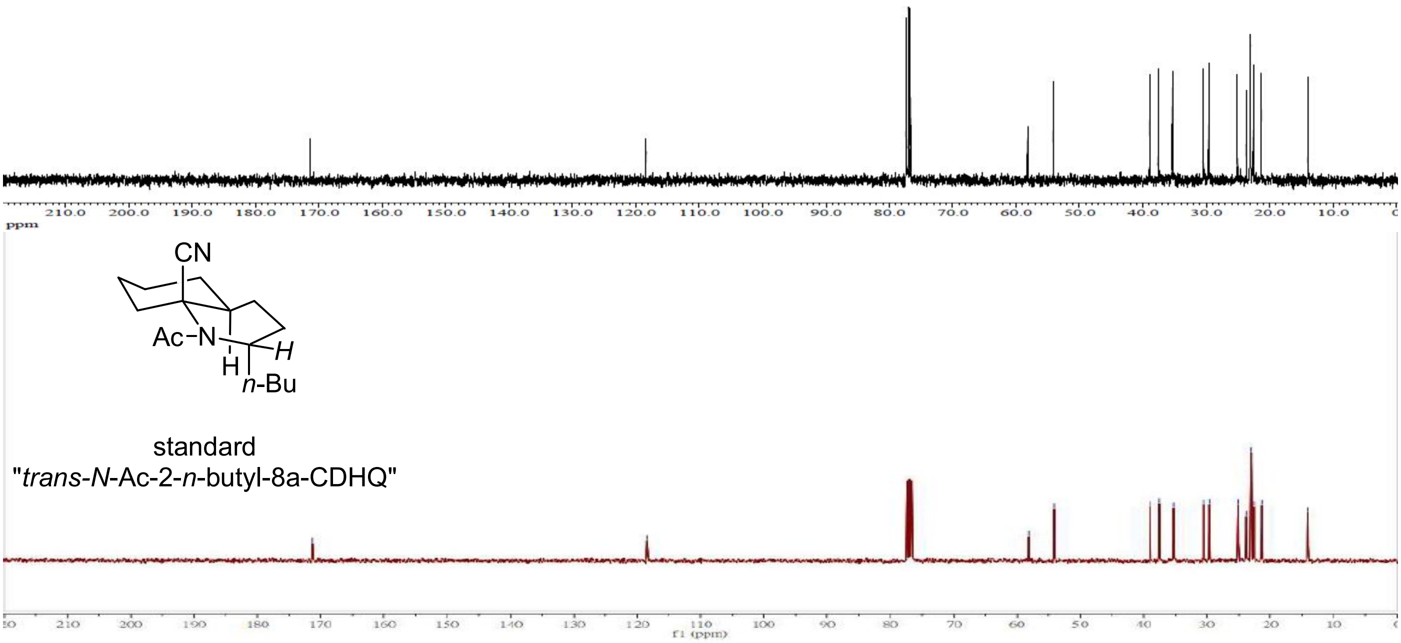

Figure S4. Comparison of the ${}^{13}C\{{}^{1}H\}$ NMR spectra of **9** with a literature spectrum of the known compound (lower one, from Chiou et al. *J. Org Chem.* **2020**, *85*, 9051–9063). [14]

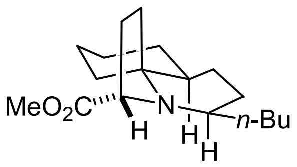

21

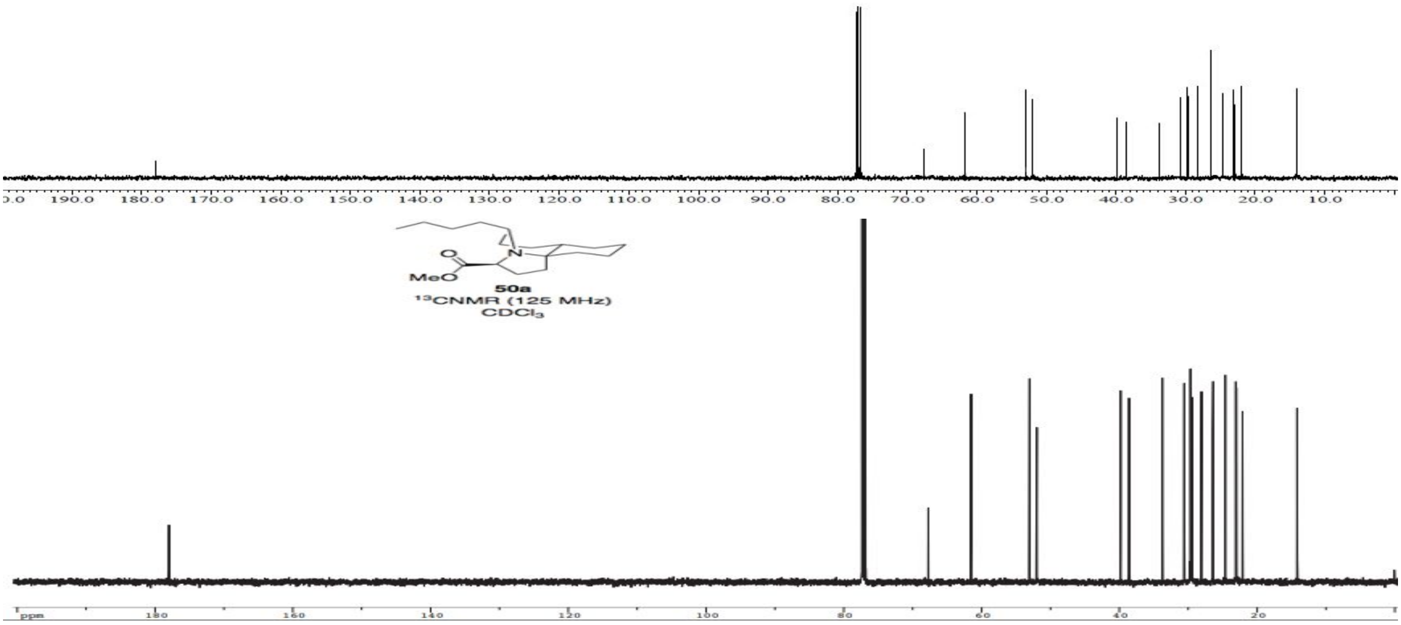

Figure S5. Comparison of the $^{13}C\{^{1}H\}$ NMR spectra of ester **21** with a literature spectrum (lower, Rychnovsky et al. *J. Org. Chem.* **2012**, *77*, 3390–3400).[12]

Table S6. Comparison of $^{13}C\{^1H\}$ NMR spectra of **1b** and **1c**

lepadiformine B	Lit.[13] values	lepadiformine C	Lit.[13] values
101 MHz, $CDCl_3$	150 MHz, $CDCl_3$	101 MHz, $CDCl_3$	150 MHz, $CDCl_3$
67.3 (C)	67.3 (C)	66.4 (C)	66.4 (C)
62.2 (CH)	62.3 (CH)	51.8 (CH)	51.9 (CH)
58.3 (CH_2)	58.3 (CH_2)	46.9 (CH_2)	46.9 (CH_2)
53.1 (CH)	53.2 (CH)	39.8 (CH)	39.7 (CH)
40.1 (CH)	40.1 (CH)	38.9 (CH_2)	38.9 (CH_2)
38.2 (CH_2)	38.2 (CH_2)	34.1 (CH_2)	34.1 (CH_2)
33.8 (CH_2)	33.8 (CH_2)	29.9 (CH_2)	29.9 (CH_2)
30.5 (CH_2)	30.5 (CH_2)	29.1 (CH_2)	29.1 (CH_2)
29.9 (CH_2)	29.8 (CH_2)	28.6 (CH_2)	28.6 (CH_2)
28.1 (CH_2)	28.2 (CH_2)	26.3 (CH_2)	26.2 (CH_2)
27.7 (CH_2)	27.6 (CH_2)	24.5 (CH_2)	24.5 (CH_2)
26.3 (CH_2)	26.3 (CH_2)	23.2 (CH_2)	23.2 (CH_2)
24.3 (CH_2)	24.2 (CH_2)	22.8 (CH_2)	22.8 (CH_2)
23.3 (CH_2)	23.2 (CH_2)	22.4 (CH_2)	22.4 (CH_2)
22.8 (CH_2)	22.9 (CH_2)	22.3 (CH_2)	22.3 (CH_2)
22.6 (CH_2)	22.6 (CH_2)	14.0 (CH_3)	14.0 (CH_3)
14.2 (CH_3)	14.1 (CH_3)	---	

Table S7. Crystal data and structure refinement for **12**. (CCDC no. 1939819)

Identification code	**12**	
Empirical formula	C21 H27 Br N2 O2	
Formula weight	419.35	
Temperature	150(2) K	
Wavelength	0.71073 Å	
Crystal system	monoclinic	
Space group	P 21	
Unit cell dimensions	a = 10.4851(5) Å	α= 90°.
	b = 7.9749(3) Å	β= 112.632(2)°.
	c = 12.6716(6) Å	γ = 90°.
Volume	977.98(8) $Å^3$	
Z	2	
Density (calculated)	1.424 Mg/m^3	
Absorption coefficient	2.119 mm^{-1}	
F(000)	436	
Crystal size	0.420 x 0.390 x 0.360 mm^3	
Theta range for data collection	3.092 to 26.397°.	
Index ranges	-13<=h<=13, -9<=k<=9, -15<=l<=15	
Reflections collected	16556	
Independent reflections	3933 [R(int) = 0.0239]	
Completeness to theta = 25.242°	99.7 %	
Absorption correction	Semi-empirical from equivalents	
Max. and min. transmission	0.9281 and 0.7426	
Refinement method	Full-matrix least-squares on F^2	
Data / restraints / parameters	3933 / 1 / 235	
Goodness-of-fit on F^2	1.030	
Final R indices [I>2sigma(I)]	R1 = 0.0307, wR2 = 0.0766	
R indices (all data)	R1 = 0.0325, wR2 = 0.0775	
Absolute structure parameter	0.369(2)	
Extinction coefficient	n/a	
Largest diff. peak and hole	0.256 and -0.260 e.$Å^{-3}$	

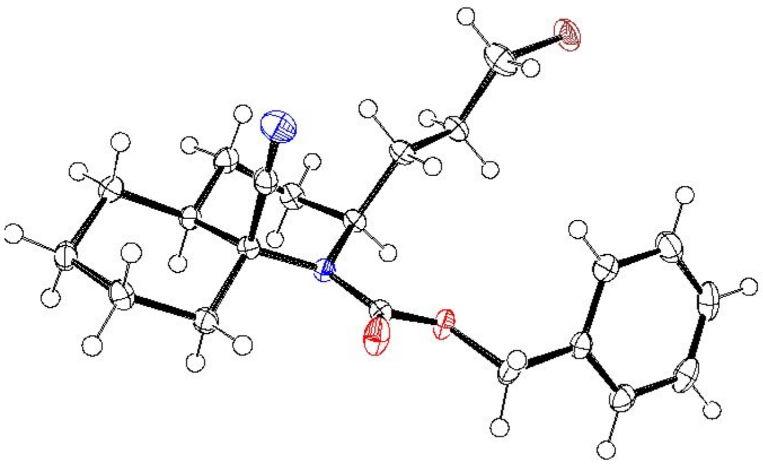

Figure S6. The ORTEP diagram of **12** with thermal ellipsoids at the 50% contour probability level (CCDC no. 1939819).

Experimental Section:

All reactions were performed under an argon atmosphere and in anhydrous solvent, unless otherwise stated. An oil bath was used for the heat source. The solvents and reagents have been dried or refined according to the literature procedures. The reaction flasks were dried in a 110 °C oven and allowed to cool to room temperature in a desiccator with drying agents and assembled under argon atmosphere. TLC analyses were visualized with UV light, iodine chamber, 10% sulfuric acid or 10% PMA solution. The crude product were purified by flash column chromatography on silica gel to give isolated yield. IR spectra were recorded on an ATR-FTIR apparatus. Melting points were recorded on a melting apparatus. The single-crystal X-ray analysis was carried out by an X-ray diffractometer, "Bruker D8 VENTURE", and the results have been reported to the Cambridge Crystallographic Data Centre to obtain the corresponding CCDC number. All NMR spectra, i.e., ^{1}H, $^{13}C\{^{1}H\}$, DEPT, gCOSY, gHSQC, and gHMBC were recorded on a 400 MHz or 600 MHz NMR spectrometer, which provided all necessary data for the full assignment of each compound. Chemical shifts (δ) are reported in ppm using residual undeuterated solvent as an internal standard. Coupling constants are described in hertz (Hz). Mass spectra were recorded on a mass spectrometer with a magnetic sector, using the electrospray ionization (ESI) or fast atom bombardment (FAB).

Microwave Irradiation Experiments: All microwave irradiation experiments were carried out in a dedicated CEM-Discover monomode microwave apparatus, operating at a frequency of 2.45 GHz with continuous irradiation power from 0 to 300 W. The reactions were carried out in 10 mL glass tubes, sealed with Teflon septum, and placed in the microwave cavity. The reactions were irradiated at the required set temperature and power for the stipulated time and then cooled to ambient temperature with air jet cooling. The reaction temperature is measured with an IR sensor, which is calibrated using DI water. The sensor is located below the microwave cavity floor to take temperature values on the bottom of the reaction vessel. When the temperature reaches the set point with the set wattage, the reaction time starts to count down as the user's input.

3,3-(ethylenedioxy)heptanol (S1)

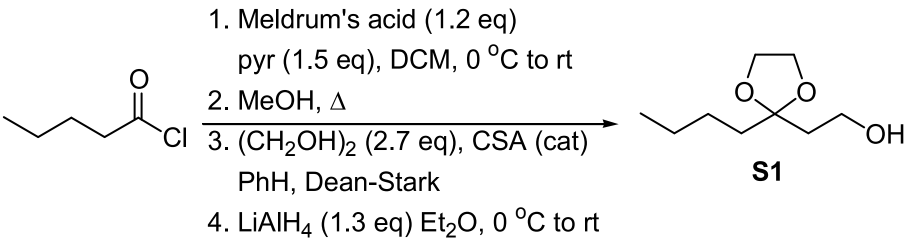

Meldrum's acid (16.3 g, 113 mmol, 1.2 eq) and pyridine (11.4 mL, 141 mmol, 1.5 eq) were dissolved in 200 mL of CH_2Cl_2 at 0 °C. Acyl chloride (11.2 mL, 94.0 mmol, 1.0 eq) was added dropwise. The solution was stirred at 0 °C for 30 minutes then warm to rt overnight. The reaction was quenched with 1N HCl solution. After separation of the organic layer, the aqueous layer was extracted with CH_2Cl_2. The combined organic layers were dried over Na_2SO_4. After removal of the solid dehydrating agent, the organic layer was concentrated under reduced pressure to give a crude product.

The crude product was dissolved in 100 mL of MeOH and heated under refluxed overnight. Upon completion of the reaction monitored by TLC analysis. The reaction mixture was concentrated to give a crude ester product.

A mixture of the crude ester, ethylene glycol (14.2 mL, 254 mmol, 2.7 eq), CSA (2.18 g, 9.40 mmol, 10 mol%) and benzene was refluxed and water was azeotropically removed with a Dean-Stark trap. Upon completion of the reaction monitored by TLC analysis, a saturated $NaHCO_3$ solution was slowly added into the reaction mixture at 0 °C, and then warmed up to rt. After separation of the organic layer, the aqueous layer was extracted with Et_2O. The combined organic layers were dried over Na_2SO_4. After removal of the solid dehydrating agent, the organic layer was concentrated under reduced pressure to give a crude product.

$LiAlH_4$ (4.64 g, 122 mmol, 1.3 eq) in dry Et_2O (150 mL) was stirred in an ice bath under Ar. To this mixture was slowly added dropwise a solution of the crude product in dry Et_2O (50 mL). Upon completion of the reaction monitored by TLC analysis, the reaction mixture was cooled down in an ice bath. The addition of water (5 mL), $NaOH_{(aq)}$ (15%, 5 mL), and water (5 mL) in sequence resulted in the formation of white solid precipitates, which were filtered off by a short celite pad to give a filtrate. The filtrate was concentrated under reduced pressure to give a crude product. The crude product was purified by a short chromatography on silica gel, EA / n-Hex as the eluant to give colorless oil **S1** (6.56 g, 37.6 mmol, 40%). R_f = 0.18 (EA / n-Hex = 1 ∶ 1); ^{1}H NMR (400 MHz, $CDCl_3$) δ 4.02-3.96 (m, 4H), 3.74 (q, J = 5.5 Hz, 2H), 2.82 (t, J = 5.7 Hz, 1H), 1.92 (t, J = 5.4 Hz, 2H), 1.70 (d, J = 2.4 Hz, 1H), 1.65-1.61 (m, 2H), 1.35-1.29 (m, 4H), 0.91-0.88 (m, 3H); $^{13}C\{^1H\}$ NMR (101 MHz, $CDCl_3$) δ 112.3 (C), 64.7 (CH_2 × 2), 58.9 (CH_2), 38.0 (CH_2), 36.8 (CH_2), 25.9 (CH_2), 22.9 (CH_2), 14.0 (CH_3); IR (cm^{-1},

film) ν_{max} = 3320, 2939, 1523, 1455, 1252, 1126; EI-HRMS (m/z) : $[M]^+$ calcd for $C_9H_{18}O_3^+$ 174.1256, found 174.1258 (Δ = 1.1 ppm).

1-bromo-3,3-(ethylenedioxy)heptane (7b)

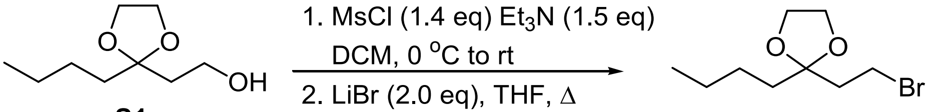

S1 7b

To a CH_2Cl_2 solution (100 mL) of the alcohol **S1** (6.56 g, 37.6 mmol, 1.0 eq), cooled in an ice bath, was added Et_3N (7.86 mL, 56.4 mmol, 1.5 eq) via a syringe followed by MsCl (4.16 mL, 52.6 mmol, 1.4 eq). The reaction was stirred room temperature for 14 h. Upon completion of the reaction monitored by TLC analysis, a saturated $NaHCO_3$ solution was slowly added. After separation of the organic layer, the aqueous layer was extracted with CH_2Cl_2. The combined organic extracts were dried over Na_2SO_4. After the removal of the solid dehydrating agent, the extracts were concentrated under reduced pressure to give a crude product. The crude product was used directly without further purification.

A solution of the crude product in dry THF (100 mL) was added to LiBr (6.53 g, 75.2 mmol, 2.0 eq) and the mixture was refluxed under Ar for 14 h. Upon completion of the reaction monitored by TLC analysis. After separation of the organic layer, the aqueous layer was extracted with Et_2O. The combined organic extracts were dried over Na_2SO_4. After the removal of the solid dehydrating agent, the extracts were concentrated under reduced pressure to give a crude product. Purification of the crude product by flash chromatography on silica gel, EA / n-Hex as the eluant to give yellow oil **7b** (7.49 g, 31.6 mmol, 84%). R_f = 0.69 (EA / n-Hex = 1 : 1); ^{1}H NMR (400 MHz, $CDCl_3$) δ 3.85 (d, J = 4.6 Hz, 4H), 3.34-3.28 (m, 2H), 2.17-2.11 (m, 2H), 1.51 (d, J = 7.3 Hz, 2H), 1.32-1.15 (m, 4H), 0.82 (d, J = 6.4 Hz, 3H); $^{13}C\{^1H\}$ NMR (101 MHz, $CDCl_3$) δ 110.6 (C), 64.8 ($CH_2 \times 2$), 40.8 (CH_2), 36.9 (CH_2), 26.8 (CH_2), 25.6 (CH_2), 22.7 (CH_2), 13.8 (CH_3); IR (cm^{-1}, film) ν_{max} = 2933, 1553, 1425, 1212, 1166; EI-HRMS (m/z) : $[M]^+$ calcd for $C_9H_{17}BrO_2^+$ 236.0412, found 236.0415 (Δ = 1.3 ppm).

General Procedure for the α-alkylation of cyclohexanone with 1-bromo-3,3-ethylenedioxyalkane

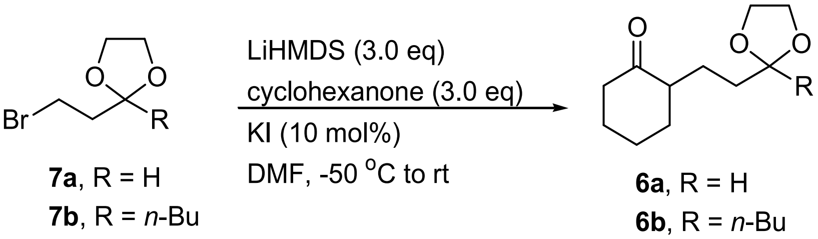

7a, R = H
7b, R = *n*-Bu

6a, R = H
6b, R = *n*-Bu

To a solution of KI (553 mg, 3.33 mmol, 10 mol%), cyclohexanone (10.4 mL, 100 mmol, 3.0 eq) in DMF (20 mL) was added LHMDS (1.0 M in THF, 100 mL, 100 mmol, 3.0 eq) at -50 °C. After being stirred for 30 min, then warmed up to rt and stirred for 1 h, a solution of bromide **7a** (3.90 mL, 33.3 mmol, 1.0 eq) in DMF (13 mL) was added slowly at -50 °C. The reaction mixture was stirred overnight, then quenched with aqueous NH_4Cl solution and extracted with EA, dried over anhydrous Na_2SO_4, and concentrated in vacuo. Purification of the crude product by flash chromatography on silica gel, EA / n-Hex as the eluant to give light yellow oil **6a** (5.31 g, 26.8 mmol, 81%).

2-(3,3-ethylenedioxyprop-1-yl)cyclohexanone (6a): light yellow oil (5.31 g, 26.8 mmol, 81%), R_f = 0.50 (EA / n-Hex = 1 :1); ^{1}H NMR (400 MHz, $CDCl_3$) δ 4.80 (t, J = 4.7 Hz, 1H), 3.93-3.77 (m, 4H), 2.36-2.21 (m, 3H), 2.10-2.06 (m, 1H), 2.03-1.96 (m, 1H), 1.90-1.80 (m, 2H), 1.69-1.56 (m, 4H), 1.39-1.24 (m, 2H); $^{13}C\{^1H\}$ NMR (101 MHz, $CDCl_3$) δ 212.8 (C), 104.4 (CH), 64.7 (CH_2 × 2), 50.3 (CH), 41.9 (CH_2), 33.9 (CH_2), 31.2 (CH_2), 27.9 (CH_2), 24.8 (CH_2), 23.7 (CH_2). These NMR data are in agreement with those reported in the literature. [1]

2-(3,3-ethylenedioxyhept-1-yl)cyclohexanone (6b): light yellow oil (1.713 g, 6.73 mmol, 72%), R_f = 0.43 (EA / n-Hex = 1 : 3); ^{1}H NMR (400 MHz, $CDCl_3$) δ 3.85 (s, 3H), 2.29 (s, 1H), 2.21 (s, 2H), 2.08-2.16 (m, 1H), 1.99-2.08 (m, 1H), 1.97 (s, 2H), 1.69-1.84 (m, 2H), 1.61-1.52 (m, 7H), 1.26-1.17 (m, 7H), 0.83 (t, J = 6.7 Hz, 3H); $^{13}C\{^1H\}$ NMR (101 MHz, $CDCl_3$) δ 213.0 (C), 111.6 (C), 64.7 (CH_2 × 2), 50.6 (CH), 41.9 (CH_2), 36.6 (CH_2), 34.2 (CH_2), 33.9 (CH_2), 27.9 (CH_2), 25.8 (CH_2), 24.7 (CH_2), 23.6 (CH_2), 22.8 (CH_2), 13.9 (CH_3); IR (cm^{-1}, film) ν_{max} = 2923, 1726, 1555, 1421, 1210, 1166; EI-HRMS (m/z) : $[M]^+$ calcd for $C_{15}H_{26}O_3^+$ 254.1882, found 254.1880 (Δ = -0.8 ppm).

General Procedure for the Strecker reaction of α-substituted cyclohexanone

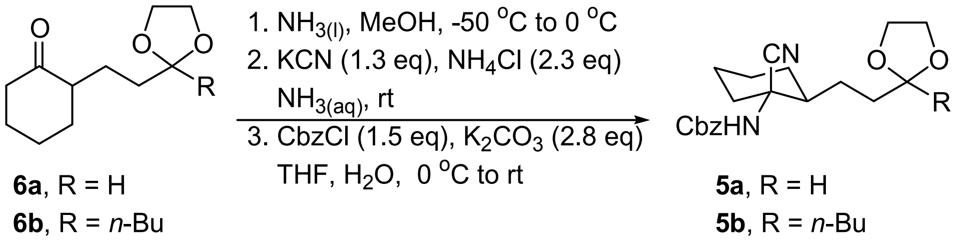

6a, R = H
6b, R = *n*-Bu

5a, R = H
5b, R = *n*-Bu

To a 250 mL three-necked flask equipped with a cold finger condenser at -50 °C in a well-ventilated hood, ammonia gas was slowly passed through the system until 30 mL of liquid ammonia had been formed. Under slow flow rate of ammonia atmosphere, the condenser was replaced with an addition funnel containing **6a** (1.838 g, 9.27 mmol, 1.0 eq) dissolved in 20 mL of MeOH. The solution was added to the liquid ammonia, and the funnel was rinsed with a little MeOH. The resulting NH_3-MeOH solution was

warmed up to 0 ℃ and stirred for 1 h. To the solution was added KCN (785 mg, 12.1 mmol, 1.3 eq), NH_4Cl (1.140 g, 21.3 mmol, 2.3 eq) and aqueous ammonia (28%, 20 mL) in sequence. After addition had been complete, the outlets of the reaction flask were equipped with a drying tube, and the solution was stirred room temperature overnight. Small amount of water was added to dissolve the solid precipitate, and the resulting reaction mixture was extracted with CH_2Cl_2. The combined organic layers were dried over anhydrous Na_2SO_4, filtered and concentrated in vacuo to give a crude product. The crude product was used directly without further purification.

To a THF solution (18 mL) of crude product, K_2CO_3 (3.59 g, 26.0 mmol, 2.8 eq) in water (9 mL) was added and the solution is stirred for 10 min in an ice bath. CbzCl (2.00 mL, 13.9 mmol, 1.5 eq) was added slowly via a syringe. After the addition had been finished, the ice bath was removed and the solution was stirred room temperature overnight. Separated from the reaction mixture, the aqueous layer was subjected to extraction with EA. The combined organic layers were dried over anhydrous Na_2SO_4, filtered and concentrated in vacuo to give a crude product. Purification of the crude product by flash chromatography on silica gel, EA / n-Hex as the eluant to give colorless oil **5a** (3.02 g, 8.43 mmol, 91%).

***E*-2-benzyloxycarbonylamino-2-cyano-1-(3,3-ethylenedioxypropyl)cyclohexane** (**5a**): colorless oil (3.02 g, 8.43 mmol, 91%), R_f = 0.31 (EA / n-Hex = 1 ∶ 1); ^1H NMR (400 MHz, $CDCl_3$) δ 7.36-7.30 (m, 5H), 5.83 (s, 1H), 5.20-5.08 (m, 2H), 4.86-4.84 (m, 1H), 3.98-3.90 (m, 2H), 3.84-3.79 (m, 2H), 2.81 (d, *J* = 12.4 Hz, 1H), 1.94-1.86 (m, 3H), 1.75-1.72 (m, 2H), 1.64-1.53 (m, 3H), 1.43-1.31 (m, 3H), 1.28-1.16 (m, 1H); $^{13}C\{^1H\}$ NMR (101 MHz, $CDCl_3$) δ 154.2 (C), 136.0 (C), 128.5 (CH × 2), 128.2 (CH), 128.1 (CH × 2), 118.0 (C), 104.1 (CH), 67.0 (CH_2), 64.8 (CH_2), 64.7 (CH_2), 57.1 (C), 44.0 (CH), 36.2 (CH_2), 30.3 (CH_2), 29.0 (CH_2), 25.0 (CH_2), 24.6 (CH_2), 22.4 (CH_2); IR (cm^{-1}, film) ν_{max} = 3320, 2939, 2865, 2238, 1726, 1523, 1455, 1252, 1126; EI-HRMS (m/z) ∶ $[M]^+$ calcd for $C_{20}H_{26}N_2O_4^+$ 358.1893, found 358.1898 (Δ = 1.4 ppm).

***E*-2-benzyloxycarbonylamino-2-cyano-1-(3,3-ethylenedioxyheptyl)cyclohexane** (**5b**): colorless oil (2.34 g, 5.64 mmol, 84%), R_f = 0.52 (EA / n-Hex = 1 ∶ 2); ^1H NMR (400 MHz, $CDCl_3$) δ 7.40-7.26 (m, 5H), 5.81 (d, *J* = 6.9 Hz, 1H), 5.14-5.07 (m, 2H), 3.92-3.85 (m, 4H), 2.76 (d, *J* = 11.8 Hz, 1H), 1.89-1.78 (m, 3H), 1.72-1.65 (m, 2H), 1.57-1.46 (m, 5H), 1.44-1.18 (m, 8H), 0.87 (t, *J* = 6.4 Hz, 3H); $^{13}C\{^1H\}$ NMR (101 MHz, $CDCl_3$) δ 154.1 (C), 135.8 (C), 128.4 (CH), 128.2 (CH), 128.1 (CH), 128.0 (CH × 2), 118.0 (C), 111.7 (C), 66.8 (CH_2), 64.5 (CH_2 × 2), 64.5 (CH_2), 57.0 (C), 44.3 (CH), 36.6 (CH_2), 33.8 (CH_2), 28.7 (CH_2), 25.9 (CH_2), 24.9 (CH_2), 24.5 (CH_2), 22.7 (CH_2), 22.3 (CH_2), 13.9 (CH_3); IR (cm^{-1}, film) ν_{max} = 3320, 2939, 2865, 2238, 1726, 1523, 1455, 1252, 1126; EI-HRMS (m/z) ∶ $[M]^+$ calcd for $C_{24}H_{34}N_2O_4^+$ 414.2519, found 414.2518 (Δ = -0.2 ppm).

The General Procedure of Deprotectively Reductive Cyclization:

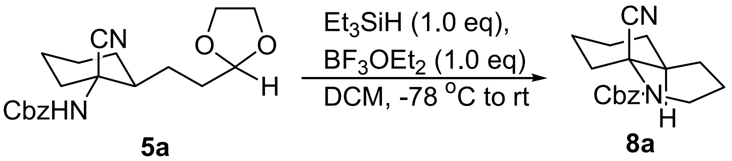

To a CH_2Cl_2 solution (10 mL) of Et_3SiH (0.163 mL, 1.018 mmol, 1.0 eq) and BF_3OEt_2 (0.129 mL, 1.018 mmol, 1.0 eq) at -78 °C was added dropwise CH_2Cl_2 solution (5 mL) of **5a** (365 mg, 1.018 mmol, 1.0 eq) at -78 °C. The cooling bath was removed and the reaction mixture was stirred at rt. Upon completion of the reaction monitored by TLC analysis, a saturated $NaHCO_3$ solution was slowly added into the reaction mixture at 0 °C, and then warmed up to room temperature. After separation of the organic layer, the aqueous layer was extracted with CH_2Cl_2. The combined organic layers were dried over Na_2SO_4. After removal of the solid dehydrating agent, the organic layer was concentrated under reduced pressure to give a crude product. Purification of the crude product by flash chromatography on silica gel, EA / n-Hex as the eluant to give colorless oil **8a** (299 mg, 1.002 mmol, 98%).

***rel*-(4a*R*,8a*S*)-*N*-Benzyloxycarbonyl-8a-cyanodecahydroquinoline (8a):** R_f = 0.33 (EA / n-Hex = 1 : 5); ^{1}H NMR (400 MHz, $CDCl_3$) δ 7.38-7.32 (m, 5H), 5.15 (s, 2H), 3.66-3.59 (m, 2H), 3.14 (d, J = 12.9 Hz, 1H), 1.84-1.76 (m, 3H), 1.71-1.62 (m, 4H), 1.58-1.47 (m, 4H), 1.36-1.20 (m, 1H); $^{13}C\{^1H\}$ NMR (101 MHz, $CDCl_3$) δ 155.3 (C), 136.0 (C), 128.4 (CH × 2), 128.1 (CH), 128.0 (CH × 2), 118.3 (C), 67.5 (CH_2), 61.1 (C), 41.94 (CH), 41.87 (CH_2), 35.0 (CH_2), 30.2 (CH_2), 25.7 (CH_2), 24.9 (CH_2), 23.3 (CH_2), 22.5 (CH_2). The NMR data are in agreement with those reported in the literature.[14]

***rel*-(2*R*,4a*R*,8a*S*)-*N*-Benzyloxycarbonyl-2-butyl-8a-cyanodecahydroquinoline (8b):**

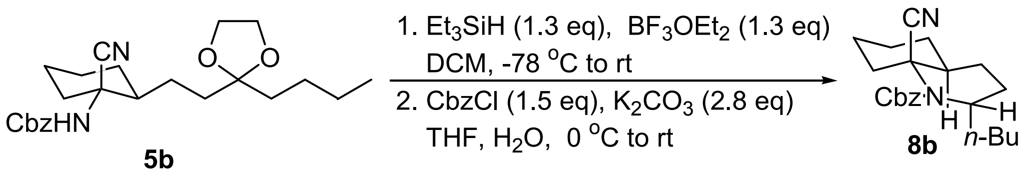

To a CH_2Cl_2 solution (10 mL) of Et_3SiH (1.171 mL, 7.33 mmol, 1.3 eq) and BF_3OEt_2 (0.929 mL, 7.33 mmol, 1.3 eq) at -78 °C was added dropwise CH_2Cl_2 solution (10 mL) of **5b** (2.34 g, 5.64 mmol, 1.0 eq) at -78 °C. The cooling bath was removed and the reaction mixture was stirred room temperature. Upon completion of the reaction monitored by TLC analysis, a saturated $NaHCO_3$ solution was slowly added into the reaction mixture at 0 °C, and then warmed up to room temperature. After separation of the organic layer, the aqueous layer was extracted with CH_2Cl_2. The combined organic

layers were dried over Na_2SO_4. After removal of the solid dehydrating agent, the organic layer was concentrated under reduced pressure to give a crude product.

To a THF solution (20 mL) of crude product, K_2CO_3 (2.18 g, 15.8 mmol, 2.8 eq) in water (10 mL) was added and the solution is stirred for 10 min in an ice bath. CbzCl (1.208 mL, 8.46 mmol, 1.5 eq) is added slowly via a syringe. After the addition had been finished, the ice bath is removed and the solution is stirred room temperature overnight. Separated from the reaction mixture, the aqueous layer was subjected to extraction with EA. The combined organic layers were dried over anhydrous Na_2SO_4, filtered and concentrated in vacuo to give a crude product. Purification of the crude product by flash chromatography on silica gel, EA / n-Hex as the eluant to give colorless oil **8b** (660 mg, 1.862 mmol, 33%). R_f = 0.62 (EA / n-Hex = 1 : 2); ^{1}H NMR (400 MHz, $CDCl_3$) δ 7.40-7.32 (m, 5H), 5.19 (m, 2H), 4.15 (d, J = 6.6 Hz, 1H), 3.27-3.24 (m, 1H), 2.11-2.04 (m, 2H), 1.78-1.71 (m, 5H), 1.69-1.61 (m, 3H), 1.57-1.49 (m, 3H), 1.31-1.20 (m, 5H), 0.88-0.84 (m, 3H); $^{13}C\{^1H\}$ NMR (101 MHz, $CDCl_3$) δ 155.7 (C), 136.1 (C), 128.5 (CH × 2), 128.4 (CH), 128.3 (CH), 128.0 (CH), 119.0 (C), 67.6 (CH_2), 58.6 (C), 53.0 (CH), 39.2 (CH), 37.5 (CH_2), 36.5 (CH_2), 30.7 (CH_2), 29.2 (CH_2), 25.1 (CH_2), 23.4 (CH_2), 23.1 (CH_2), 22.5 (CH_2), 21.7 (CH_2), 14.0 (CH_3); IR (cm^{-1}, film) ν_{max} = 2936, 2864, 2229, 1711, 1457, 1382, 1281, 1242; EI-HRMS (m/z) : $[M]^+$ calcd for $C_{22}H_{30}N_2O_2^+$ 354.2307, found 354.2308 (Δ = 0.3 ppm).

The General Procedure of Deprotectively Reductive Cyclization and Acetylation:

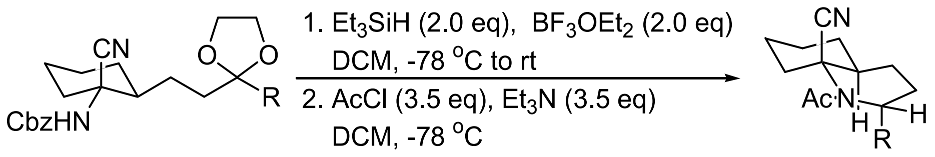

5a, R = H
5b, R = *n*-Bu

S2, R = H,
9, R = *n*-Bu

To a CH_2Cl_2 solution (10 mL) of Et_3SiH (0.162 mL, 1.014 mmol, 2.0 eq) and BF_3OEt_2 (0.128 mL, 1.014 mmol, 2.0 eq) at -78 ℃ was added dropwise CH_2Cl_2 solution (5 mL) of **5b** (210 mg, 0.507 mmol, 1.0 eq) at -78 ℃. The cooling bath was removed and the reaction mixture was stirred room temperature. Upon completion of the reaction monitored by TLC analysis, a saturated $NaHCO_3$ solution was slowly added into the reaction mixture at 0 ℃, and then warmed up to rt. After separation of the organic layer, the aqueous layer was extracted with CH_2Cl_2. The combined organic layers were dried over Na_2SO_4. After removal of the solid dehydrating agent, the organic layer was concentrated under reduced pressure to give a crude product.

To a CH_2Cl_2 solution (10 mL) of acetyl chloride (0.126 mL, 1.775 mmol, 3.5 eq) at -78 ℃ was slowly added Et_3N (0.247 mL, 1.775 mmol, 3.5 eq), and the solution was

stirred for another 10 min at -78 °C. To the freshly prepared acetylating reagent was cannulated the crude amine in CH_2Cl_2 solution (5 mL). The solution was stirred for 2 h at -78 °C. Upon completion of the reaction monitored by TLC analysis, a saturated $NaHCO_3$ solution was slowly added. After the separation of the organic layer, the aqueous layer was extracted with CH_2Cl_2. The combined organic extracts were dried over Na_2SO_4. After the removal of the solid dehydrating agent, the organic layer was concentrated under reduced pressure to give a crude product. Purification of the crude product by flash chromatography on silica gel, EA / n-Hex as the eluant to give yellow oil **9** (108 mg, 0.412 mmol, 81%).

***rel*-(2*R*,4a*R*,8a*S*)-*N*-Acetyl-2-butyl-8a-cyanodecahydroquinoline (9):** R_f = 0.28 (EA / n-Hex = 1 : 3); ^{1}H NMR (400 MHz, $CDCl_3$) δ 3.69 (t, *J* = 3.3 Hz, 1H), 3.31 (d, *J* = 12.9 Hz, 1H), 2.15-2.03 (m, 4H), 1.88-1.82 (m, 1H), 1.76-1.59 (m, 7H), 1.55-1.48 (m, 2H), 1.36-1.23 (m, 5H), 1.15-1.05 (m, 1H), 0.92-0.85 (m, 3H); $^{13}C\{^1H\}$ NMR (101 MHz, $CDCl_3$) δ 171.3 (C), 118.4 (C), 58.2 (C), 54.1 (CH), 38.9 (CH), 37.6 (CH_2), 35.3 (CH_2), 30.5 (CH_2), 29.6 (CH_2), 25.1 (CH_2), 23.7 (CH_3), 23.0 (CH_2 × 2), 22.6 (CH_2), 21.3 (CH_2), 13.9 (CH_3). These NMR data are in agreement with those reported in the literature. [14]

***trans*-*N*-Acetyl-8a-cyanodecahydroquinoline (S2):** white solid, (188 mg, 0.911 mmol, 86%); mp = 63−64 °C, (lit. [14] mp = 62−64 °C); R_f = 0.43 (EA); ^{1}H NMR (400 MHz, $CDCl_3$) δ 3.46 (dd, *J* = 7.6, 4.5 Hz, 2H), 3.19 (d, *J* = 12.9 Hz, 1H), 2.13 (s, 3H), 1.92-1.83 (m, 2H), 1.81-1.71 (m, 4H), 1.69-1.52 (m, 4H), 1.41-1.27 (m, 2H); $^{13}C\{^1H\}$ NMR (101 MHz, $CDCl_3$) δ 170.5 (C), 117.8 (C), 59.9 (C), 41.7 (CH_2), 40.2 (CH), 33.5 (CH_2), 29.6 (CH_2), 24.7 (CH_2), 23.9 (CH_2), 23.7 (CH_3), 22.8 (CH_2), 21.9 (CH_2). The NMR data are in agreement with those reported in the literature. [14]

The Procedure of Deprotectively Allylative Cyclization and Subsequent Hydration:

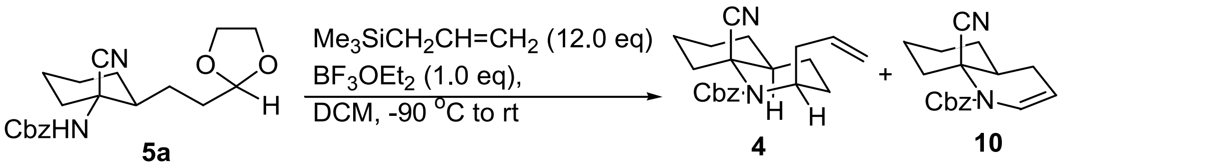

To a CH_2Cl_2 solution (5 mL) of allyltrimethylsilane (1.659 mL, 10.4 mmol, 12.0 eq) and BF_3•OEt_2 (0.107 mL, 0.870 mmol, 1.0 eq) at -90 °C was added dropwise CH_2Cl_2 solution (5 mL) of **5a** (312 mg, 0.870 mmol, 1.0 eq) at -90 °C. The cooling bath was removed and the reaction mixture was stirred room temperature. Upon completion of the reaction monitored by TLC analysis, a saturated $NaHCO_3$ solution was slowly added into the reaction mixture at 0 °C, and then warmed up to room temperature. After separation of the organic layer, the aqueous layer was extracted with CH_2Cl_2. The

combined organic layers were dried over Na_2SO_4. After removal of the solid dehydrating agent, the organic layer was concentrated under reduced pressure to give a crude product. Purification of the crude product by flash chromatography on silica gel, EA / n-Hex as the eluant to give colorless oil mixture product (240 mg, 0.756 mmol, 87%, **4** : **10** = 1 ∶ 0.15), R_f = 0.44 (EA / n-Hex = 1 ∶ 5).

***rel*-(2*S*,4a*S*,8a*R*)-*N*-Benzyloxycarbonyl-2-allyl-8a-cyanodecahydroquinoline (4):** white solid, mp = 57−60 °C, (lit. [14] mp = 58−60 °C); ^{1}H NMR (400 MHz, $CDCl_3$) δ 7.38-7.32 (m, 5H), 5.62-5.51 (m, 1H), 5.18 (d, J = 12.0 Hz, 1H), 5.11 (d, J = 12.0 Hz, 1H), 5.02 (m, 2H), 4.30 (t, J = 5.1 Hz, 1H), 3.53 (d, J = 12.9 Hz, 1H), 2.68-2.59 (m, 1H), 2.37-2.30 (m, 1H), 1.84-1.75 (m, 3H), 1.72-1.60 (m, 5H), 1.52-1.43 (m, 1H), 1.41-1.28 (m, 3H); $^{13}C\{^1H\}$ NMR (101 MHz, $CDCl_3$) δ 156.9 (C), 135.7 (C), 134.9 (CH), 128.5 (CH × 2), 128.3 (CH × 2), 128.3 (CH), 119.9 (C), 117.8 (CH_2), 67.6 (CH_2), 58.7 (C), 55.3 (CH), 46.5 (CH), 36.6 (CH_2), 35.9 (CH_2), 30.5 (CH_2), 26.1 (CH_2), 25.1 (CH_2), 23.3 (CH_2), 23.2 (CH_2). These NMR data are in agreement with those reported in the literature. [14]

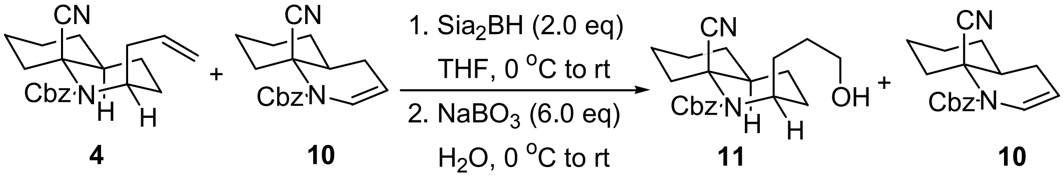

To a BH_3-SMe_2 solution (2.0 M in THF, 3.79 mL, 7.58 mmol, 2.0 eq) at -30 ℃ was slowly added 2-methyl-2-butene (1.610 mL, 15.2 mmol, 4.0 eq) via a syringe. The solution was stirred for 2 h in an ice bath. To the freshly prepared Sia_2BH solution was transferred by cannulation into a THF solution (20 mL) of a mixture from previous deprotectively allylative cyclization (1.507 g, 4.55 mmol, 1.0 eq, the ratio of **4** : **10** around 5 ∶ 1). After addition was finished, the ice bath was removed and the reaction mixture was stirred for 1 h. Upon completion of the reaction monitored by TLC analysis, the reaction was quenched with water (20 mL), followed by $NaBO_3$•$4H_2O$ (3.50 g, 22.7 mmol, 6.0 eq), and the resulting solution was stirred overnight. Separated from the reaction mixture, the aqueous layer was subjected to extraction with EA. The combined organic layers were dried over anhydrous Na_2SO_4, filtered, and concentrated in vacuo to give a crude product. Purification of the crude product by flash chromatography on silica gel, EA / n-Hex as the eluant to give white solid **11** (1.270 g, 3.56 mmol, 78%) and white solid **10** (210 mg, 0.709 mmol, 16%).

***trans*-*N*-Benzyloxycarbonyl-2,3-didehydro-8a-cyanodecahydroquinoline (10):** White solid, (210 mg, 0.709 mmol, 16%), mp = 81−84 °C, (lit. [14] mp = 82−84 °C); R_f = 0.44 (EA / n-Hex = 1 ∶ 5); ^{1}H NMR (400 MHz, $CDCl_3$) δ 7.39-7.31 (m, 5H), 6.78-6.72 (m, 1H), 5.25-5.20 (m, 2H), 5.03-4.99 (m, 1H), 3.48 (d, J = 12.9 Hz, 1H), 2.06

(dd, J = 17.4, 11.5 Hz, 1H), 1.96-1.89 (m, 1H), 1.84-1.67 (m, 5H), 1.60-1.51 (m, 1H), 1.44-1.26 (m, 2H); $^{13}C\{^1H\}$ NMR (101 MHz, $CDCl_3$) δ 152.9 (C), 135.4 (C), 128.6 (CH × 2), 128.4 (CH), 128.2 (CH × 2), 125.3 (CH), 117.8 (C), 106.1 (CH), 68.2 (CH_2), 59.2 (C), 42.2 (CH), 34.7 (CH_2), 30.0 (CH_2), 26.2 (CH_2), 24.9 (CH_2), 23.1 (CH_2). These NMR data are in agreement with those reported in the literature. [14]

***rel*-(2*S*,4a*S*,8a*R*)-*N*-Benzyloxycarbonyl-2-(3-hydroxypropyl)-8a-cyanodecahydro-quinoline (11):** White solid, (1.270 g, 3.56 mmol, 78%), mp = 87−88 °C, (lit. [14] mp = 86−88 °C); R_f = 0.38 (pure EA); ^1H NMR (400 MHz, $CDCl_3$) δ 7.38-7.29 (m, 5H), 5.80 (d, J = 12.0 Hz, 1H), 5.22 (d, J = 12.0 Hz, 1H), 4.25 (d, J = 9.8 Hz, 1H), 3.51-3.46 (m, 3H), 1.99-1.90 (m, 2H), 1.77-1.58 (m, 9H), 1.54-1.42 (m, 3H), 1.40-1.24 (m, 5H); $^{13}C\{^1H\}$ NMR (101 MHz, $CDCl_3$) δ 157.1 (C), 135.7 (C), 128.5 (CH × 3), 128.3 (CH × 2), 119.9 (C), 67.6 (CH_2), 62.1 (CH_2), 58.7 (C), 55.7 (CH), 46.5 (CH), 36.0 (CH_2), 30.5 (CH_2), 30.2 (CH_2), 28.5 (CH_2), 26.4 (CH_2), 25.0 (CH_2), 23.3 (CH_2 × 2). These NMR data are in agreement with those reported in the literature. [14]

***rel*-(2*S*,4a*S*,8a*R*)-*N*-Benzyloxycarbonyl-2-(3-bromopropyl)-8a-cyanodecahydro-quinoline (12):**

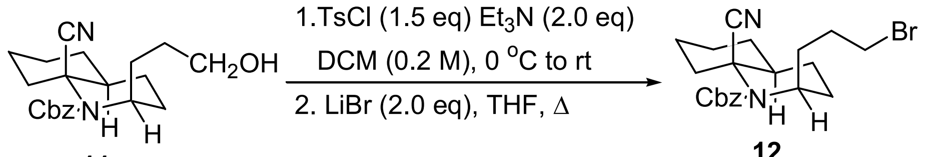

11 12

To a CH_2Cl_2 solution (20 mL) of the alcohol **11** (822 mg, 2.31 mmol, 1.0 eq), cooled in an ice bath, was added Et_3N (0.644 mL, 4.62 mmol, 2.0 eq) via a syringe followed by TsCl (661 mg, 3.47 mmol, 1.5 eq). The reaction was stirred at rt for 18 h. Upon completion of the reaction monitored by TLC analysis, a saturated $NaHCO_3$ solution was slowly added. After separation of the organic layer, the aqueous layer was extracted with CH_2Cl_2. The combined organic extracts were dried over Na_2SO_4. After the removal of the solid dehydrating agent, the extracts were concentrated under reduced pressure to give a crude product. The crude product was used directly without further purification.

A solution of the crude product in dry THF (20 mL) was added to LiBr (401 mg, 4.62 mmol, 2.0 eq) and the mixture was refluxed under Ar for 15 h. Upon completion of the reaction monitored by TLC analysis. After separation of the organic layer, the aqueous layer was extracted with Et_2O. The combined organic extracts were dried over Na_2SO_4. After the removal of the solid dehydrating agent, the extracts were concentrated under reduced pressure to give a crude product. Purification of the crude product by flash chromatography on silica gel, EA / n-Hex as the eluant to give the titled product as yellow solid (810 mg, 1.932 mmol, 84%).

The bromide **12** was recrystallized with CH_2Cl_2-hexane to afford a needle crystal, CCDC no. 1939819; mp = 96 – 99 ℃; R_f = 0.33 (EA / n-Hex = 1 : 3); ^{1}H NMR (400 MHz, $CDCl_3$) δ 7.39-7.31 (m, 5H), 5.24 (d, J = 12.0 Hz, 1H), 5.08 (d, J = 12.0 Hz, 1H), 4.23 (d, J = 3.1 Hz, 1H), 3.52 (d, J = 13.3 Hz, 1H), 3.18 (t, J = 6.8 Hz, 2H), 2.06-1.96 (m, 1H), 1.78-1.71 (m, 6H), 1.67-1.56 (m, 5H), 1.55-1.46 (m, 2H), 1.42-1.25 (m, 4H); ^{13}C{^{1}H} NMR (101 MHz, $CDCl_3$) δ 156.8 (C), 135.7 (C), 128.7 (CH × 2), 128.6 (CH), 128.5 (CH × 2), 119.7 (C), 67.7 (CH_2), 58.8 (C), 55.3 (CH), 46.5 (CH), 36.0 (CH_2), 32.5 (CH_2), 31.1 (CH_2), 30.9 (CH_2), 30.5 (CH_2), 26.6 (CH_2), 25.0 (CH_2), 23.4 (CH_2 × 2); IR (cm^{-1}, film) ν_{max} = 2866, 1717, 1441, 1353, 1266, 1251, 1152, 1122; EI-HRMS (m/z) : $[M]^+$ calcd for $C_{21}H_{27}BrN_2O_2^+$ 418.1256, found 418.1251 (Δ = -1.2 ppm).

***rel*-(2*S*,4a*S*,8a*R*)-*N*-Benzyloxycarbonyl-2-(3-oxoxypropyl)-8a-cyanodecahydro-quinoline (13):**

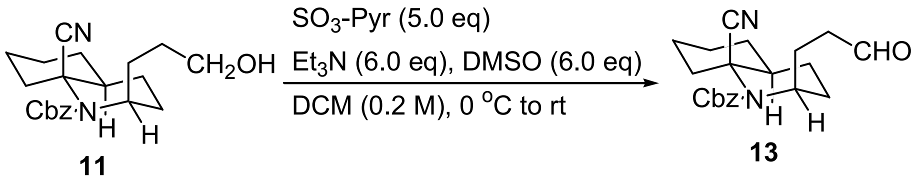

To a CH_2Cl_2 solution (10 mL) of **11** (422 mg, 1.184 mmol, 1.0 eq), DMSO (0.503 mL, 7.10 mmol, 6.0 eq), SO_3•Py (942 mg, 5.92 mmol, 5.0 eq), and NEt_3 (0.990 mL, 7.10 mmol, 6.0 eq) at 0 ℃. The resulting mixture was stirred at room temperature for 2 h. Upon completion of the reaction monitored by TLC analysis, a saturated NH_4Cl solution was slowly added into the reaction mixture at 0 ℃, and then warmed up to rt. After separation of the organic layer, the aqueous layer was extracted with CH_2Cl_2. The combined organic layers were dried over Na_2SO_4. After removal of the solid dehydrating agent, the organic layer was concentrated under reduced pressure to give a crude product. Purification of the crude product by flash chromatography on silica gel, EA / n-Hex as the eluant to give colorless oil **13** (275 mg, 0.776 mmol, 66%), R_f = 0.28 (EA / n-Hex = 1 : 3); ^{1}H NMR (400 MHz, $CDCl_3$) δ 9.48 (s, 1H), 7.37-7.30 (m, 5H), 5.23 (d, J = 12.0 Hz, 1H), 5.09 (d, J = 12.0 Hz, 1H), 4.26 (d, J = 7.0 Hz, 1H), 3.51 (d, J = 12.9 Hz, 1H), 2.28-2.09 (m, 3H), 1.94-1.85 (m, 1H), 1.81-1.58 (m, 8H), 1.55-1.46 (m, 1H), 1.43-1.38 (m, 1H), 1.36-1.24 (m, 2H); ^{13}C{^{1}H} NMR (101 MHz, $CDCl_3$) δ 201.0 (CH), 156.7 (C), 135.6 (C), 128.6 (CH × 3), 128.5 (CH × 2), 119.7 (C), 67.7 (CH_2), 58.7 (C), 55.1 (CH), 46.5 (CH), 41.1 (CH_2), 36.0 (CH_2), 30.5 (CH_2), 26.6 (CH_2), 25.0 (CH_2), 24.6 (CH_2), 23.3 (CH_2), 23.3 (CH_2). These NMR data are in agreement with those reported in the literature. [14]

***rel*-(2*R*,4a*S*,8a*R*)-*N*-Benzyloxycarbonyl-2-(3-butenyl)-8a-cyanodecahydro-quinoline (14):**

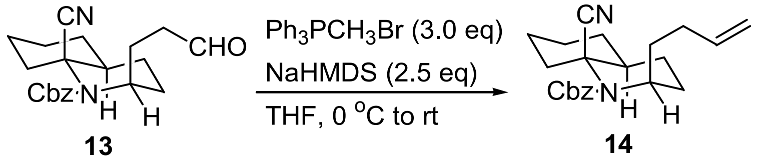

To a THF solution (15 mL) of methyltriphenyl-phosphonium bromide (1.663 g, 4.66 mmol, 3.0 eq) at an ice bath was slowly added NaHMDS solution (1.0 M in THF, 3.88 mL, 3.88 mmol, 2.5 eq) via a syringe. The reaction mixture was stirred at 0 °C for 1 h. To the freshly prepared Wittig reagent at the ice bath was added a THF solution (15 mL) of aldehyde **13** (550 mg, 1.552 mmol, 1.0 eq) and warmed up gradually to rt. Upon completion of the reaction monitored by TLC analysis, the reaction was quenched with saturated NH_4Cl. After the separation of the organic layer, the aqueous layer was extracted with EA. The combined organic layers were dried over Na_2SO_4. After the removal of the solid dehydrating agent, the organic layer was concentrated under reduced pressure to give a crude product. Purification of the crude product by flash chromatography on silica gel, EA / n-Hex as the eluant to give white solid **14** (466 mg, 1.322 mmol, 85%), mp = 93−95 °C, (lit. [14] mp = 93−95 °C); R_f = 0.58 (EA / n-Hex = 1 : 3); ^{1}H NMR (400 MHz, $CDCl_3$) δ 7.40-7.30 (m, 5H), 5.65-5.56 (m, 1H), 5.21 (d, *J* = 12.0 Hz, 1H), 5.09 (d, *J* = 12.0 Hz, 1H), 4.95-4.84 (m, 2H), 4.23 (d, *J* = 10.6 Hz, 1H), 3.53 (d, *J* = 13.3 Hz, 1H), 2.11-1.93 (m, 2H), 1.88-1.63 (m, 9H), 1.59-1.49 (m, 2H), 1.40-1.25 (m, 3H); $^{13}C\{^1H\}$ NMR (101 MHz, $CDCl_3$) δ 157.0 (C), 137.3 (CH), 135.8 (C), 128.5 (CH × 3), 128.3 (CH × 2), 119.8 (C), 115.0 (CH_2), 67.6 (CH_2), 58.7 (C), 55.5 (CH), 46.6 (CH), 36.0 (CH_2), 31.5 (CH_2), 31.2 (CH_2), 30.5 (CH_2), 26.0 (CH_2), 25.1 (CH_2), 23.3 (CH_2 × 2). These NMR data are in agreement with those reported in the literature. [14]

***rel*-(2*R*,4a*S*,8a*R*)-*N*-Benzyloxycarbonyl-2-*n*-butyl-8a-cyanodecahydroquinoline (15):**

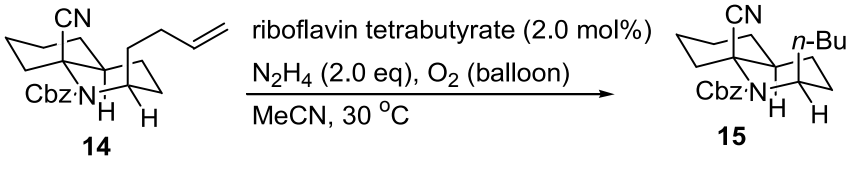

To a 50 mL flask charged with the alkene **14** (466 mg, 1.322 mmol, 1.0 eq) and riboflavin tetrabutyrate (17 mg, 26.4 μmol, 2.0 mol%) were added ACN (10 mL) and N_2H_4•H_2O (0.128 mL, 2.64 mmol, 2.0 eq). The solution was stirred under an oxygen balloon at 30 °C overnight. Evaporation of the volatile substance afforded the resulting crude reside, which was purified by flash chromatography on silica gel, EA / n-Hex as

the eluant to give white solid **15** (462 mg, 1.306 mmol, 99%), mp = 96−99 °C, (lit. [14] mp = 96−98 °C); R_f = 0.58 (EA / n-Hex = 1 : 3); ^{1}H NMR (400 MHz, CDCl$_3$) δ 7.38-7.30 (m, 5H), 5.21 (d, J = 12.0 Hz, 1H), 5.08 (d, J = 12.0 Hz, 1H), 4.19 (t, J = 5.3 Hz, 1H), 3.52 (d, J = 12.9 Hz, 1H), 1.98-1.89 (m, 1H), 1.78-1.59 (m, 8H), 1.56-1.25 (m, 5H), 1.22-1.12 (m, 3H), 1.06-1.01 (m, 1H), 0.81 (t, J = 7.0 Hz, 3H); ^{13}C{^{1}H} NMR (101 MHz, CDCl$_3$) δ 157.1 (C), 135.9 (C), 128.4 (CH × 3), 128.2 (CH × 2), 119.8 (C), 67.5 (CH$_2$), 58.6 (C), 56.2 (CH), 46.6 (CH), 36.0 (CH$_2$), 31.9 (CH$_2$), 30.5 (CH$_2$), 29.6 (CH$_2$), 26.3 (CH$_2$), 25.1 (CH$_2$), 23.4 (CH$_2$), 23.3 (CH$_2$), 22.3 (CH$_2$), 13.9 (CH$_3$). These NMR data are in agreement with those reported in the literature. [14]

***rel*-(2*R*,4a*S*,8a*R*)-*N*-Acetyl-2-*n*-butyl-8a-cyanodecahydroquinoline (3):**

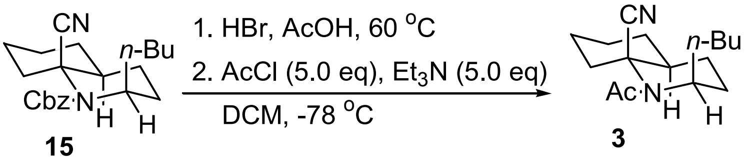

A mixed solution of carbamate **15** (462 mg, 1.303 mmol, 1.0 eq) in HBr$_{(aq)}$ (48%, 3 mL) and acetic acid (12 mL) was stirred at 60 ℃ for 14 h. Upon completion of the reaction monitored by TLC analysis, the solution was concentrated under reduced pressure to give a crude residue. The crude product was used directly without further purification.

To a CH$_2$Cl$_2$ solution (10 mL) of acetyl chloride (0.463 mL, 6.52 mmol, 5.0 eq) at -78 ℃ was slowly added Et$_3$N (0.909 mL, 6.52 mmol, 5.0 eq), and the solution was stirred for another 10 min at -78 ℃. To the freshly prepared acetylating reagent was cannulated the crude amine in CH$_2$Cl$_2$ solution (10 mL). The solution was stirred for 2 h at -78 ℃. Upon completion of the reaction monitored by TLC analysis, a saturated NaHCO$_3$ solution was slowly added. After the separation of the organic layer, the aqueous layer was extracted with CH$_2$Cl$_2$. The combined organic extracts were dried over Na$_2$SO$_4$. After the removal of the solid dehydrating agent, the organic layer was concentrated under reduced pressure to give a crude product. Purification of the crude product by flash chromatography on silica gel, EA / n-Hex as the eluant to give yellow solid **3** (243 mg, 0.926 mmol, 71%), mp = 78−80 °C, (lit. [14] mp = 78−80 °C); R_f = 0.31 (EA / n-Hex = 1 : 5); ^{1}H NMR (400 MHz, CDCl$_3$) δ 3.85 (d, J = 11.3 Hz, 1H), 3.53 (d, J = 13.3 Hz, 1H), 2.20-2.04 (m, 4H), 1.86-1.80 (m, 2H), 1.74-1.58 (m, 6H), 1.49-1.23 (m, 7H), 1.18-1.11 (m, 2H), 0.89-0.83 (m, 3H); ^{13}C{^{1}H} NMR (101 MHz, CDCl$_3$) δ 175.3 (C), 119.5 (C), 57.9 (C), 56.3 (CH), 46.5 (CH), 35.4 (CH$_2$), 32.6 (CH$_2$), 30.4 (CH$_2$), 30.0 (CH$_2$), 26.3 (CH$_2$), 25.2 (CH$_2$), 24.7 (CH$_3$), 23.3 (CH$_2$), 23.1 (CH$_2$), 22.4 (CH$_2$), 14.0 (CH$_3$). These NMR data are in agreement with those reported in the literature. [14]

***rel*-(2*R*,5*S*,10*R*)-2-Butyl-11,13-dioxodecahydro-*H*-pyrrolo[2,1-j]quinoline (16):**

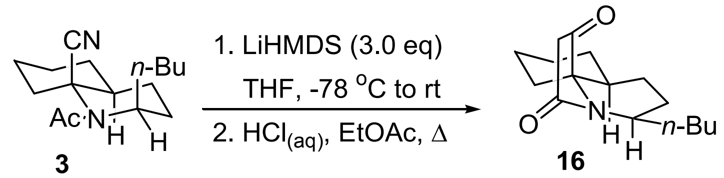

To a THF solution (10 mL) of acetamide **3** (243 mg, 0.926 mmol, 1.0 eq) at -78 °C was slowly added LHMDS solution (1.0 M in THF, 2.78 mL, 2.78 mmol, 3.0 eq) via a syringe. The reaction mixture was stirred at -78 °C for 1 h and warmed up gradually to rt in 2 h. After the slow addition of $HCl_{(aq)}$ (1.0 N, 10 mL) and EA (10 mL), the reaction was heated at reflux for 13 h. After the separation of the organic layer, the aqueous layer was extracted with EA. The combined organic layers were dried over Na_2SO_4. After the removal of the solid dehydrating agent, the organic layer was concentrated under reduced pressure to give a crude product. Purification of the crude product by flash chromatography on silica gel, EA / n-Hex as the eluant to give yellow oil **16** (188 mg, 0.714 mmol, 77%). R_f = 0.5 (EA / n-Hex = 1 : 4); ^{1}H NMR (400 MHz, $CDCl_3$) δ 3.29-3.19 (m, 2H), 2.74-2.64 (m, 1H), 2.57-2.49 (m, 1H), 2.30-2.18 (m, 1H), 1.88-1.64 (m, 9H), 1.53-1.23 (m, 7H), 1.18-1.06 (m, 1H), 0.90 (t, J = 7.0 Hz, 3H); $^{13}C\{^1H\}$ NMR (101 MHz, $CDCl_3$) δ 209.0 (C), 170.2 (C), 73.7 (C), 52.8 (CH), 43.0 (CH_2), 42.4 (CH), 33.9 (CH_2), 32.5 (CH_2), 30.0 (CH_2), 29.9 (CH_2), 26.3 (CH_2), 25.9 (CH_2), 24.5 (CH_2), 22.5 (CH_2), 21.2 (CH_2), 14.0 (CH_3). These NMR data are in agreement with those reported in the literature. [14]

***rel*-(2*R*,5*S*,10*R*)-2-Butyl-11,12-didehydro-13-oxodecahydro-*H*-pyrrolo[2,1-j]quinoline (17):**

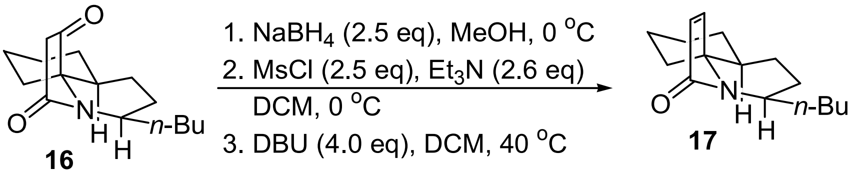

To a MeOH solution (10 mL) of ketone **16** (188 mg, 0.714 mmol, 1.0 eq) at 0 °C was added $NaBH_4$ (68 mg, 1.785 mmol, 2.5 eq) in portions. The reaction mixture was stirred in the ice bath for 1 h. Upon completion of the reaction monitored by TLC analysis, and quenched with saturated NH_4Cl solution. After evaporation of the volatile substance, the resulting solution was partitioned with CH_2Cl_2. The combined organic extracts were dried over Na_2SO_4. After the removal of the solid dehydrating agent, the organic solution was concentrated under reduced pressure to give a crude product. The crude alcohol product was used directly without further purification.

To a CH_2Cl_2 solution (10 mL) of the crude alcohol, cooled in an ice bath, was added Et_3N (0.259 mL, 1.856 mmol, 2.6 eq) via a syringe followed by MsCl (0.138

mL, 1.785 mmol, 2.5 eq). The reaction was stirred in the ice bath for 1 h. Upon completion of the reaction monitored by TLC analysis, a saturated $NaHCO_3$ solution was slowly added. After separation of the organic layer, the aqueous layer was extracted with CH_2Cl_2. The combined organic extracts were dried over Na_2SO_4. After the removal of the solid dehydrating agent, the extracts were concentrated under reduced pressure to give a crude product. The crude product was used directly without further purification.

The crude mesylate was diluted with CH_2Cl_2 (10 mL) followed by the addition of DBU (0.427 mL, 2.86 mmol, 4.0 eq). The reaction mixture was stirred and heated at reflux for 16 h. Upon completion of the reaction monitored by TLC analysis, the reaction was quenched with saturated NH_4Cl solution. After separation from the organic extracts, the aqueous solution was extracted with CH_2Cl_2. The combined organic extracts were dried over Na_2SO_4. After the removal of the solid dehydrating agent, the organic layer was concentrated under reduced pressure to give a crude product. Purification of the crude product by flash chromatography on silica gel, EA / n-Hex as the eluant to give yellow oil **17** (153 mg, 0.618 mmol, 87%): R_f = 0.48 (EA / n-Hex = 1 ∶ 2); ^1H NMR (400 MHz, $CDCl_3$) δ 7.24 (s, 1H), 6.06 (d, J = 5.9 Hz, 1H), 3.25-3.17 (m, 1H), 2.52-2.43 (m, 1H), 1.90-1.78 (m, 6H), 1.72-1.58 (m, 4H), 1.40-1.22 (m, 8H), 0.89-0.76 (m, 3H); $^{13}C\{^1H\}$ NMR (101 MHz, $CDCl_3$) δ 176.6 (C), 149.3 (CH), 129.1 (CH), 72.4 (C), 52.2 (CH), 43.7 (CH), 35.6 (CH_2), 32.0 (CH_2), 31.8 (CH_2), 29.7 (CH_2), 29.5 (CH_2), 26.7 (CH_2), 23.8 (CH_2), 22.6 (CH_2), 22.5 (CH_2), 14.1 (CH_3). These NMR data are in agreement with those reported in the literature. [14]

***rel*-(2*R*,5*S*,10*S*)-2-Butyl-13-oxodecahydro-*H*-pyrrolo[2,1-j]quinoline (2):**

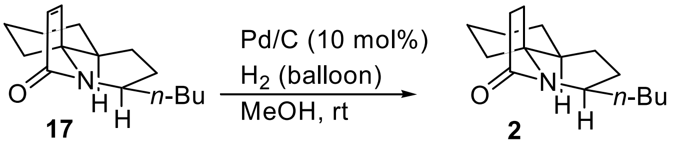

To a 100 mL flask containing a freshly activated Pd/C catalyst (10%, 66 mg, 0.0618 mmol, 10 mol%) was added MeOH solution (20 mL) of **17** (153 mg, 0.618 mmol, 1.0 eq). The reaction suspension was stirred overnight under a hydrogen balloon. Upon completion of the reaction monitored by TLC analysis, the suspension was filtered through celite and the filtrate was concentrated under reduced pressure to give a crude product. Purification of the crude product by flash chromatography on silica gel, EA / n-Hex as the eluant to give colorless oil **2** (139 mg, 0.557 mmol, 90%): R_f = 0.38 (EA / n-Hex = 1 ∶ 2); ^1H NMR (400 MHz, $CDCl_3$) δ 3.16 (t, J = 7.4 Hz, 1H), 2.49-2.40 (m, 2H), 2.11 (dd, J = 16.0, 9.0 Hz, 1H), 1.87 (dd, J = 12.3, 8.4 Hz, 1H), 1.80-1.54 (m, 8H), 1.53-1.40 (m, 3H), 1.38-1.24 (m, 7H), 1.22-1.14 (m, 1H), 0.89 (t, J = 6.8 Hz, 3H); $^{13}C\{^1H\}$ NMR (101 MHz, $CDCl_3$) δ 176.3 (C), 66.2 (C), 51.7 (CH), 42.5 (CH), 33.2

(CH_2), 31.6 (CH_2 × 2), 30.4 (CH_2), 30.0 (CH_2), 27.2 (CH_2), 26.1 (CH_2), 24.4 (CH_2), 23.5 (CH_2), 22.5 (CH_2), 22.1 (CH_2), 14.1 (CH_3). The NMR data are in agreement with those reported in the literature. [14]

***rel*-(2*R*,5*S*,10*S*)-2-Butyl-13-oxodecahydro-*H*-pyrrolo[2,1-j]quinoline (Lepadiformine C, 1c):**

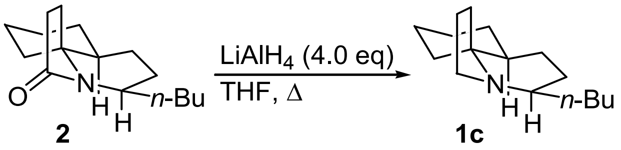

To a THF (10 mL) solution of lactam **2** (56 mg, 0.225 mmol, 1.0 eq) in an ice bath under argon was slowly added $LiAlH_4$ (34 mg, 0.900 mmol, 4.0 eq) in portions. The reaction mixture was stirred under reflux conditions overnight. Upon completion of the reaction monitored by TLC analysis, the reaction mixture was cooled down in an ice bath. The addition of water (0.1 mL), $NaOH_{(aq)}$ (15%, 0.1 mL), and water (0.2 mL) in sequence resulted in the formation of white solid precipitates, which were filtered off by a short celite pad to give a filtrate. The filtrate was concentrated under reduced pressure to give a crude product. The crude product was purified by a short chromatography on silica gel, CH_2Cl_2 / MeOH / $NH_{3(aq)}$ (28%) as the eluant to give yellow oil **1c** (40 mg, 0.170 mmol, 76%): R_f = 0.16 ($CHCl_3$ / MeOH = 1 : 9); ^1H NMR (400 MHz, $CDCl_3$) δ 3.16 (br s, 1H), 2.98 (br s, 1H), 2.73-2.65 (m, 1H), 1.82-1.48 (m, 10H), 1.48-1.17 (m, 12H), 1.09-0.99 (m, 1H), 0.89 (t, J = 7.4 Hz, 3H); $^{13}C\{^1H\}$ NMR (101 MHz, $CDCl_3$) δ 66.4 (C), 51.8 (CH), 46.9 (CH_2), 39.8 (CH), 38.9 (CH_2), 34.1 (CH_2), 29.9 (CH_2), 29.1 (CH_2), 28.6 (CH_2), 26.3 (CH_2), 24.5 (CH_2), 23.2 (CH_2), 22.8 (CH_2), 22.4 (CH_2), 22.3 (CH_2), 14.0 (CH_3); IR (cm^{-1}, film) ν_{max} = 2930, 2860, 1450, 1275; EI-HRMS (m/z) : $[M]^+$ calcd for $C_{16}H_{29}N^+$ 235.2300, found 235.2303 (Δ = 1.3 ppm). The NMR data are in agreement with those reported in the literature. [13]

General Procedure for the Ir-catalyzed reductive cyanation and Subsequent Brulyants Transformations or Methanolysis:

***rel*-(2*R*,5*S*,10*S*,13*S*)-2-Butyl-13-cyanodecahydro-*H*-pyrrolo[2,1-j]qui-noline (18):**

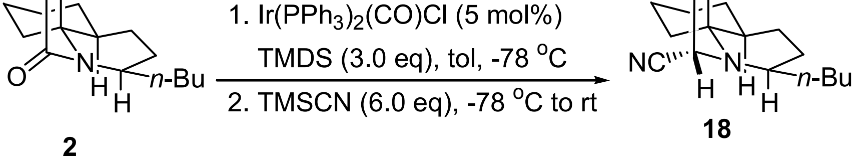

To a toluene solution (10 mL) of lactam **2** (216 mg, 0.866 mmol, 1.0 eq) was added $Ir(PPh_3)_2(CO)Cl$ (34 mg, 0.0433 mmol, 5.0 mol%). The reaction mixture was stirred 10 minutes at -78 °C, then TMDS (0.459 mL, 2.60 mmol, 3.0 eq) was added in one portion.

Upon completion of the reaction monitored by TLC analysis, TMSCN (0.650 mL, 5.20 mmol, 6.0 eq) was added and stirred at room temperature overnight. The solution was quenched with saturated $NaHCO_3$ solution. After separation of the organic layer, the aqueous layer was extracted with EA. The combined organic layers were dried over Na_2SO_4. After removal of the solid dehydrating agent, the organic layer was concentrated under reduced pressure to give a crude product. The crude product **18** was used directly without further purification due to its liability.

***rel*-(2*R*,5*S*,10*S*,13*S*)-2-Butyl-13-ethynyl-decahydro-*H*-pyrrolo[2,1-j]quinoline (19):**

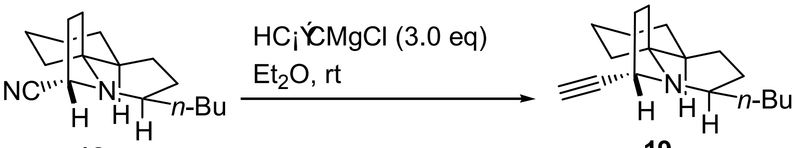

18 **19**

The crude aminonitrile product **18** (from 101 mg of **2**, 0.405 mmol) was dissolved in dry Et_2O (10 mL) and added dropwise to a solution of ethynylmagnesium chloride (0.5 M in THF, 2.43 mL, 1.215 mmol, 3.0 eq) at 0 ℃. Upon completion of the reaction monitored by TLC analysis, the reaction mixture was added 6 N $NaOH_{(aq)}$ in sequence resulted in the formation of white solid precipitates, which were filtered off by a short celite pad to give a filtrate. The filtrate was separation of the organic layer, the aqueous layer was extracted with Et_2O. The combined organic layers were dried over Na_2SO_4. After the removal of the solid dehydrating agent, the organic layer was concentrated under reduced pressure to give a crude product. Purification of the crude product by flash chromatography on silica gel, CH_2Cl_2 / MeOH / $NH_{3(aq)}$ (28%) as the eluant to give colorless oil **19** (36 mg, 0.139 mmol, 34%): R_f = 0.28 (MeOH / $CHCl_3$ = 1 ∶ 9); ^{1}H NMR (400 MHz, $CDCl_3$) δ 3.54-3.50 (m, 1H), 3.12-3.09 (m, 1H), 2.31-2.26 (m, 1H), 1.72–1.62 (m, 10H), 1.38–1.25 (m, 11H), 1.14-1.06 (m, 2H), 0.87 (t, *J* = 7.1 Hz, 3H); $^{13}C\{^1H\}$ NMR (101 MHz, $CDCl_3$) δ 84.2 (C), 72.9 (CH), 67.7 (C), 61.2 (CH), 53.1 (CH), 40.1 (CH), 38.8 (CH_2), 33.8 (CH_2), 30.5 (CH_2), 29.9 (CH_2), 28.1 (CH_2), 27.7 (CH_2), 26.3 (CH_2), 24.5 (CH_2), 23.5 (CH_2), 22.8 (CH_2), 22.6 (CH_2), 14.3 (CH_3); IR (cm^{-1}, film) ν_{max} = 3386, 2927, 2857, 2150, 1460, 1077, 1039; EI-HRMS (m/z) ∶ $[M]^+$ calcd for $C_{18}H_{29}N^+$ 259.2300, found 259.2305 (Δ = 1.9 ppm).

***rel*-(2*R*,5*S*,10*S*,13*S*)-2-Butyl-13-trimethylsilylmethyl-decahydro-*H*-pyrrolo[2,1-j]-quinoline (20):**

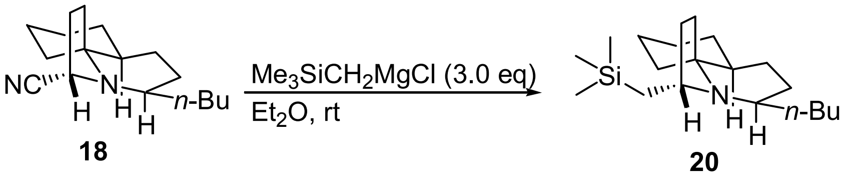

The crude aminonitrile product **18** (from 101 mg of **2**, 0.405 mmol) was dissolved in dry Et_2O (10 mL) and added dropwise to a solution of (Trimethylsilyl)methylmagnesium chloride solution (1.0 M in Et_2O, 1.215 mL, 1.215 mmol, 3.0 eq) at 0 °C. Upon completion of the reaction monitored by TLC analysis, the reaction mixture was added 6 N $NaOH_{(aq)}$ in sequence resulted in the formation of white solid precipitates, which were filtered off by a short celite pad to give a filtrate. The filtrate was separation of the organic layer, the aqueous layer was extracted with Et_2O. The combined organic layers were dried over Na_2SO_4. After the removal of the solid dehydrating agent, the organic layer was concentrated under reduced pressure to give a crude product. Purification of the crude product by flash chromatography on silica gel, CH_2Cl_2 / MeOH / $NH_{3(aq)}$ (28%) as the eluant to give colorless oil **20** (74 mg, 0.230 mmol, 57%): R_f = 0.31 (MeOH / $CHCl_3$ = 1 ∶ 9); ^{1}H NMR (400 MHz, $CDCl_3$) δ 3.54-3.50 (m, 2H), 3.34-3.30 (m, 1H), 3.12-3.09 (m, 1H), 1.72–1.62 (m, 11H), 1.38–1.25 (m, 10H), 1.14-1.06 (m, 2H), 0.87 (t, J = 7.1 Hz, 3H), 0.06 (s, 9H); $^{13}C\{^1H\}$ NMR (101 MHz, $CDCl_3$) δ 67.7 (C), 62.2 (CH), 56.0 (CH_2), 53.1 (CH), 40.1 (CH), 38.8 (CH_2), 33.8 (CH_2), 30.5 (CH_2), 29.9 (CH_2), 28.1 (CH_2), 27.7 (CH_2), 26.3 (CH_2), 24.5 (CH_2), 23.5 (CH_2), 22.8 (CH_2), 22.6 (CH_2), 14.3 (CH_3), 0.98 (CH_3 × 3); IR (cm^{-1}, film) ν_{max} = 2927, 2857, 1460, 1077, 1039; EI-HRMS (m/z) ∶ [M]+ calcd for $C_{20}H_{39}NSi^+$ 321.2852, found 321.2855 (Δ = 0.9 ppm).

***rel*-(2*R*,5*S*,10*S*,13*S*)-2-Butyl-13-methoxycarbonyl-decahydro-*H*-pyrrolo[2,1-j]qui-noline (21):**

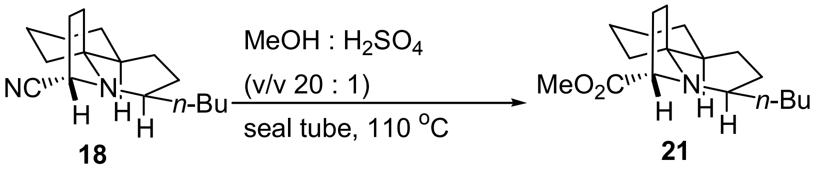

A solution (0.17 M) of the crude product **18** (from 216 mg of **2**, 0.866 mmol) in MeOH / H_2SO_4 (20:1) was heated to 110 °C in a sealed tube. After 3 days, the solution was cooled to 0 °C and adjusted to pH 8 with saturated aq. $NaHCO_3$. The aqueous layer was extracted with CH_2Cl_2. The combined organic layers were dried over Na_2SO_4. After removal of the solid dehydrating agent, the organic layer was concentrated under reduced pressure to give a crude product. Purification of the crude product by flash

chromatography on silica gel, EA / n-Hex as the eluant to give colorless oil **21** (84 mg, 0.286 mmol, 33%): R_f = 0.24 (EA); ^{1}H NMR (400 MHz, $CDCl_3$) δ 3.74-3.66 (m, 3H), 3.21-3.13 (m, 1H), 2.10-2.00 (m, 1H), 1.82-1.76 (m, 1H), 1.80-1.70 (m, 2H), 1.70-1.60 (m, 4H), 1.60-1.51 (m, 2H), 1.51-1.38 (m, 3H), 1.38-1.14 (m, 9H), 1.14-1.04 (m, 1H), 1.00-0.92 (m, 1H), 0.85 (t, J = 7.0 Hz, 3H); $^{13}C\{^1H\}$ NMR (101 MHz, $CDCl_3$) δ 177.9 (C), 67.7 (C), 61.6 (CH), 53.0 (CH), 52.0 (CH_3), 39.8 (CH), 38.6 (CH_2), 33.7 (CH_2), 30.6 (CH_2), 29.6 (CH_2), 29.4 (CH_2), 28.2 (CH_2), 26.3 (CH_2), 24.6 (CH_2), 23.0 (CH_2), 23.0 (CH_2), 22.1 (CH_2), 14.1 (CH_3); IR (cm^{-1}, film) ν_{max} = 2931, 2862, 1732; EI-HRMS (m/z) ∶ [M]$^+$ calcd for $C_{18}H_{31}NO_2^+$ 293.2355, found 293.2358 (Δ = 1.0 ppm). The NMR data are in agreement with those reported in the literature. [12]

***rel*-(2*R*,5*S*,10*S*,13*S*)-2-Butyl-13-hydroxymethyl-decahydro-*H*-pyrrolo[2,1-j]quinoline (Lepadiformine B, 1b):**

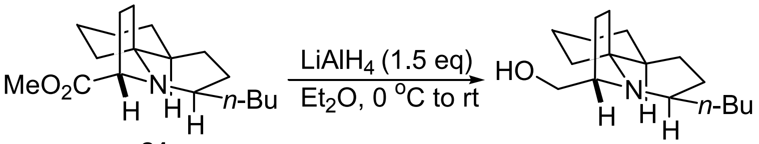

21 **1b**

To an ether solution (10 mL) of **21** (66 mg, 0.225 mmol, 1.0 eq), cooled in an ice bath under argon, was slowly added LAH (13 mg, 0.338 mmol, 1.5 eq) in portions. The cooling bath was removed and the reaction mixture was stirred at room temperature for 1 h. Upon completion of the reaction monitored by TLC analysis, the reaction mixture was added water (0.1 mL), $NaOH_{(aq)}$ (15%, 0.1 mL), and water (0.2 mL) in sequence resulted in the formation of white solid precipitates, which were filtered off by a short celite pad to give a filtrate. The filtrate was concentrated under reduced pressure to give a crude product. The crude product was purified by a short chromatography on silica gel, CH_2Cl_2 / MeOH / $NH_{3(aq)}$ (28%) as the eluant to give colorless oil **1b** (54 mg, 0.203 mmol, 90%): R_f = 0.19 (MeOH / $CHCl_3$ = 1 ∶ 9); ^{1}H NMR (400 MHz, $CDCl_3$) δ 3.54-3.50 (m, 1H), 3.34-3.30 (m, 1H), 3.12-3.09 (m, 1H), 1.72-1.62 (m, 14H), 1.38-1.25 (m, 9H), 1.14-1.06 (m, 2H), 0.87 (t, J = 7.1 Hz, 3H); $^{13}C\{^1H\}$ NMR (101 MHz, $CDCl_3$) δ 67.3 (C), 62.2 (CH), 58.3 (CH_2), 53.1 (CH), 40.1 (CH), 38.2 (CH_2), 33.8 (CH_2), 30.5 (CH_2), 29.9 (CH_2), 28.1 (CH_2), 27.7 (CH_2), 26.3 (CH_2), 24.3 (CH_2), 23.3 (CH_2), 22.8 (CH_2), 22.6 (CH_2), 14.2 (CH_3); IR (cm^{-1}, film) ν_{max} = 3386, 2927, 2857, 1460, 1077, 1039; EI-HRMS (m/z) ∶ [M]$^+$ calcd for $C_{17}H_{31}NO^+$ 265.2406, found 265.0405 (Δ = -0.4 ppm). The NMR data are in agreement with those reported in the literature. [13]

Lepadiformine B (**1b**), ^{1}H-NMR (400MHz, $CDCl_3$)

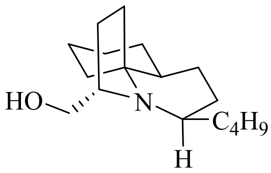

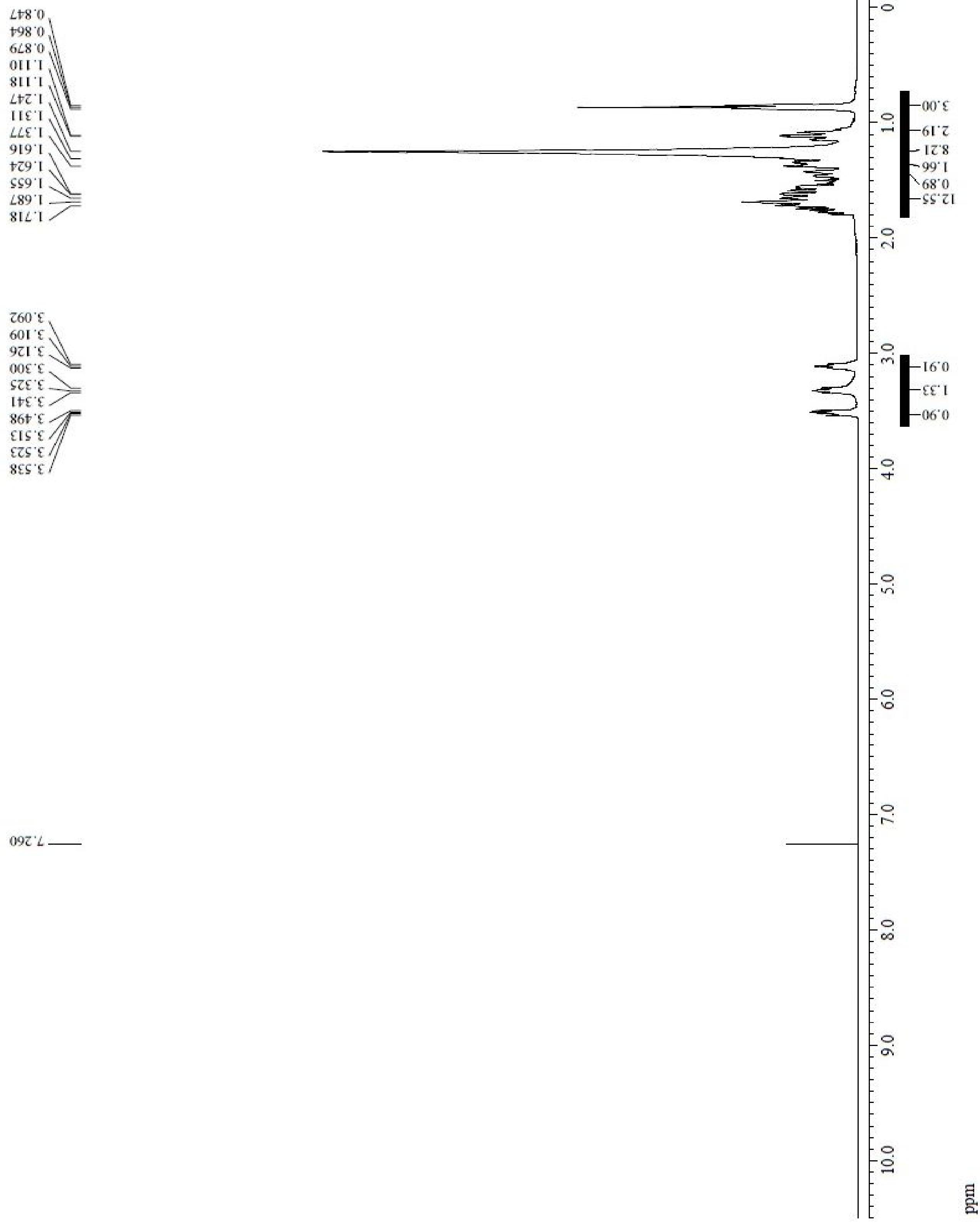

Lepadiformine B (**1b**), $^{13}C\{^{1}H\}$ NMR (101MHz, $CDCl_3$)

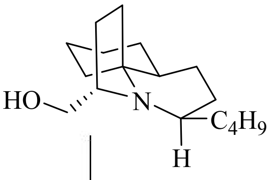

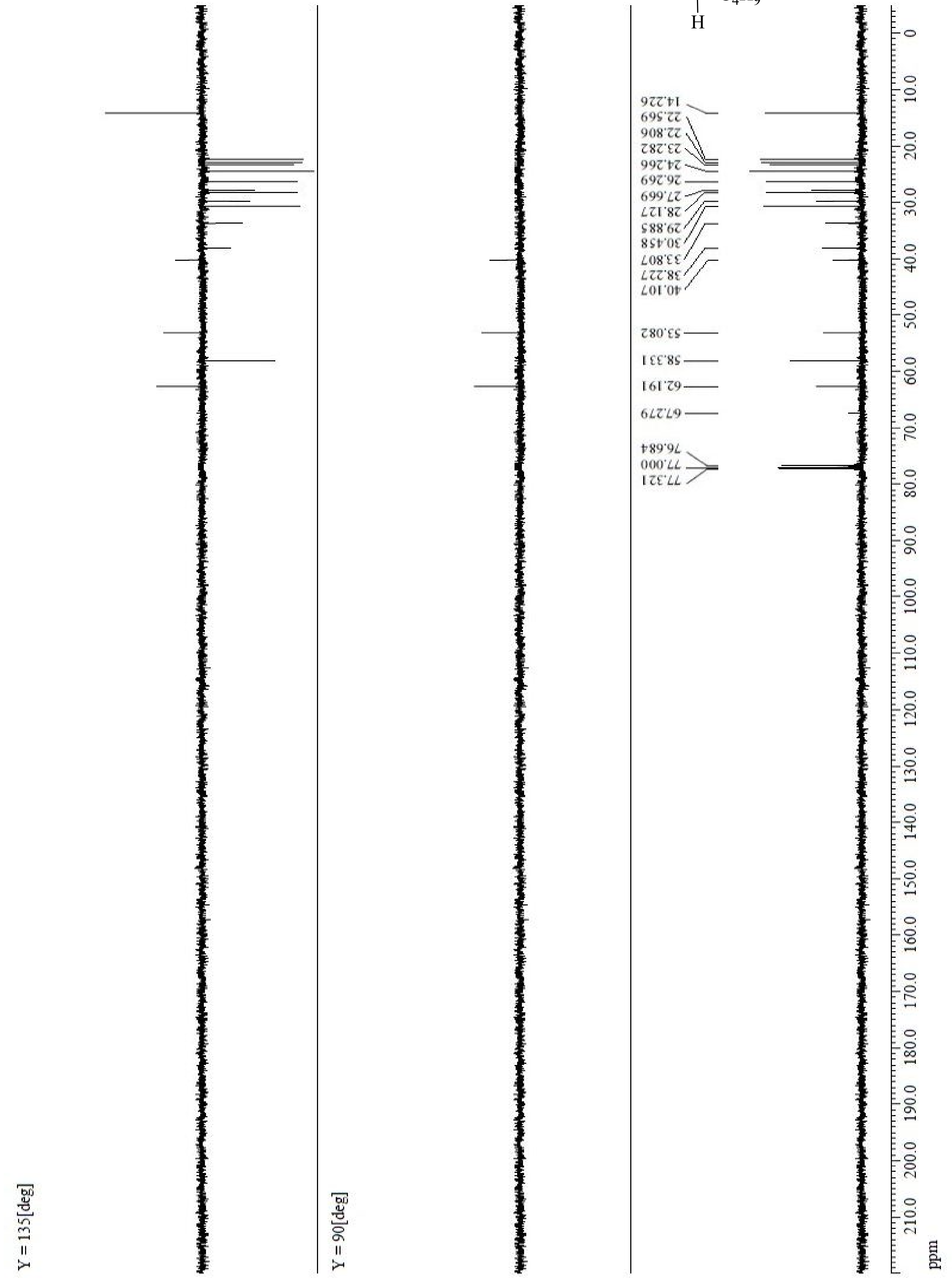

Lepadiformine C (**1c**), ^{1}H-NMR (400MHz, $CDCl_3$)

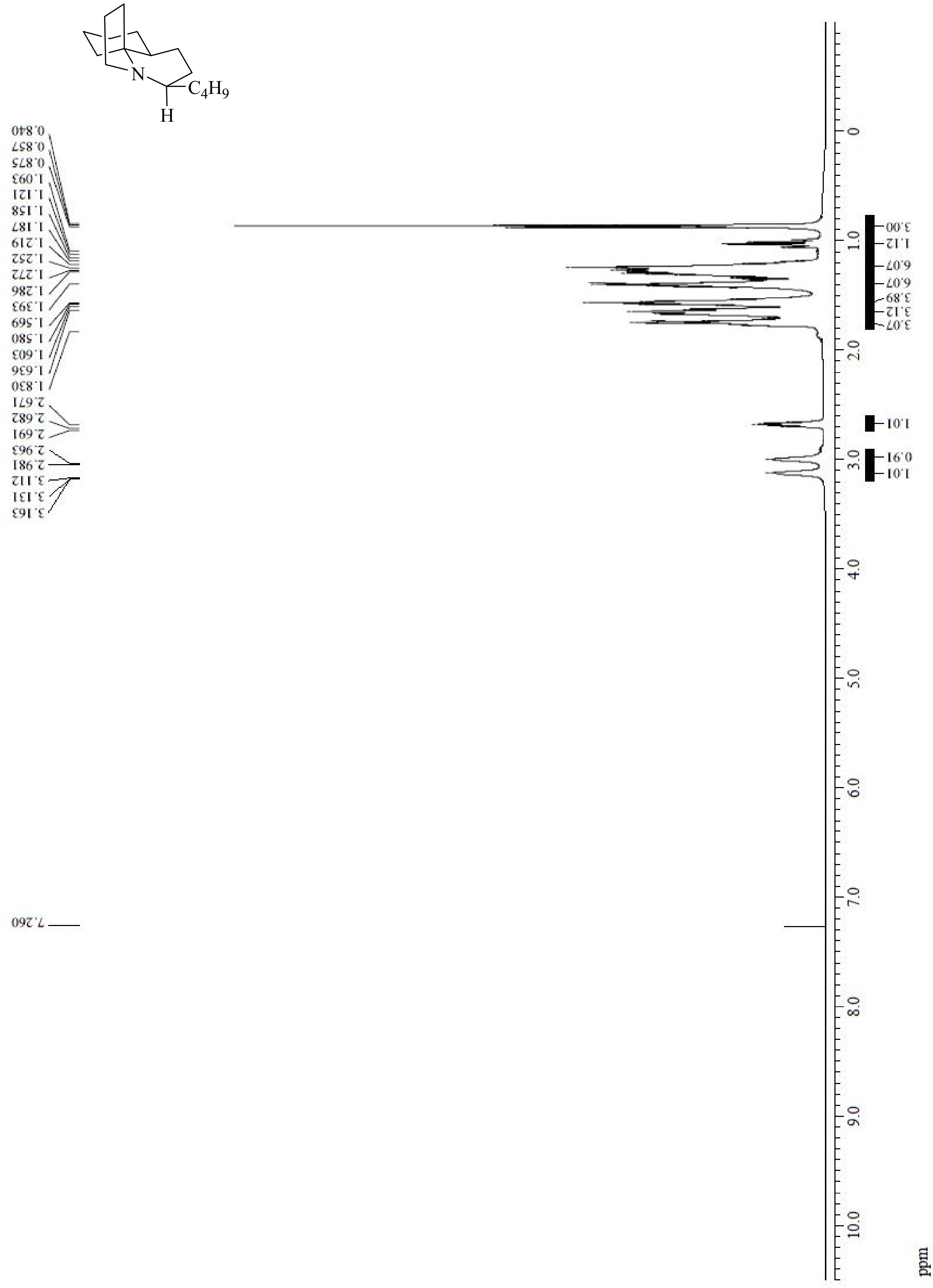

Lepadiformine C (**1c**), $^{13}C\{^{1}H\}$ NMR (101MHz, $CDCl_3$)

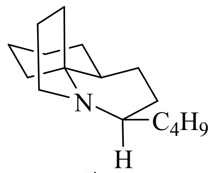

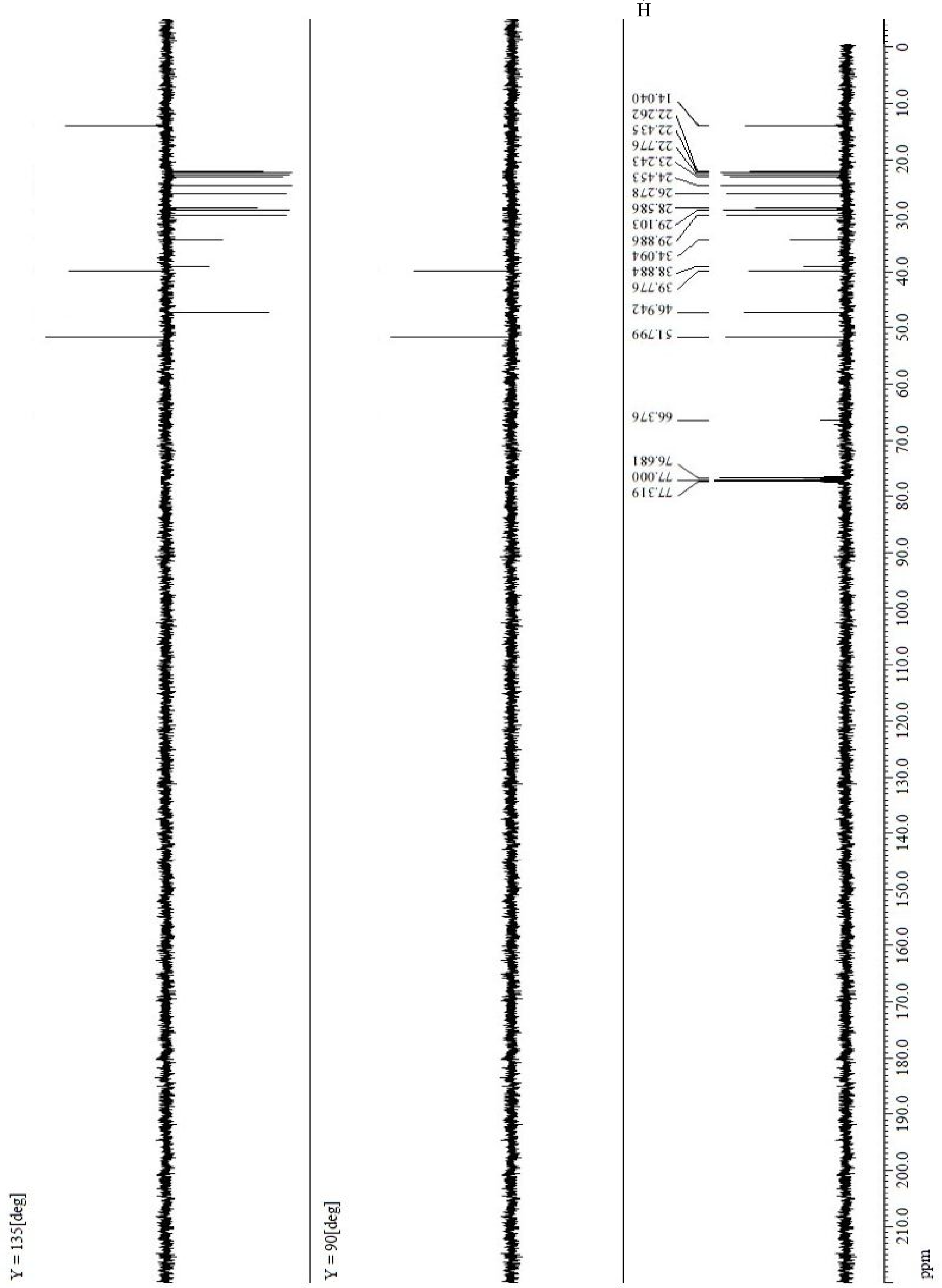

2, ^{1}H-NMR (400MHz, $CDCl_3$)

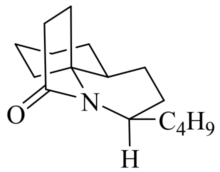

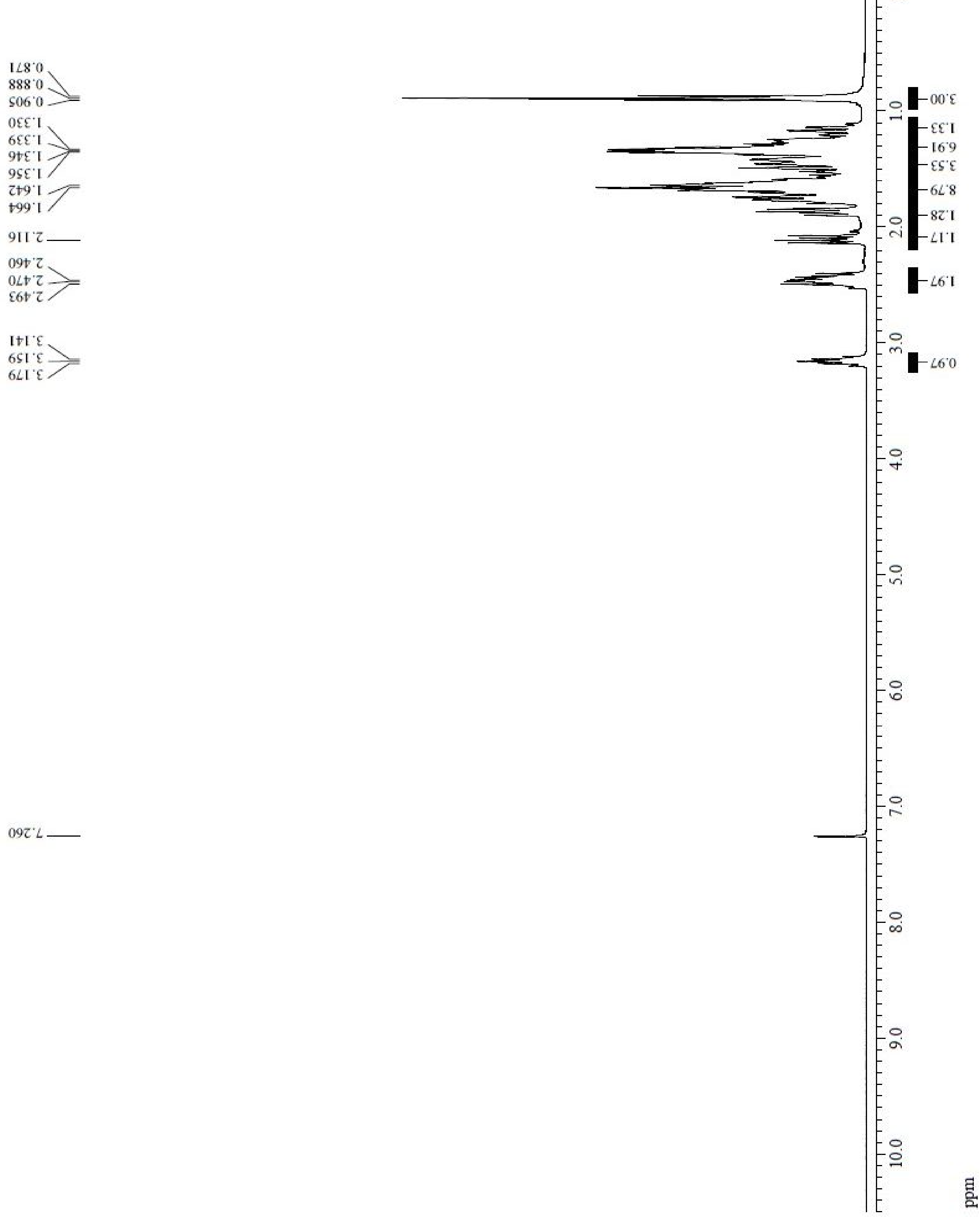

2, $^{13}C\{^{1}H\}$ NMR (101MHz, $CDCl_3$)

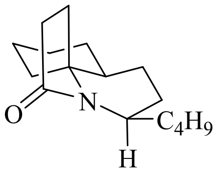

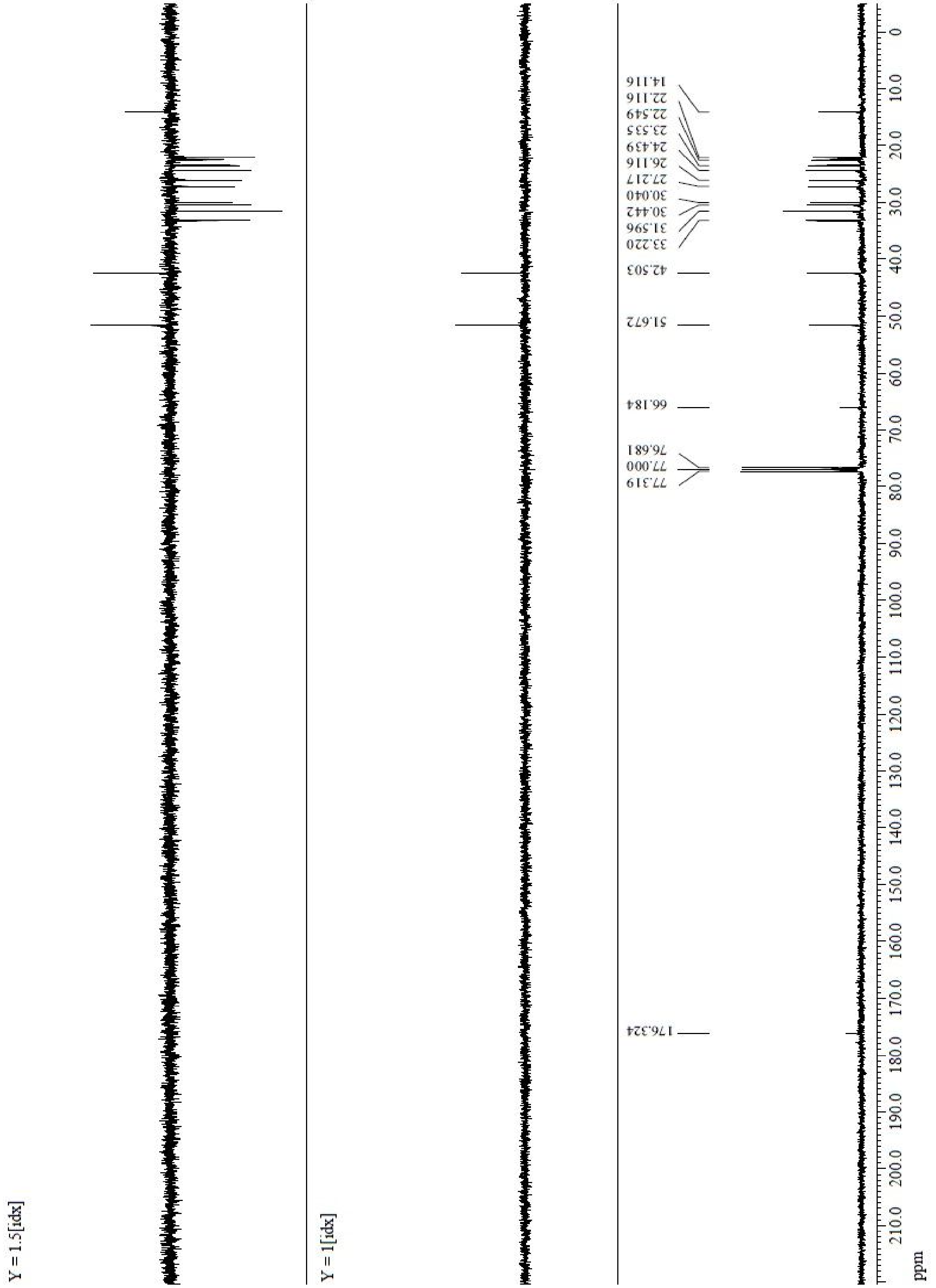

3, ^{1}H-NMR (400MHz, $CDCl_3$)

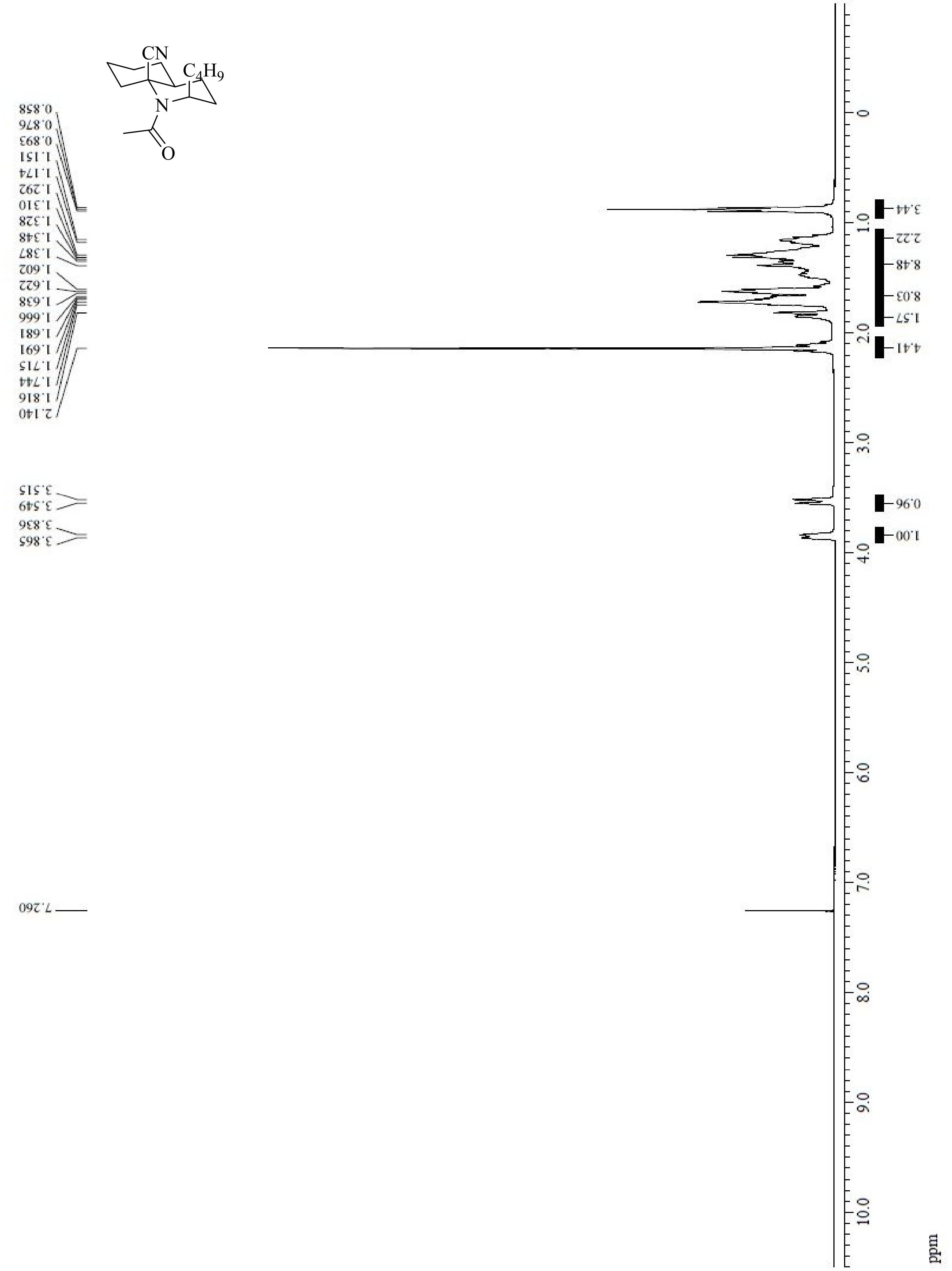

3, $^{13}C\{^{1}H\}$ NMR (101MHz, $CDCl_3$)

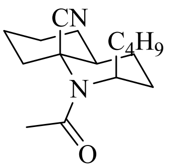

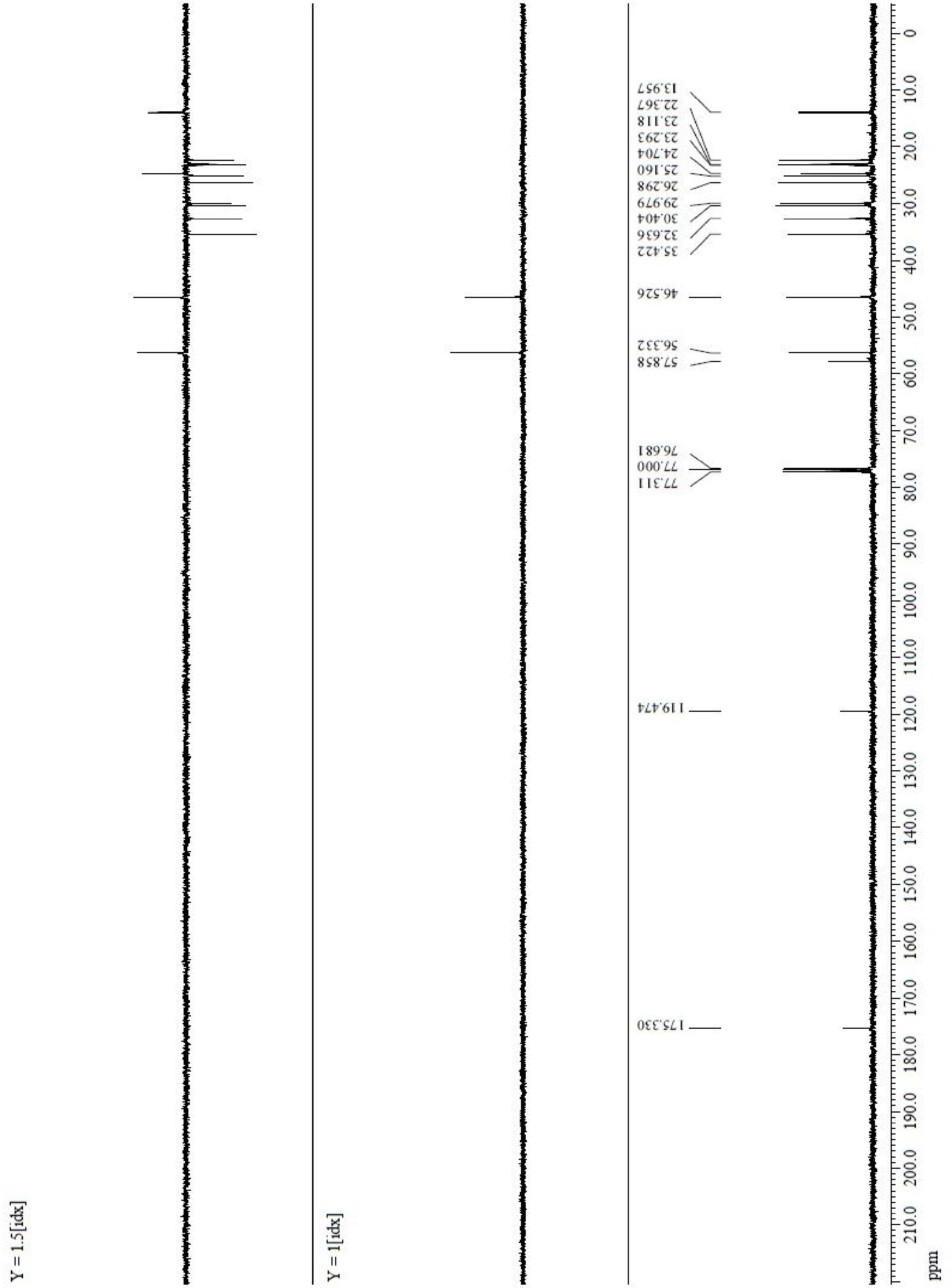

4, ^{1}H-NMR (400MHz, $CDCl_3$)

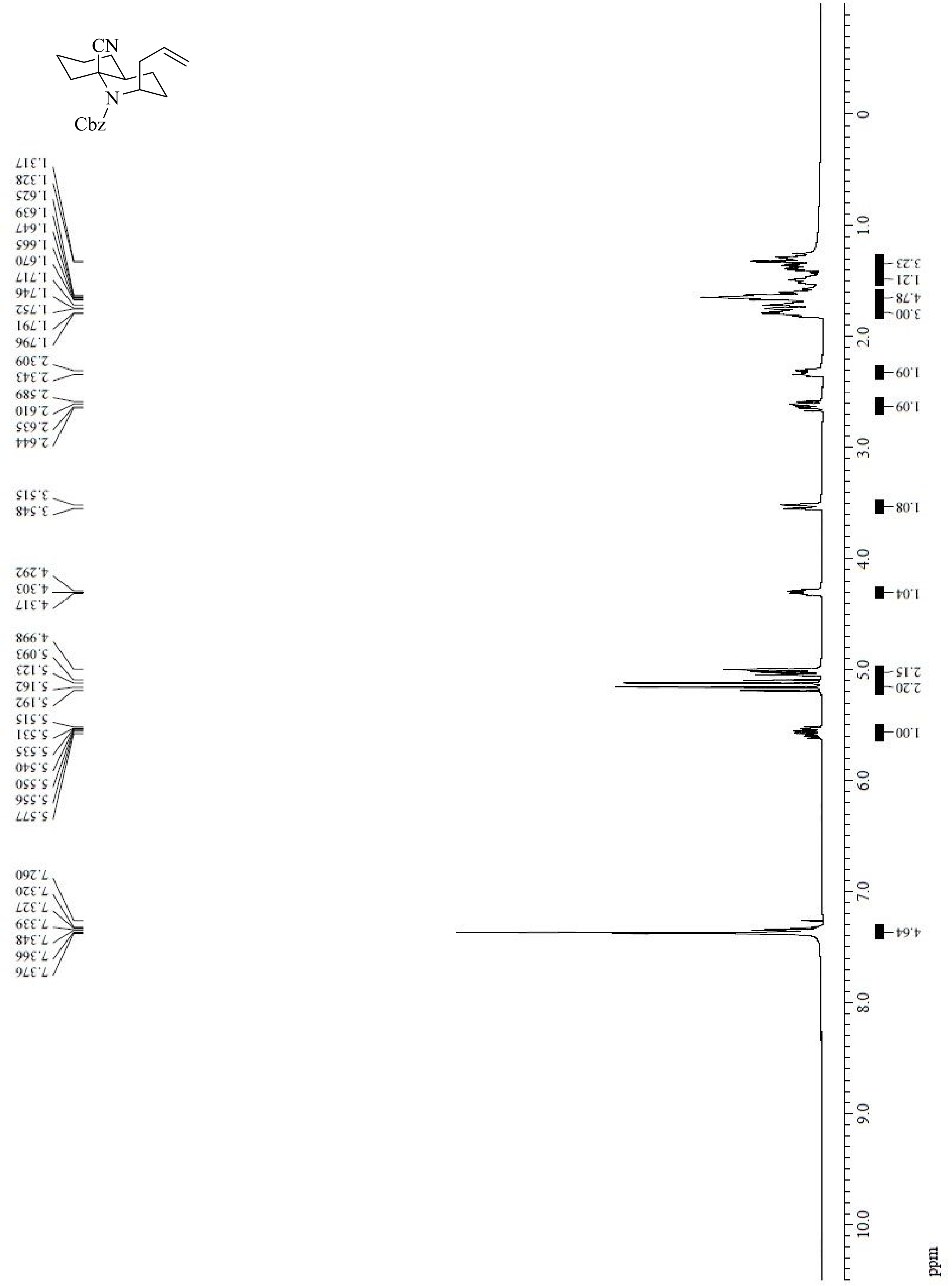

4, $^{13}C\{^{1}H\}$ NMR (101MHz, $CDCl_3$)

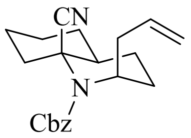

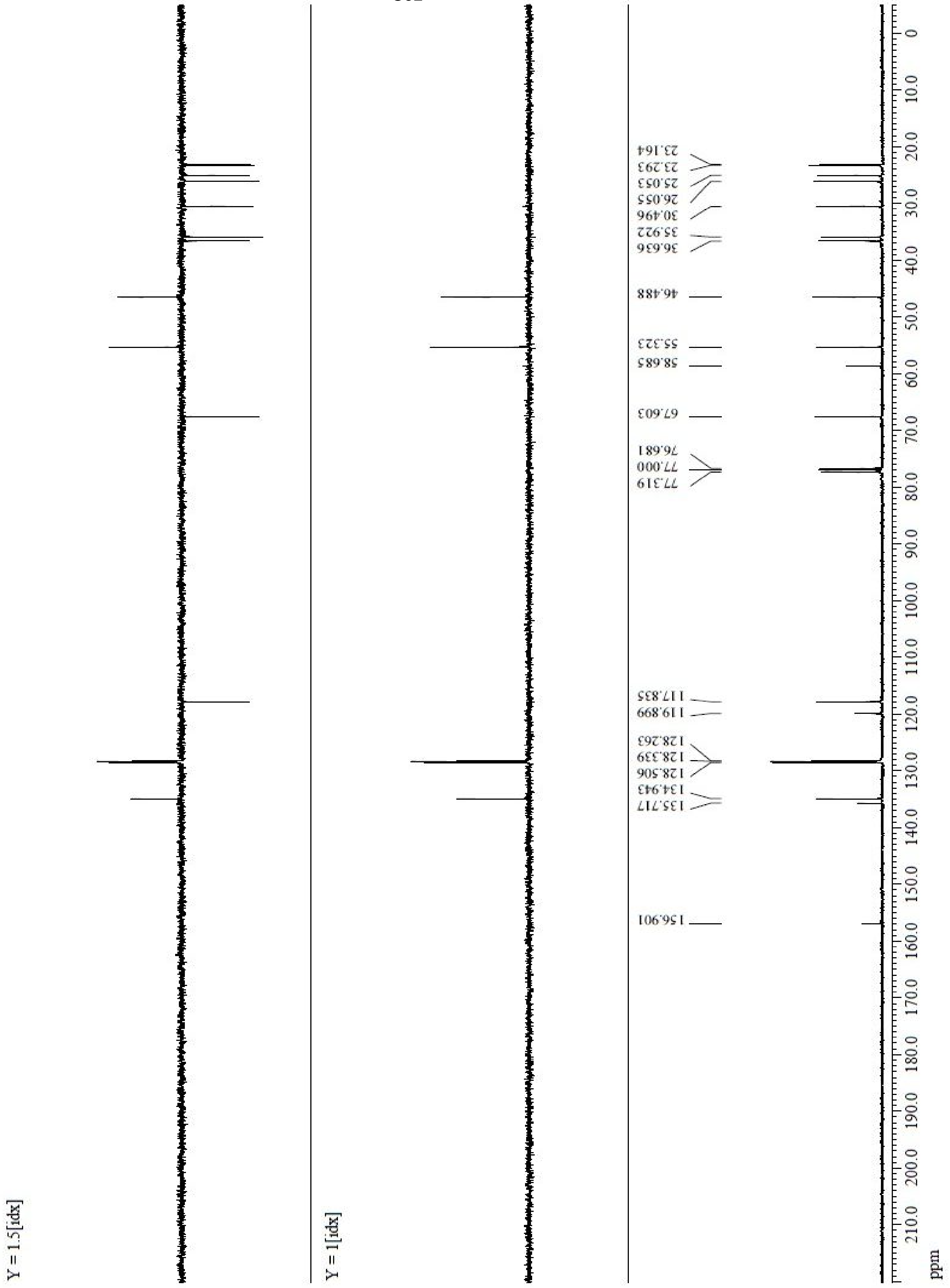

5a, ^{1}H-NMR (400MHz, $CDCl_3$)

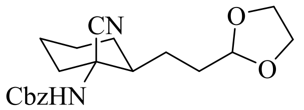

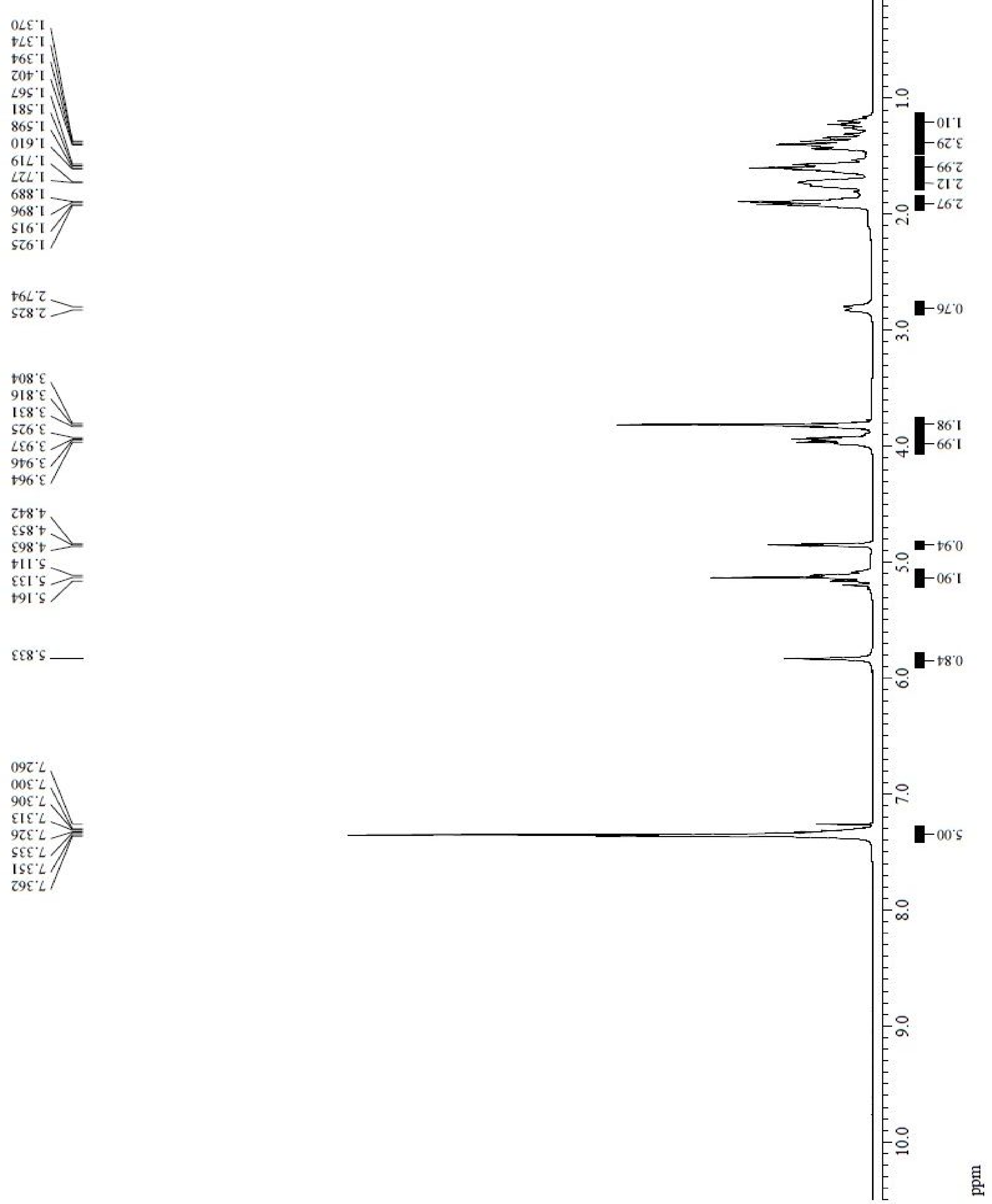

5a, $^{13}C\{^{1}H\}$ NMR (101MHz, $CDCl_3$)

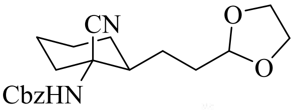

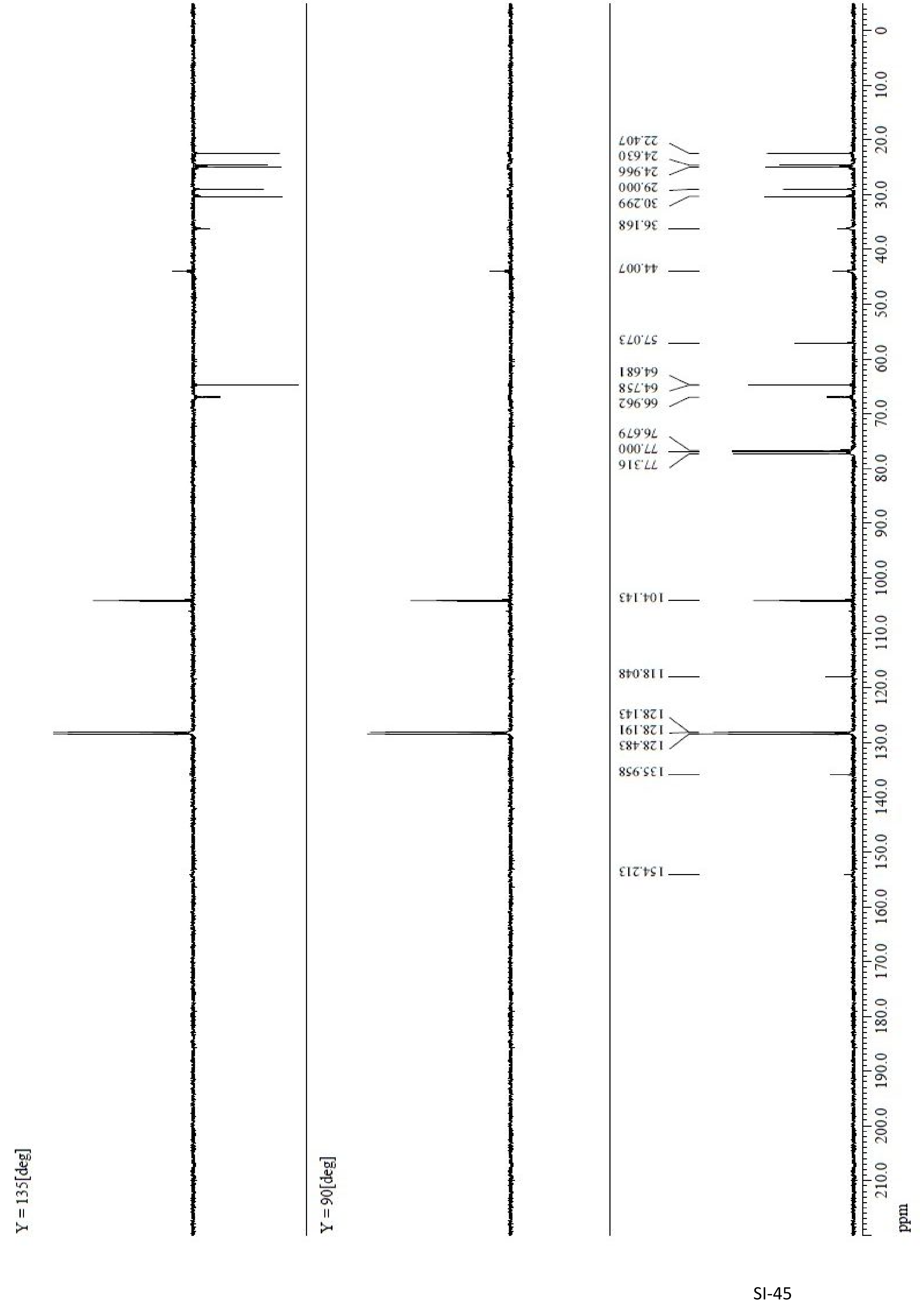

5b, ^{1}H-NMR (400MHz, $CDCl_3$)

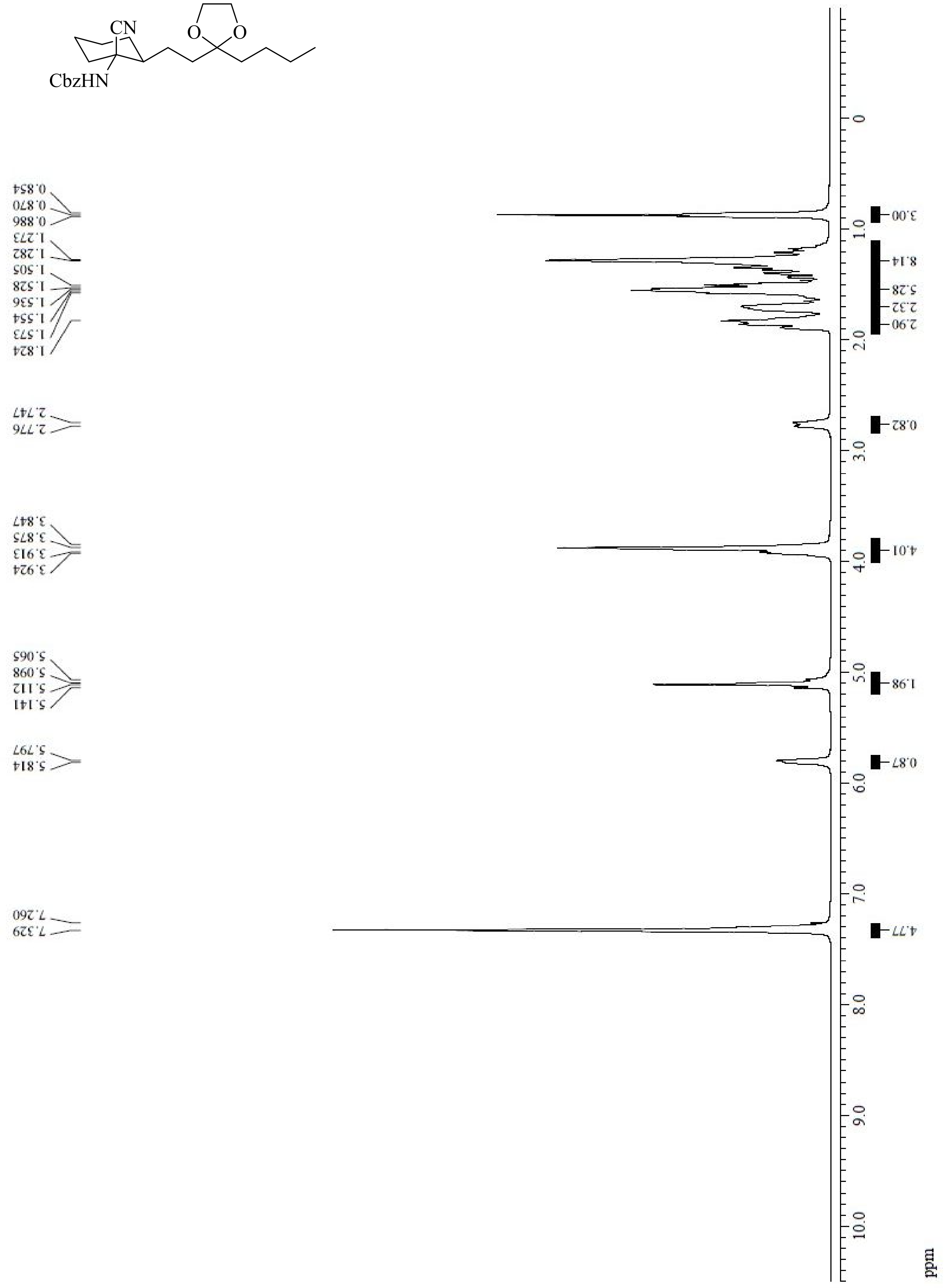

5b, $^{13}C\{^{1}H\}$ NMR (101MHz, $CDCl_3$)

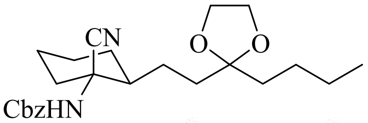

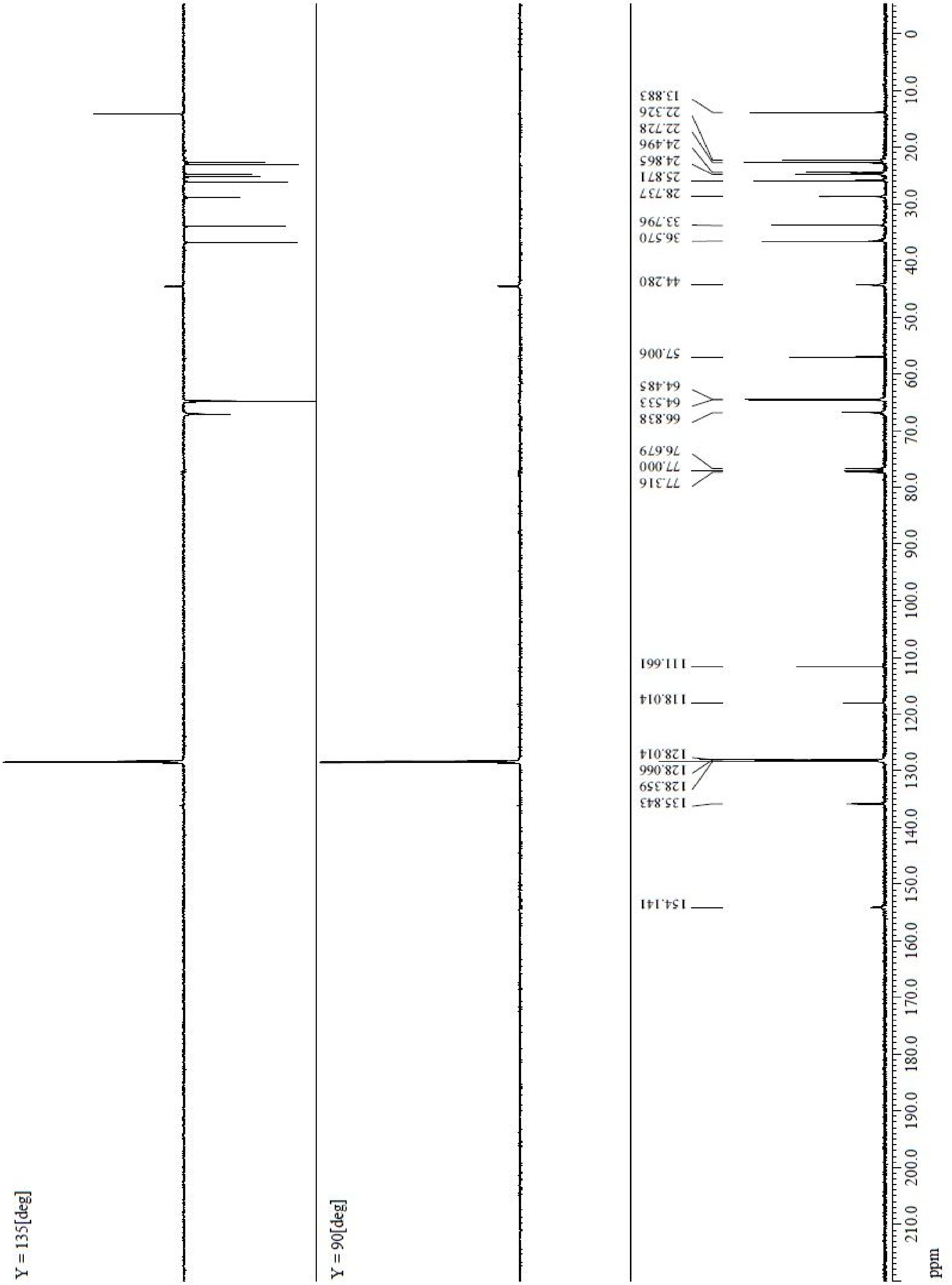

6a, ^{1}H-NMR (400MHz, $CDCl_3$)

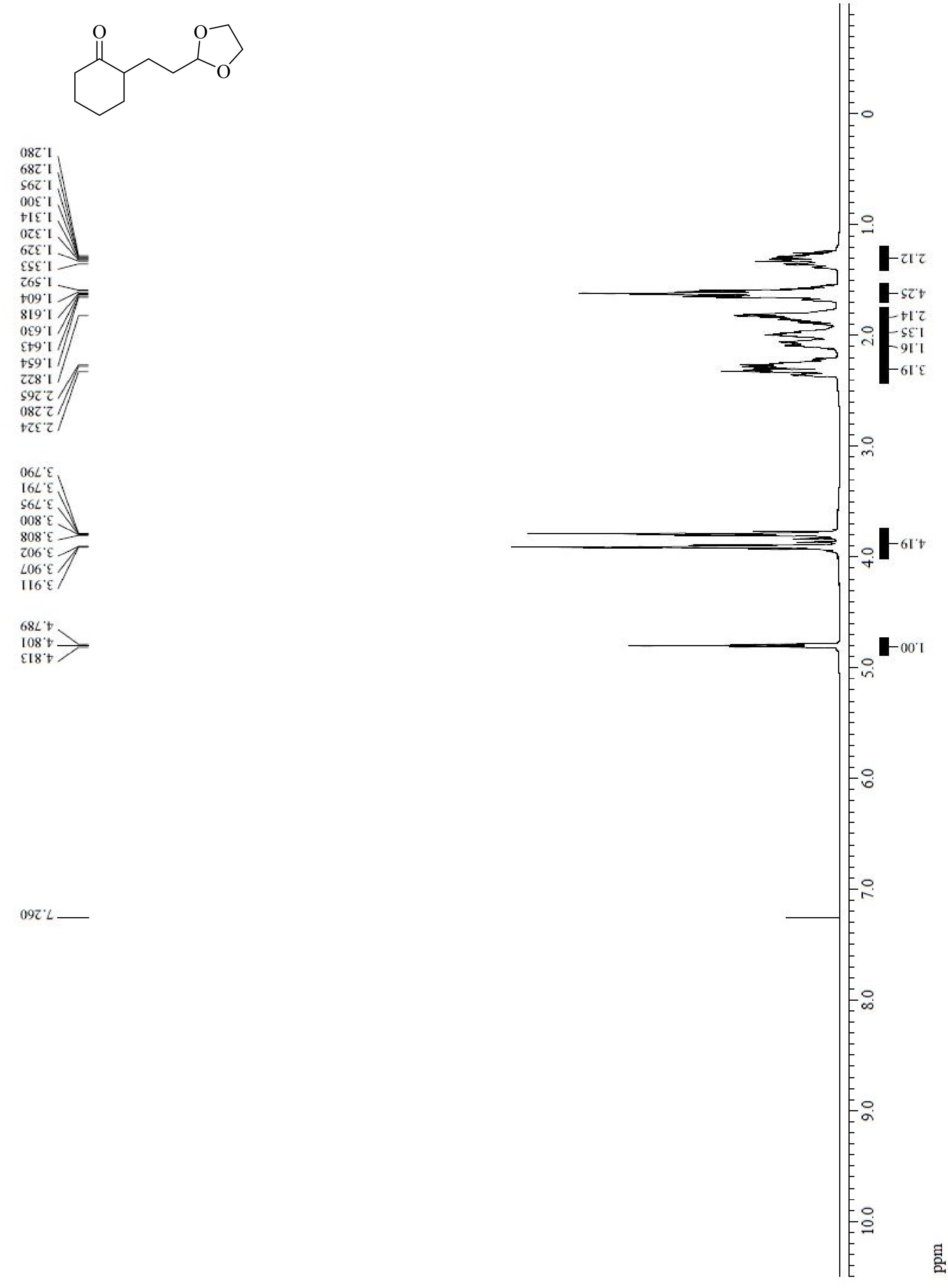

6a, $^{13}C\{^{1}H\}$ NMR (101MHz, $CDCl_3$)

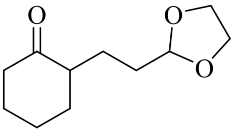

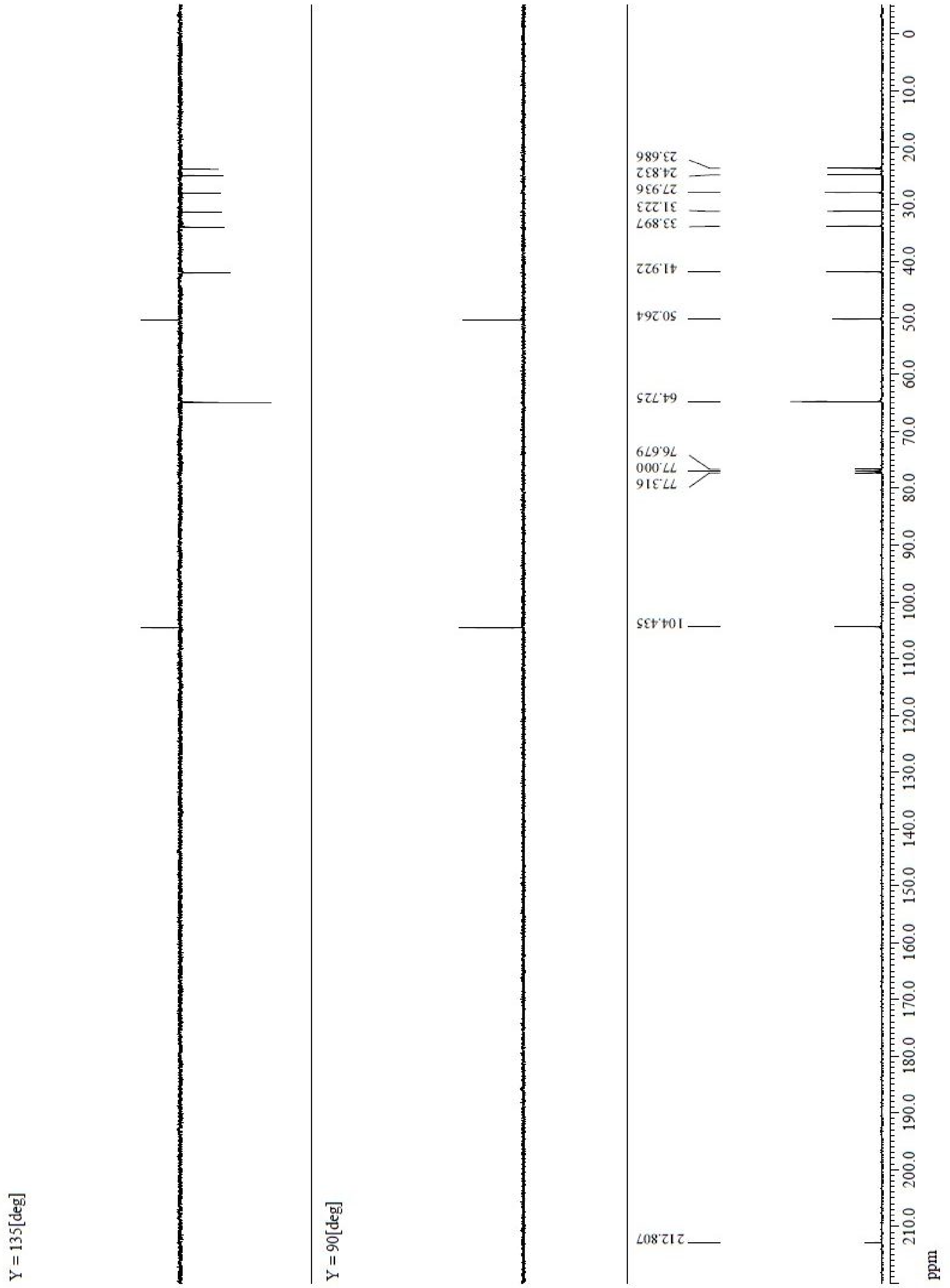

6b, ^{1}H-NMR (400MHz, $CDCl_3$)

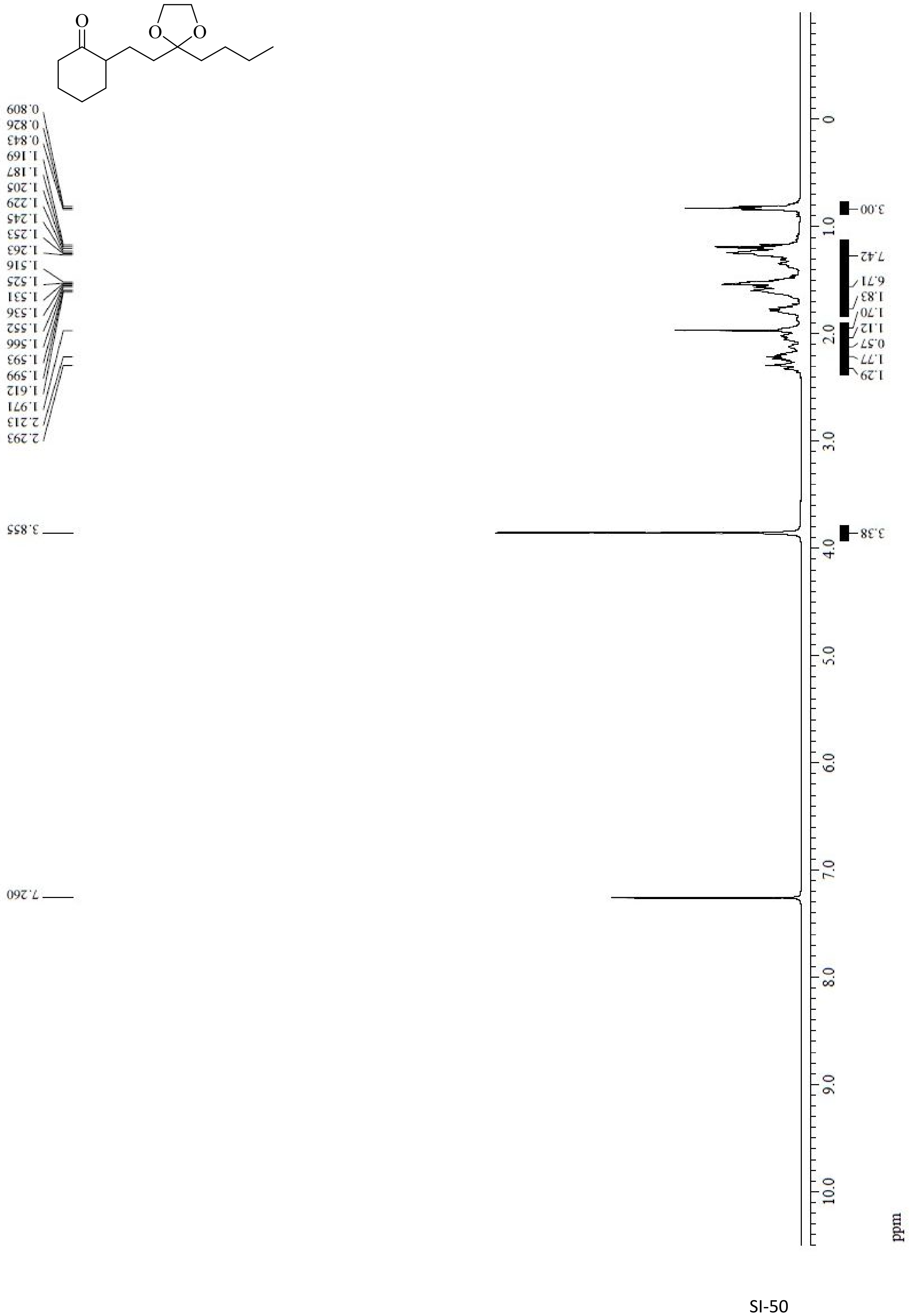

6b, $^{13}C\{^1H\}$ NMR (101MHz, $CDCl_3$)

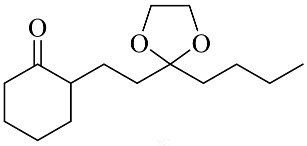

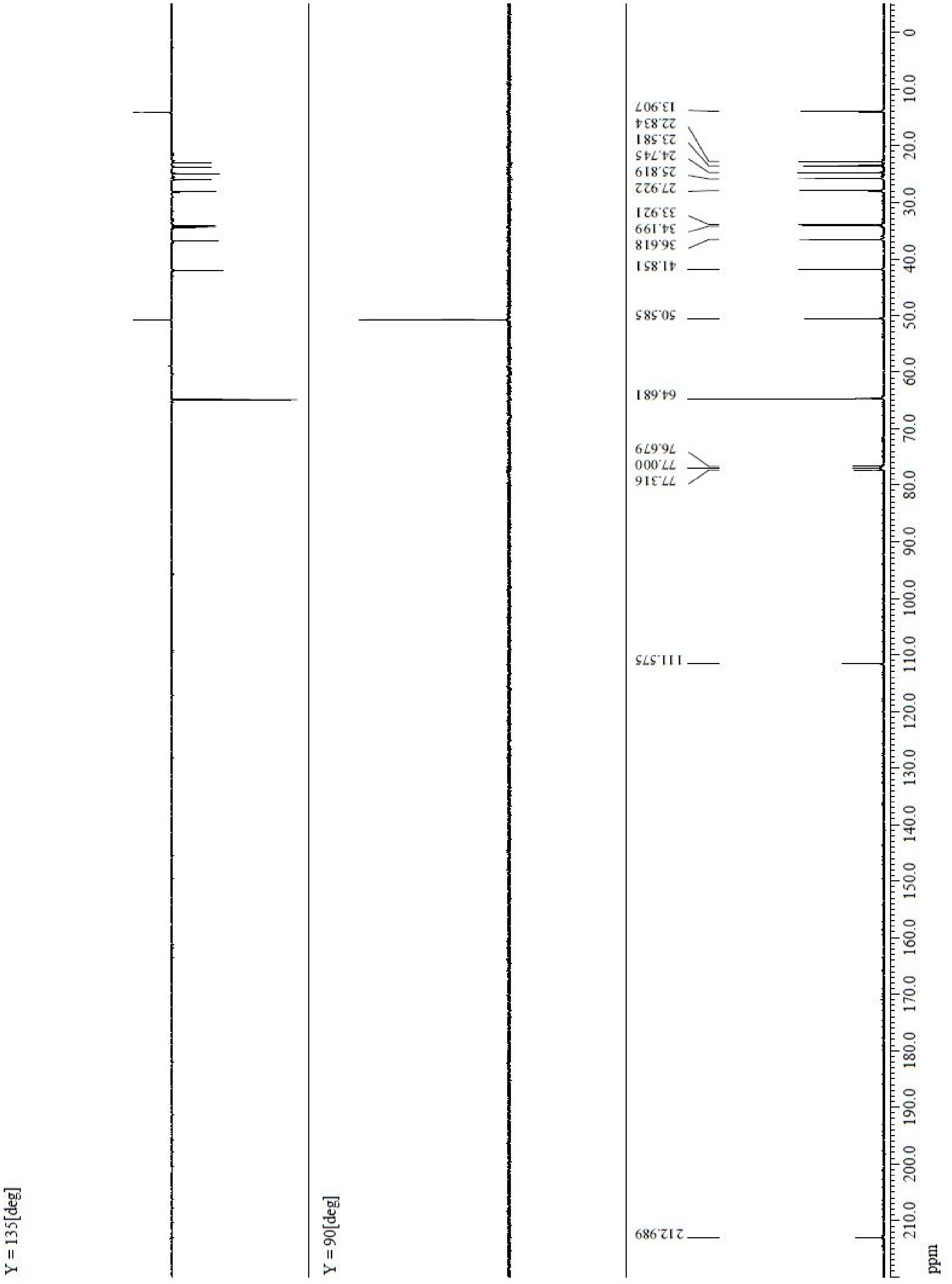

7b, ^{1}H-NMR (400MHz, $CDCl_3$)

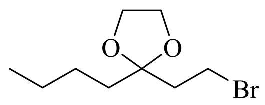

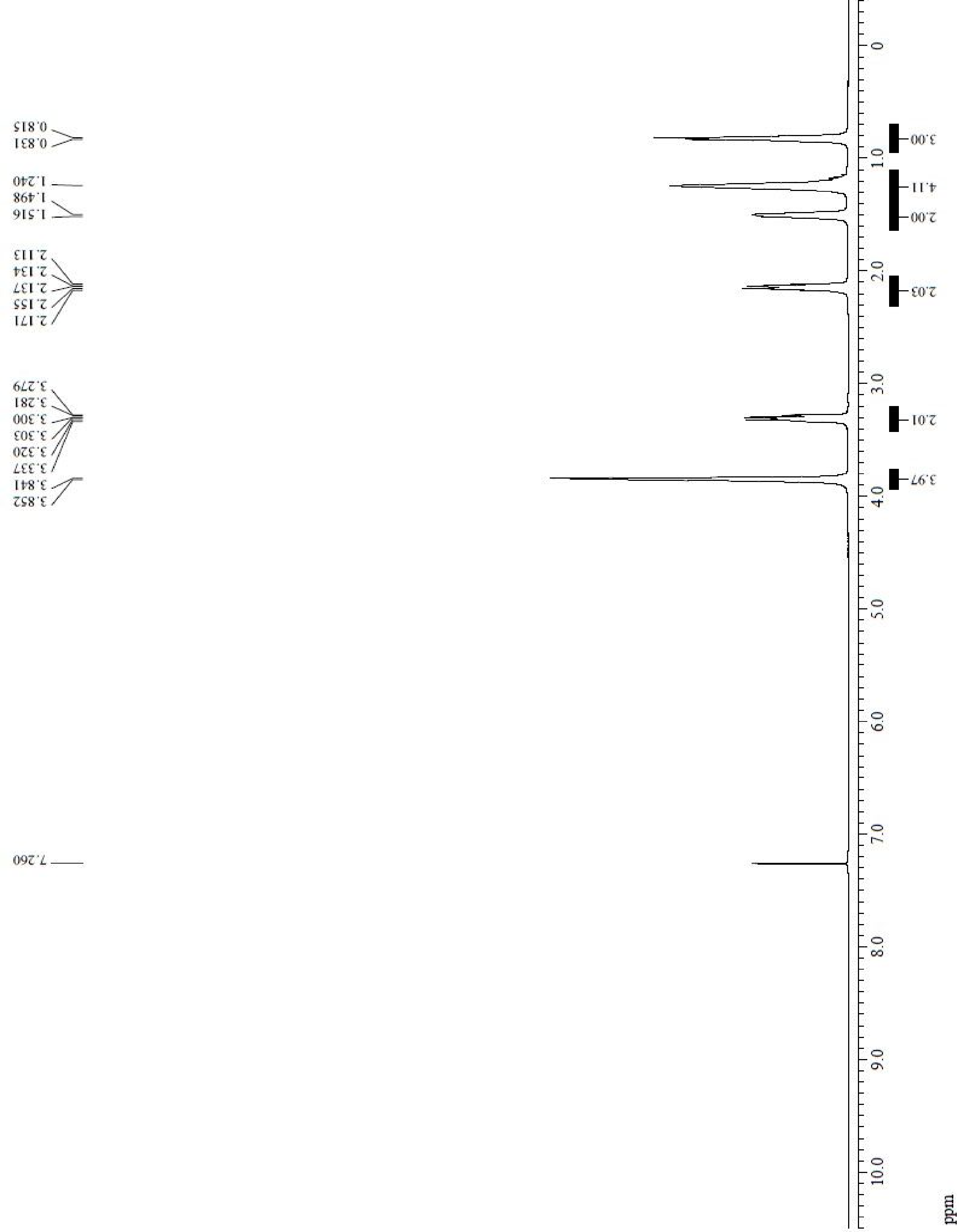

7b, $^{13}C\{^{1}H\}$ NMR (101MHz, $CDCl_3$)

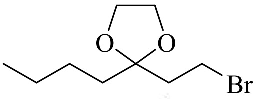

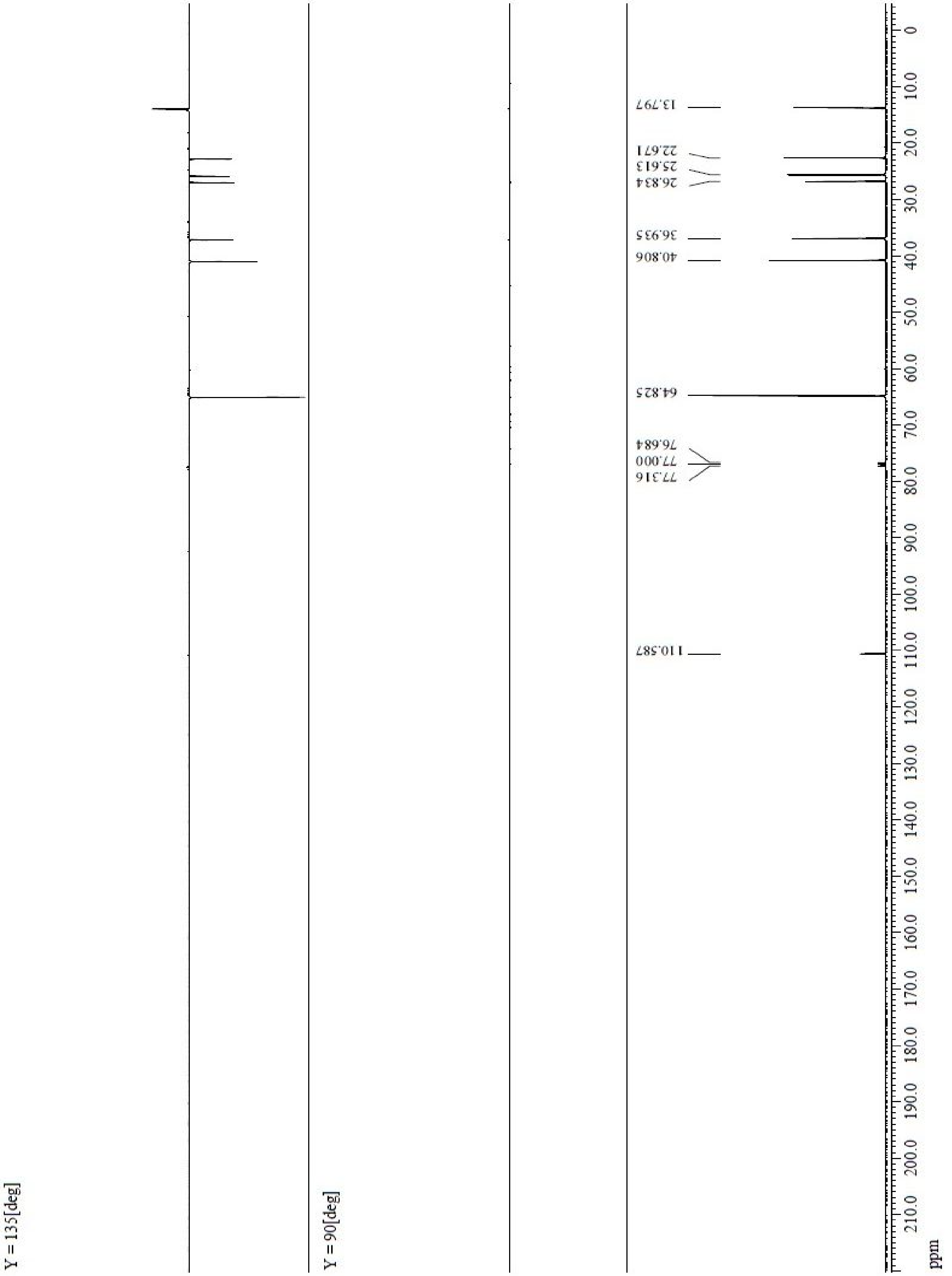

8a, ^{1}H-NMR (400MHz, $CDCl_3$)

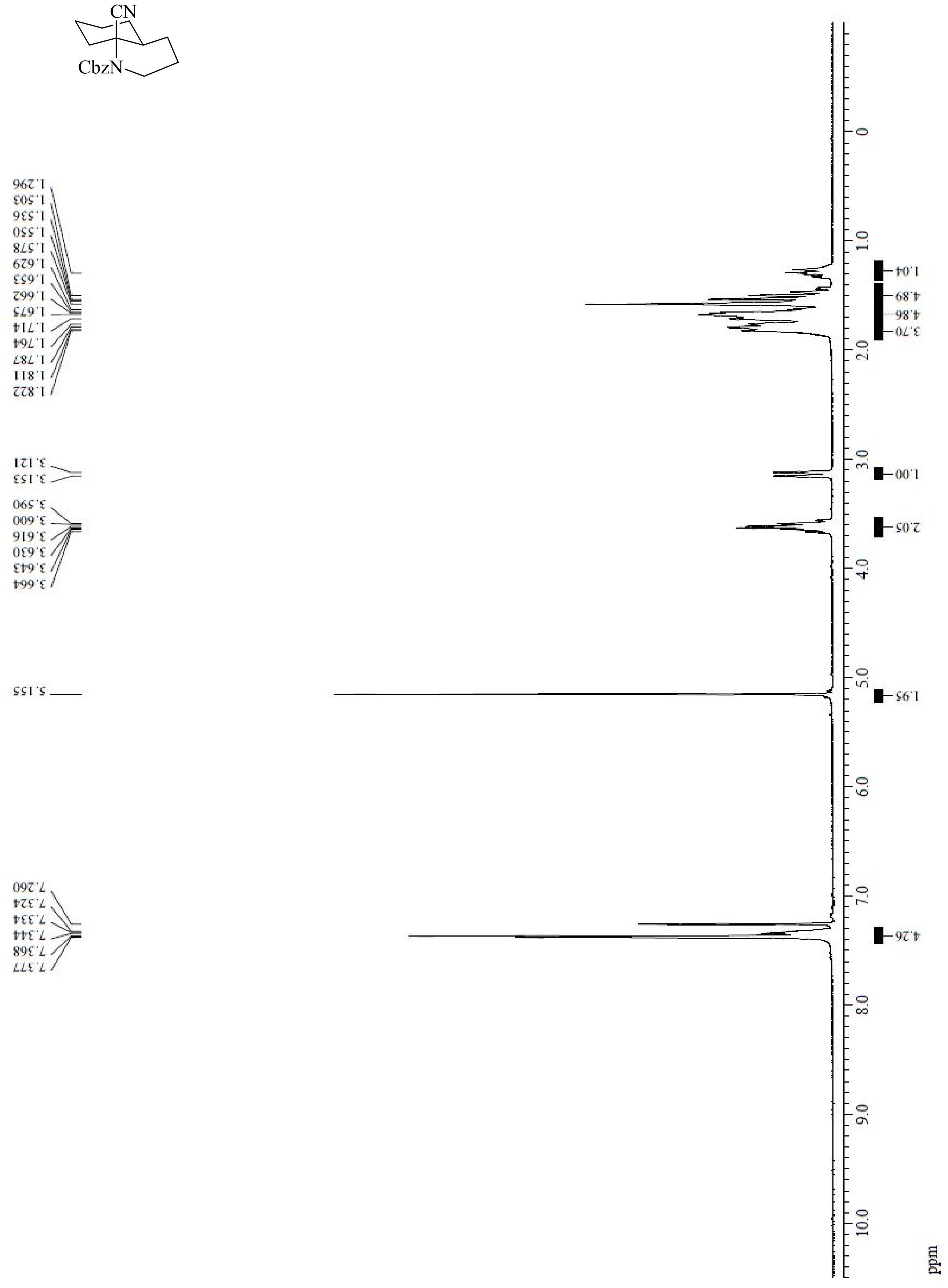

8a, $^{13}C\{^{1}H\}$ NMR (101MHz, $CDCl_3$)

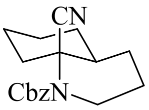

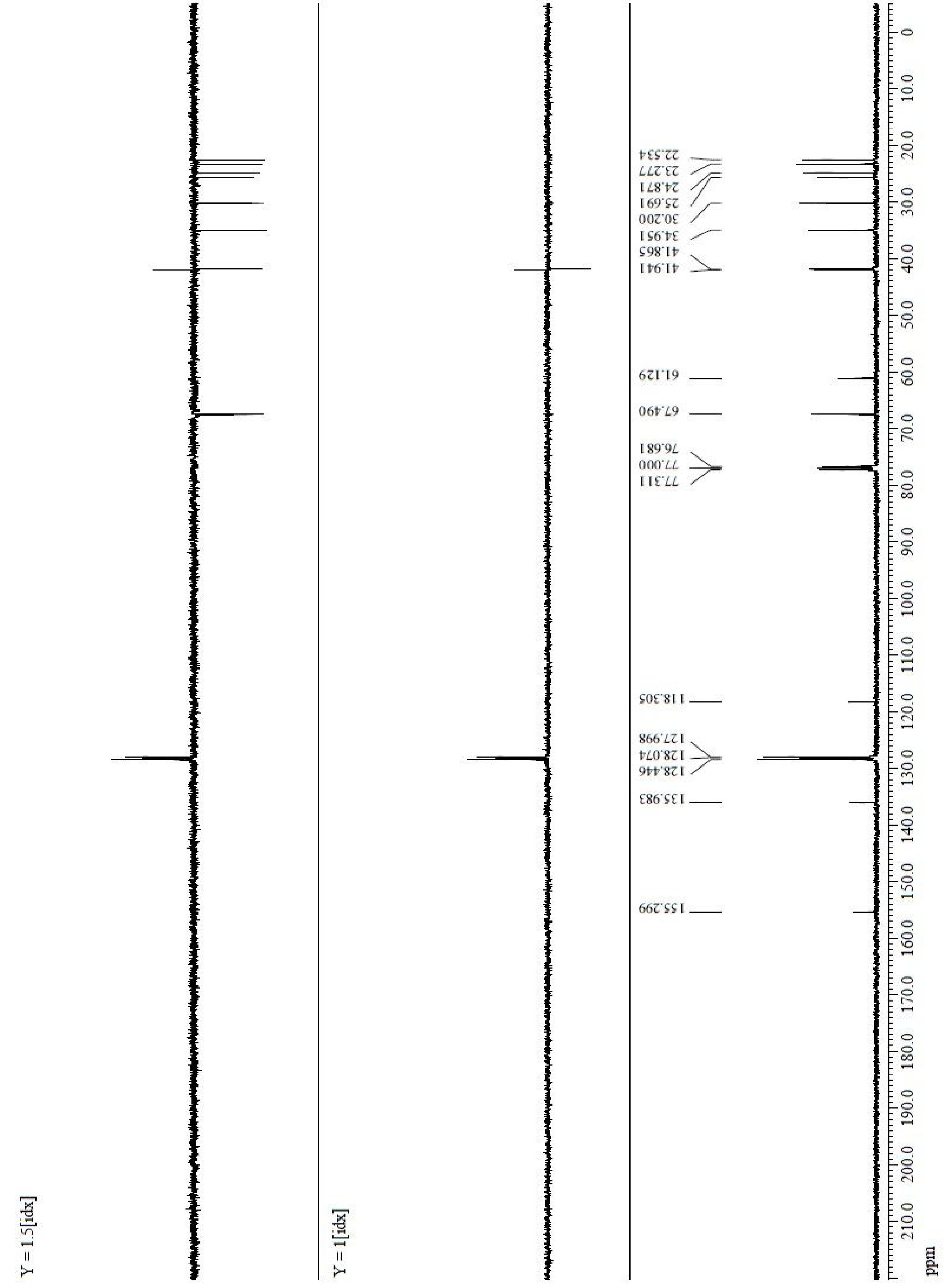

8b, ^{1}H-NMR (400MHz, $CDCl_3$)

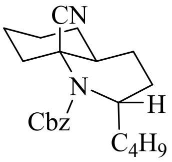

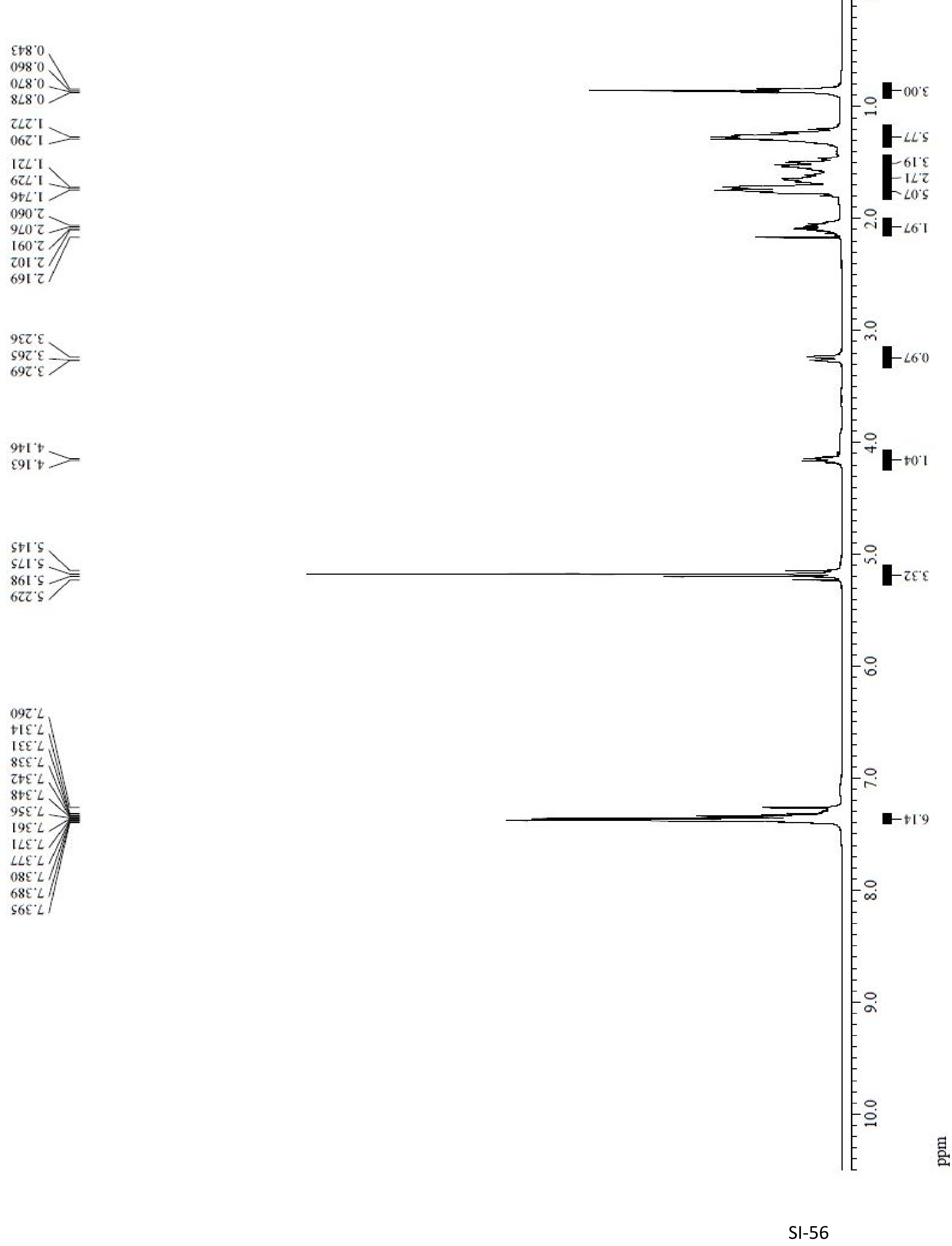

8b, $^{13}C\{^{1}H\}$ NMR (101MHz, $CDCl_3$)

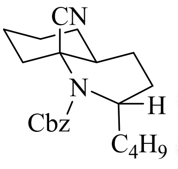

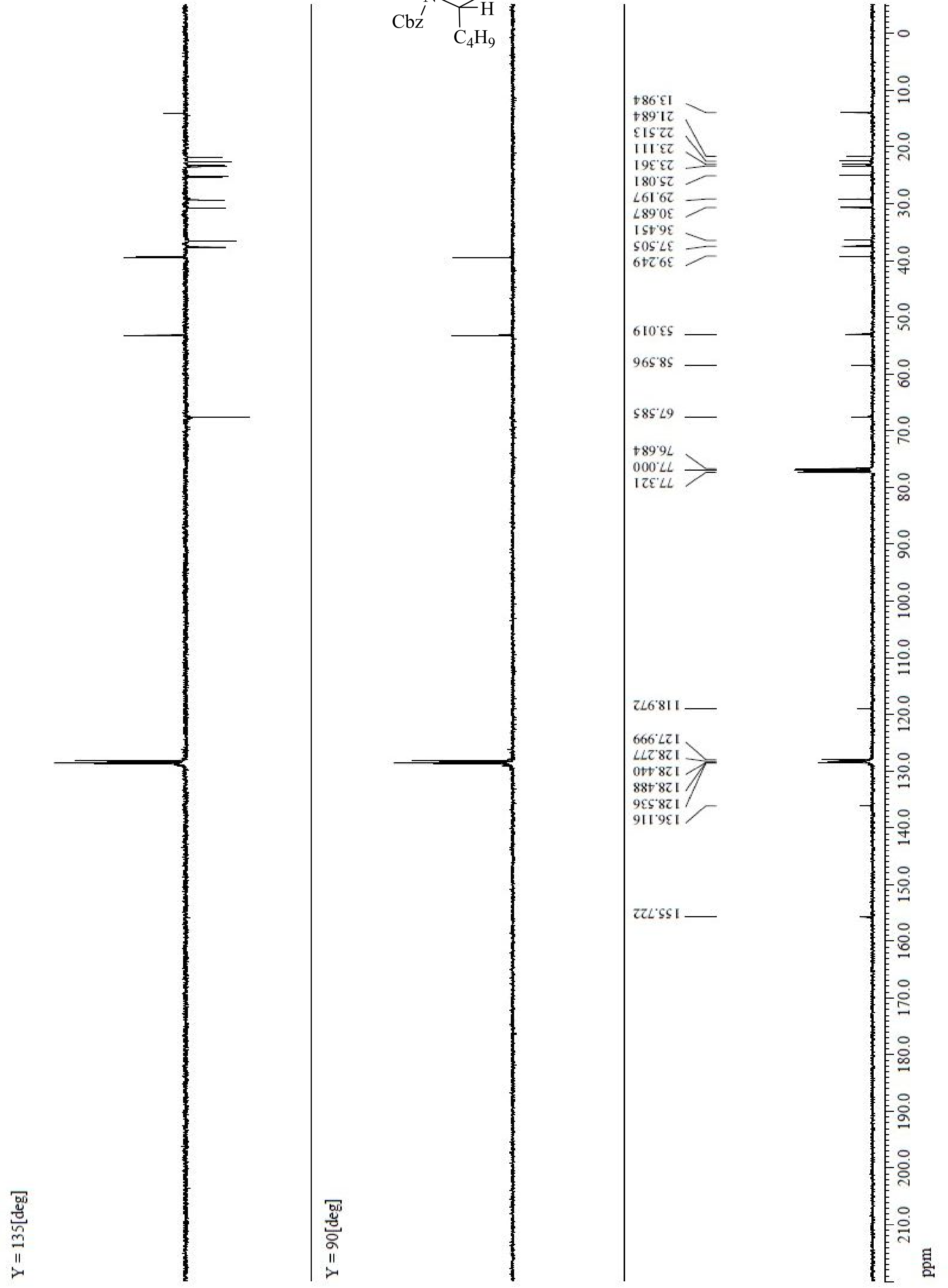

9, ^{1}H-NMR (400MHz, $CDCl_3$)

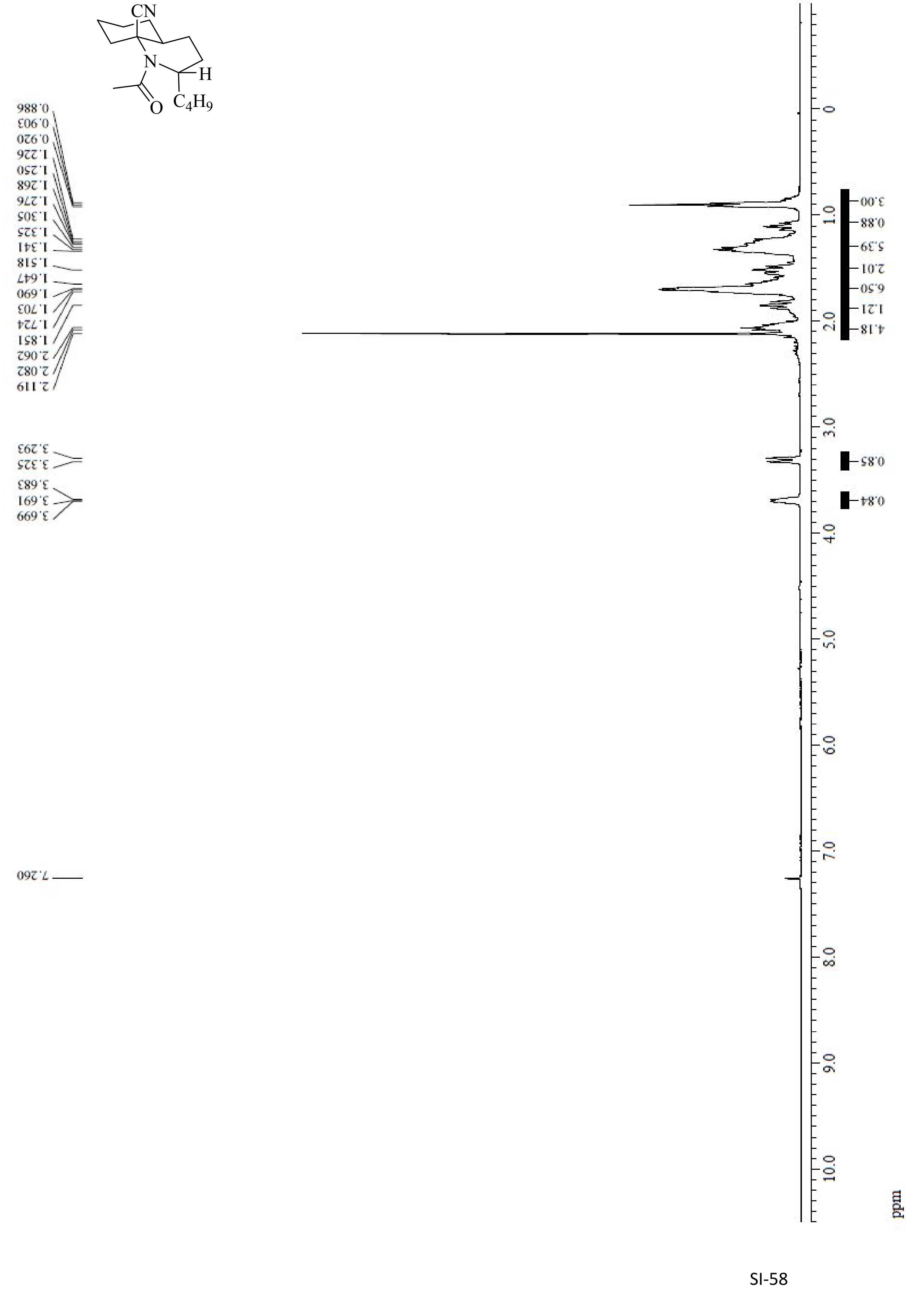

9, $^{13}C\{^1H\}$ NMR (101MHz, $CDCl_3$)

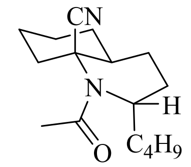

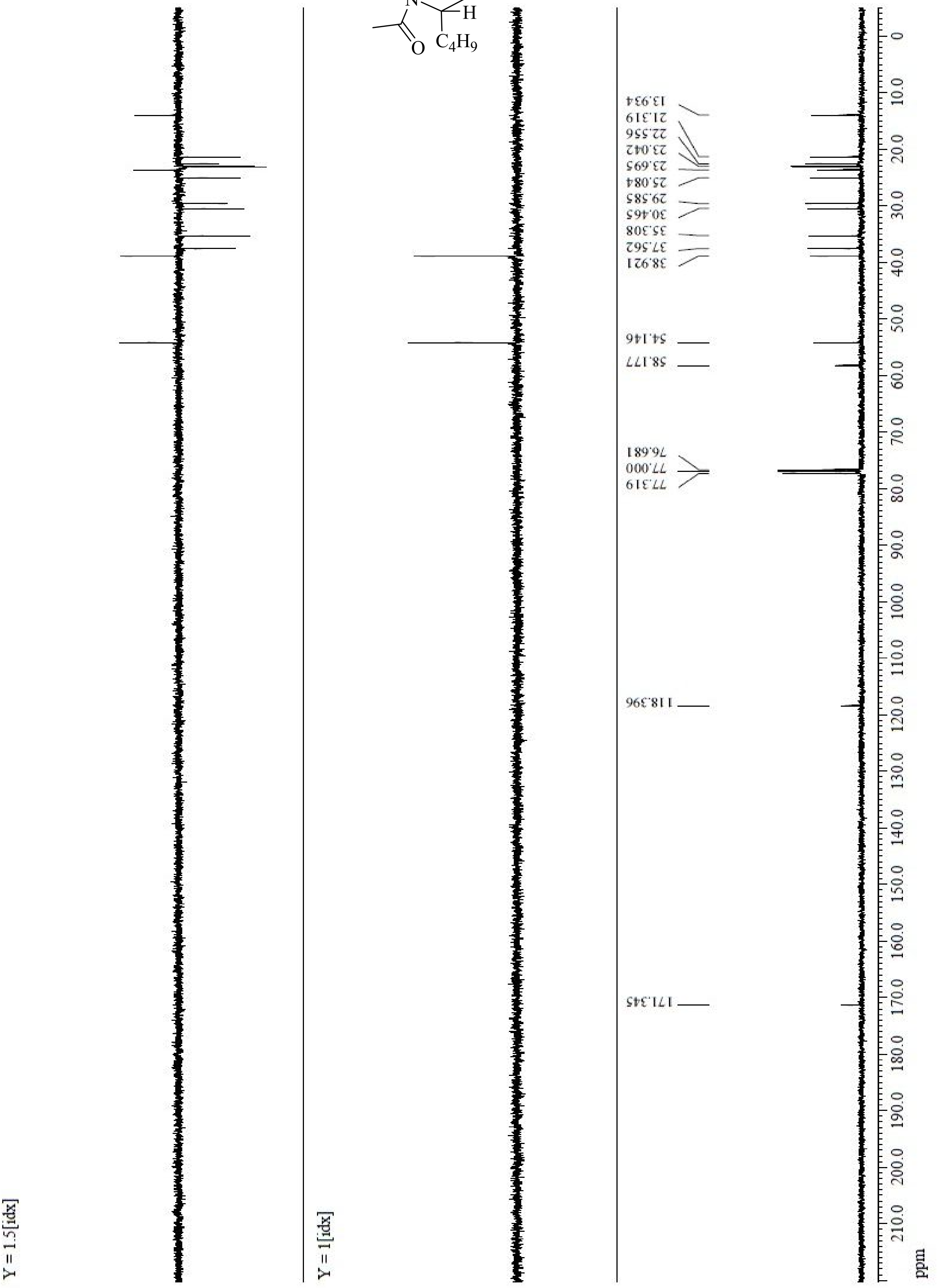

10, ^{1}H-NMR (400MHz, $CDCl_3$)

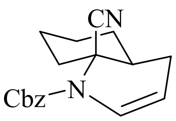

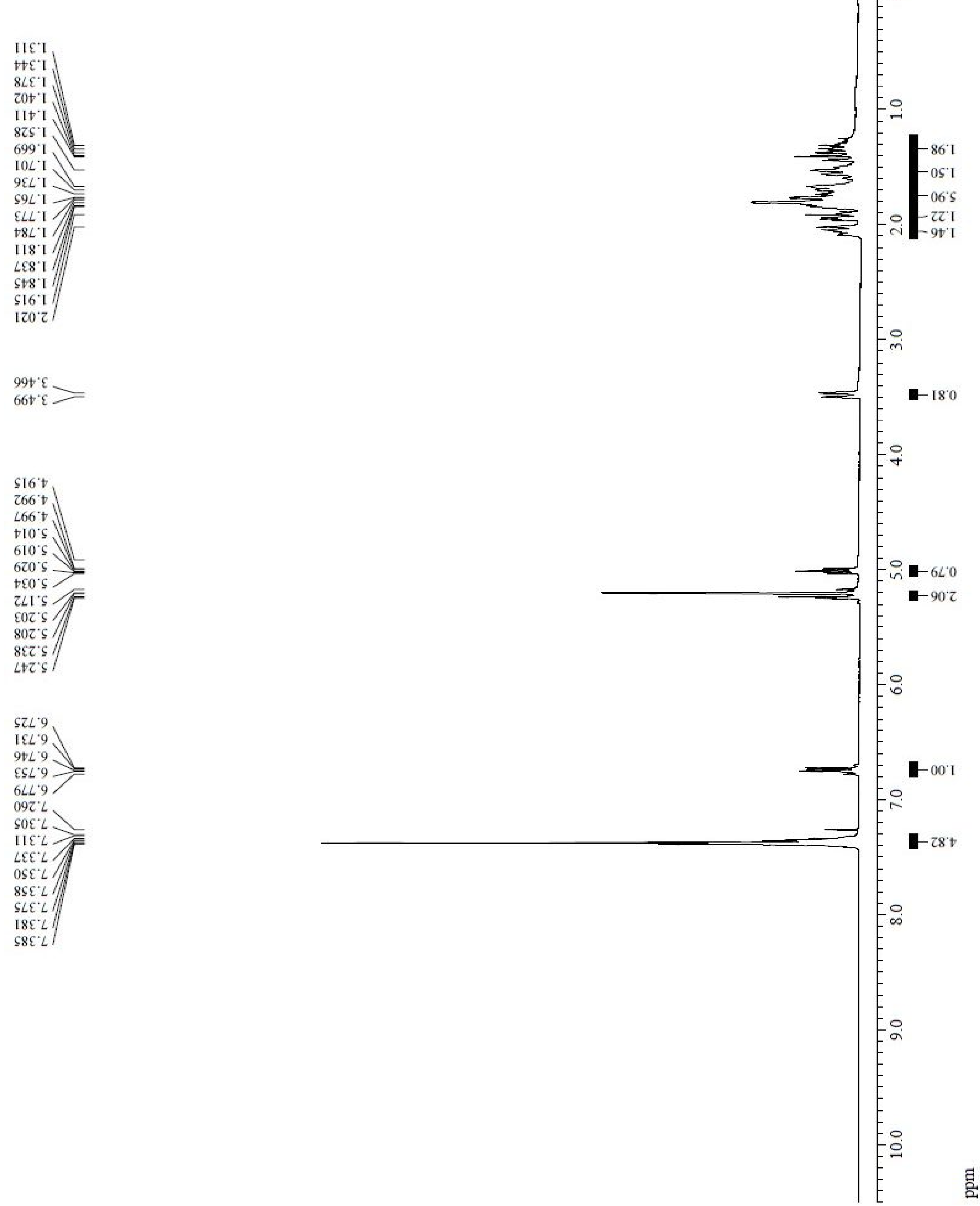

10, $^{13}C\{^{1}H\}$ NMR (101MHz, $CDCl_3$)

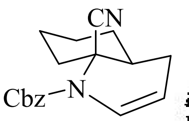

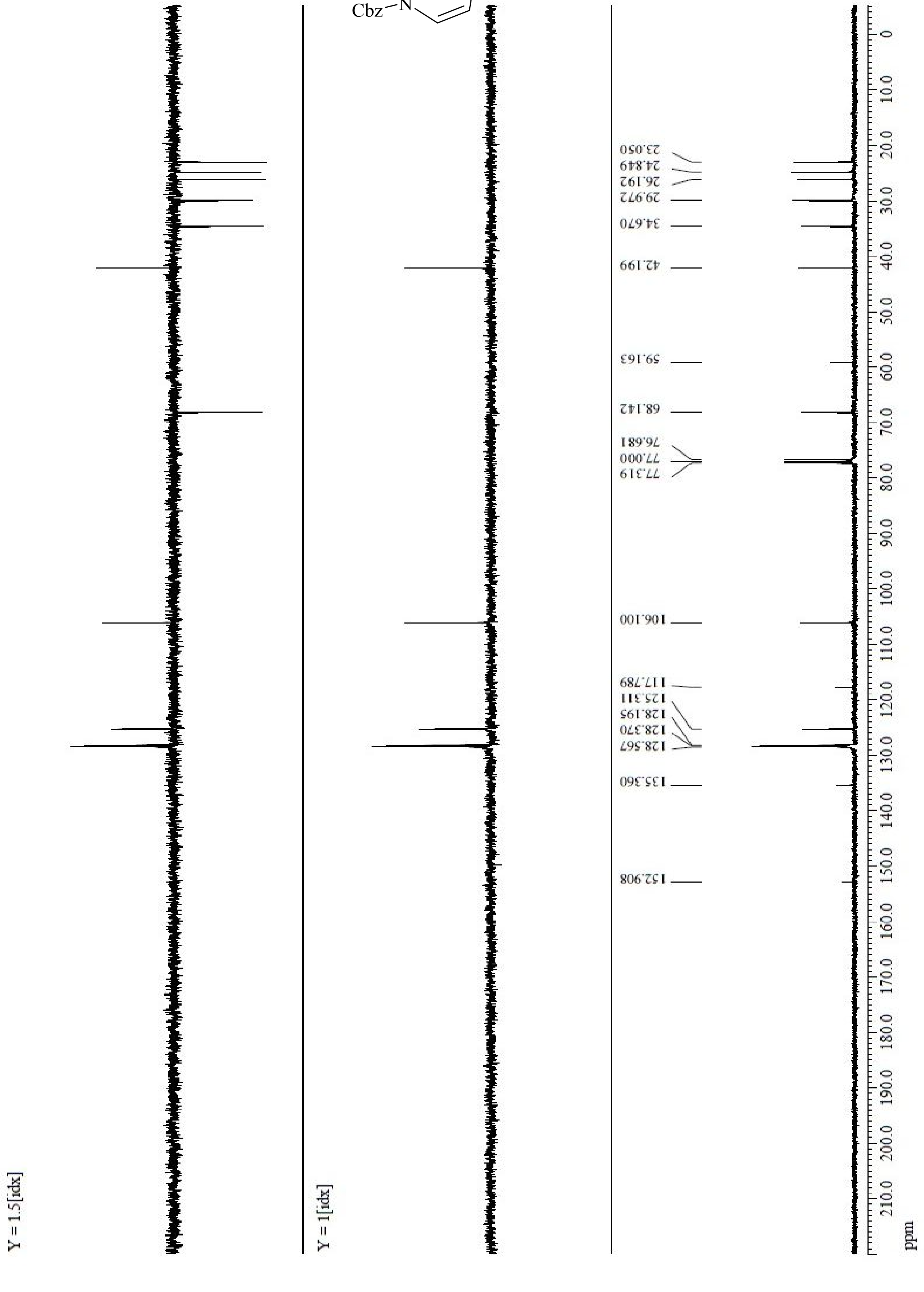

11, ^{1}H-NMR (400MHz, $CDCl_3$)

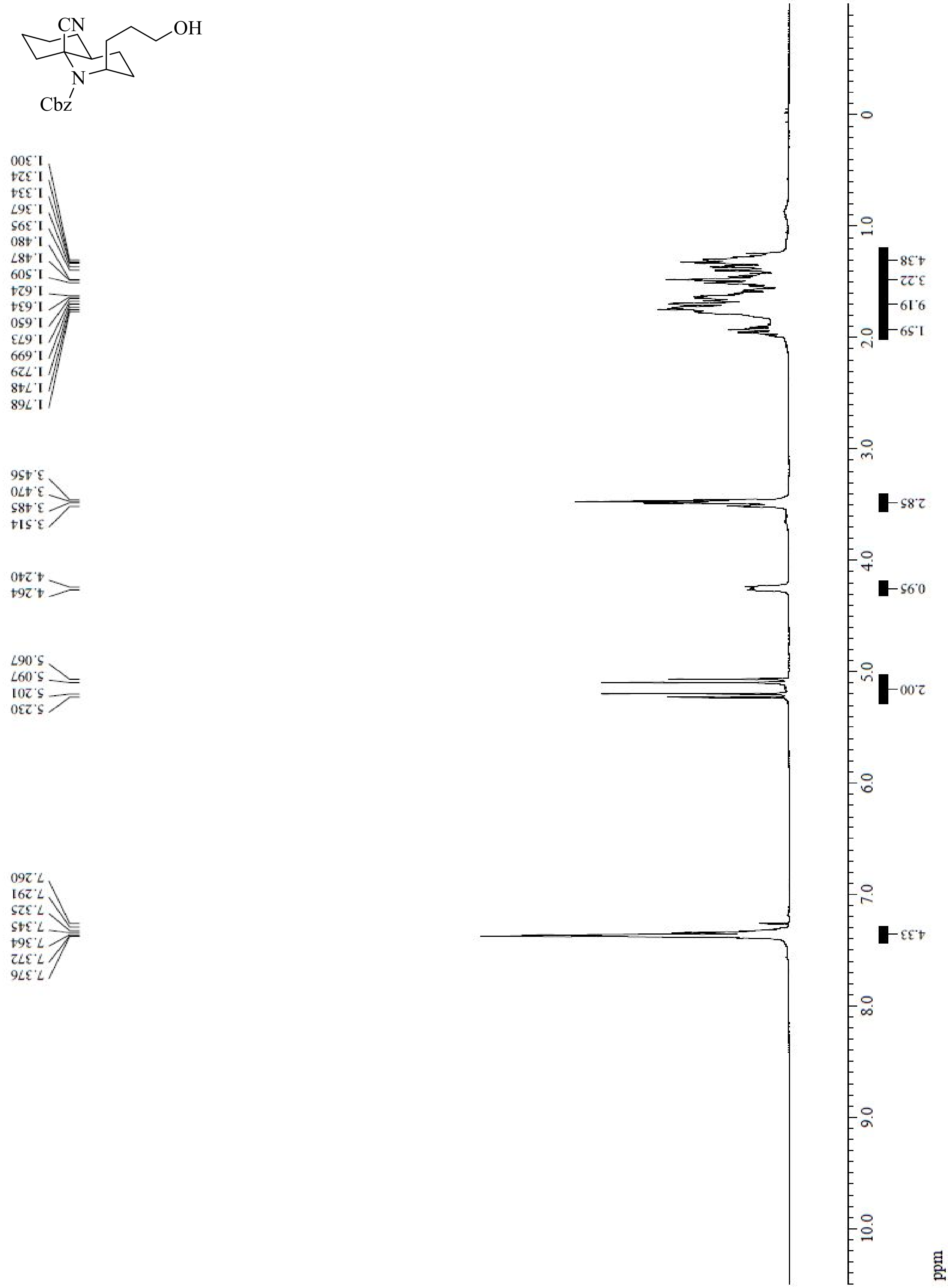

11, $^{13}C\{^1H\}$ NMR (101MHz, $CDCl_3$)

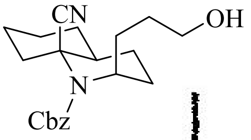

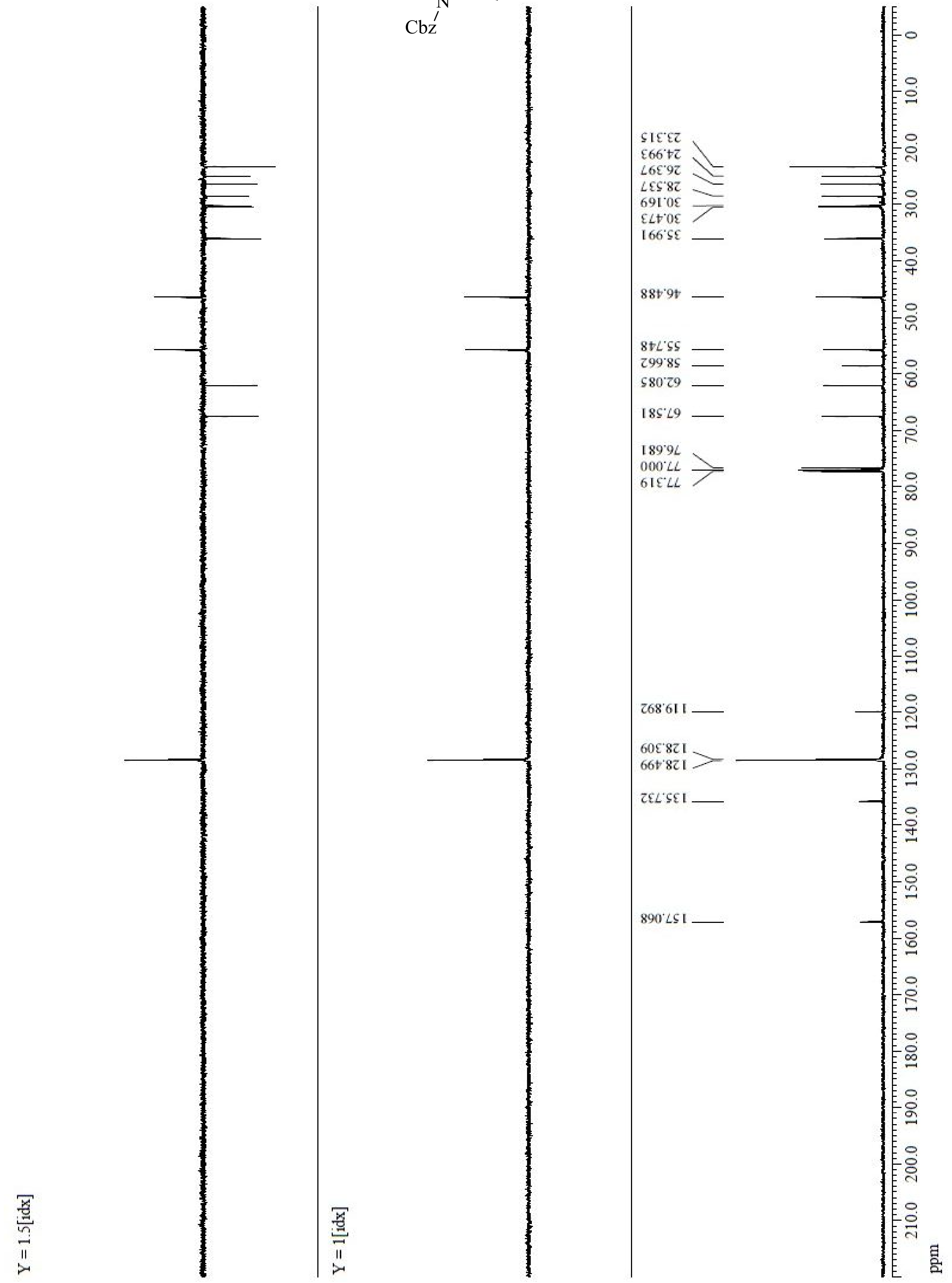

12, ^{1}H-NMR (400MHz, $CDCl_3$)

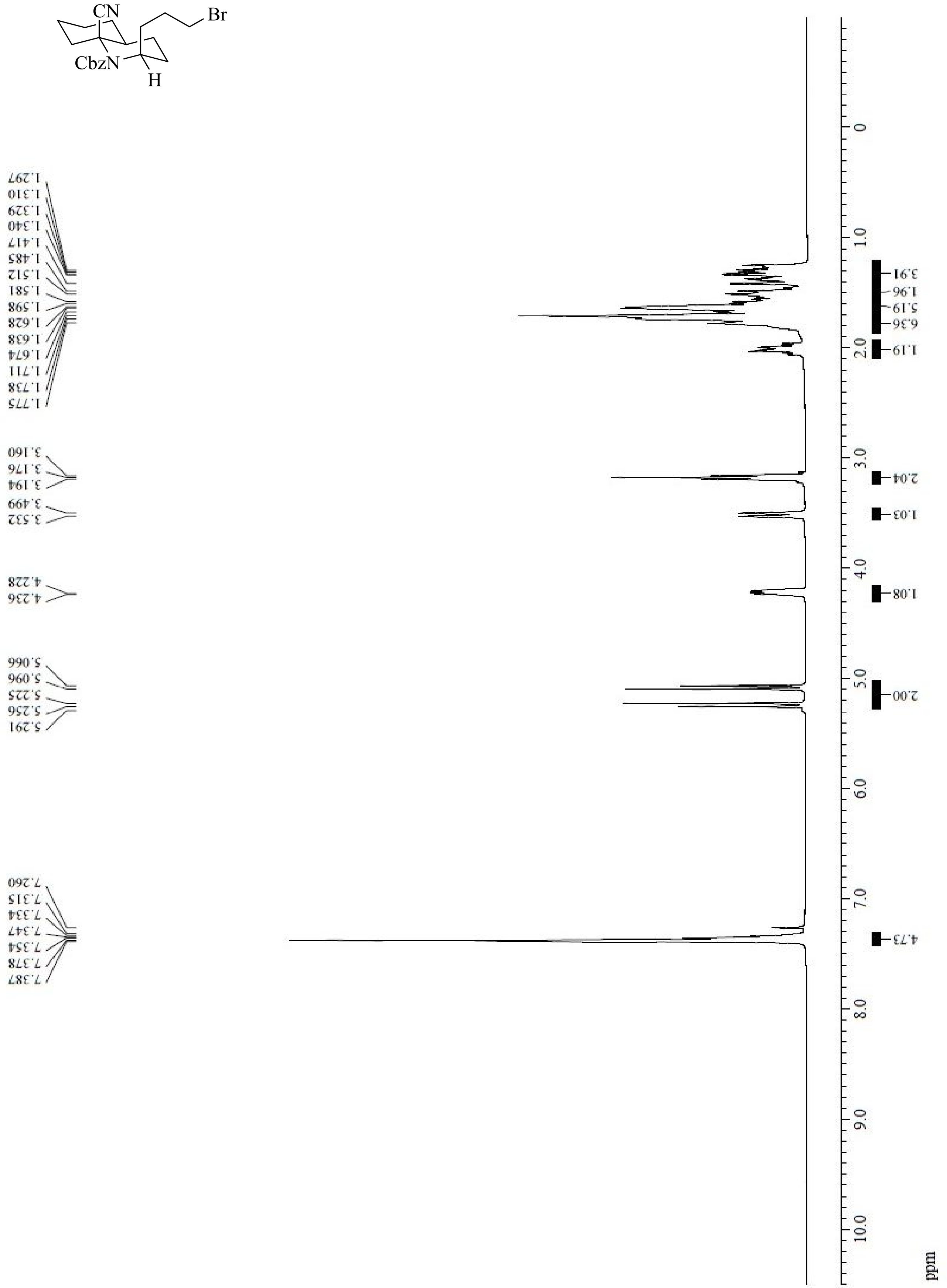

12, $^{13}C\{^1H\}$ NMR (101MHz, $CDCl_3$)

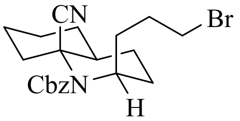

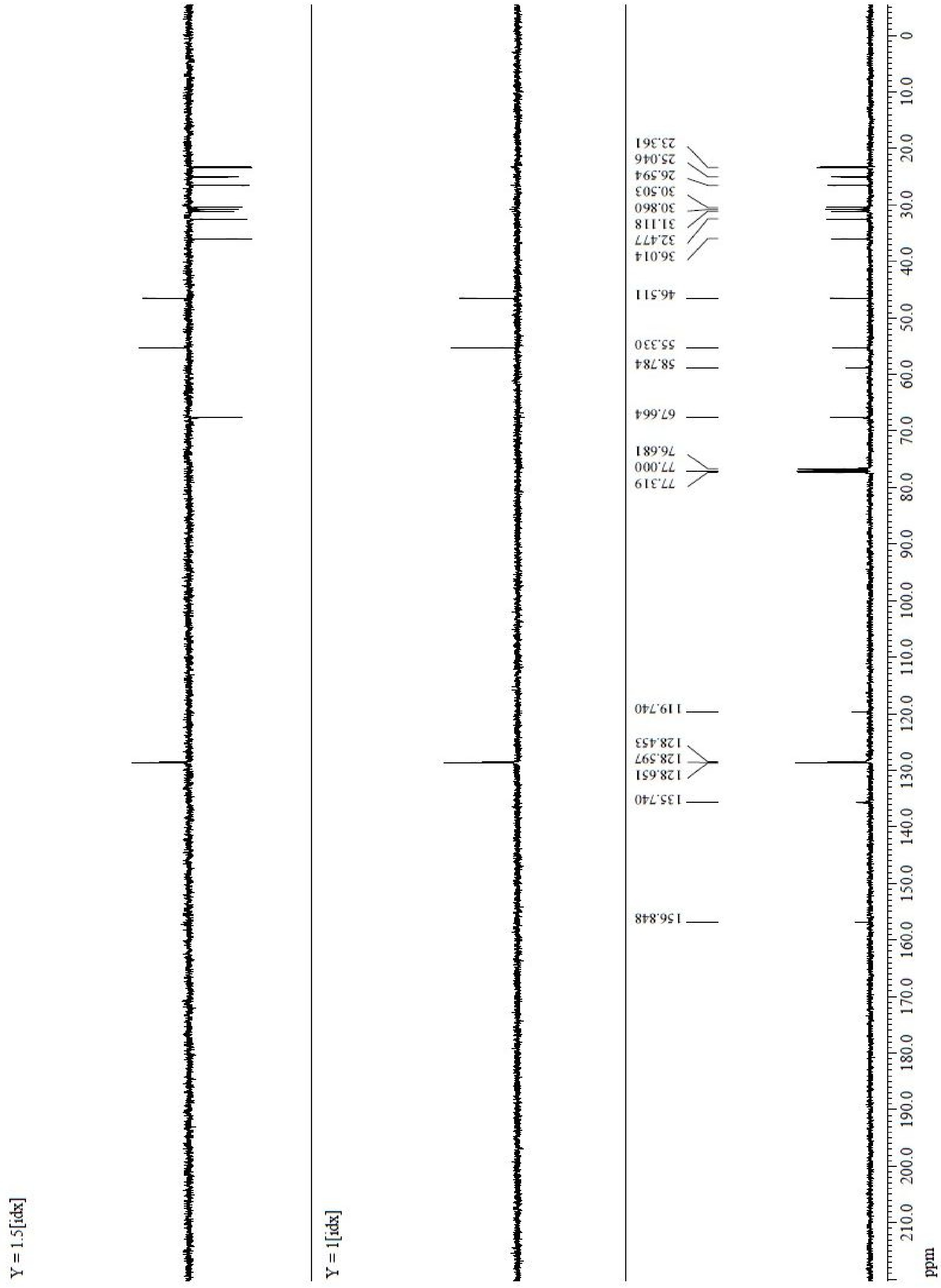

13, ^{1}H-NMR (400MHz, $CDCl_3$)

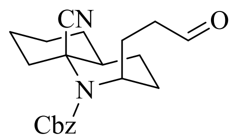

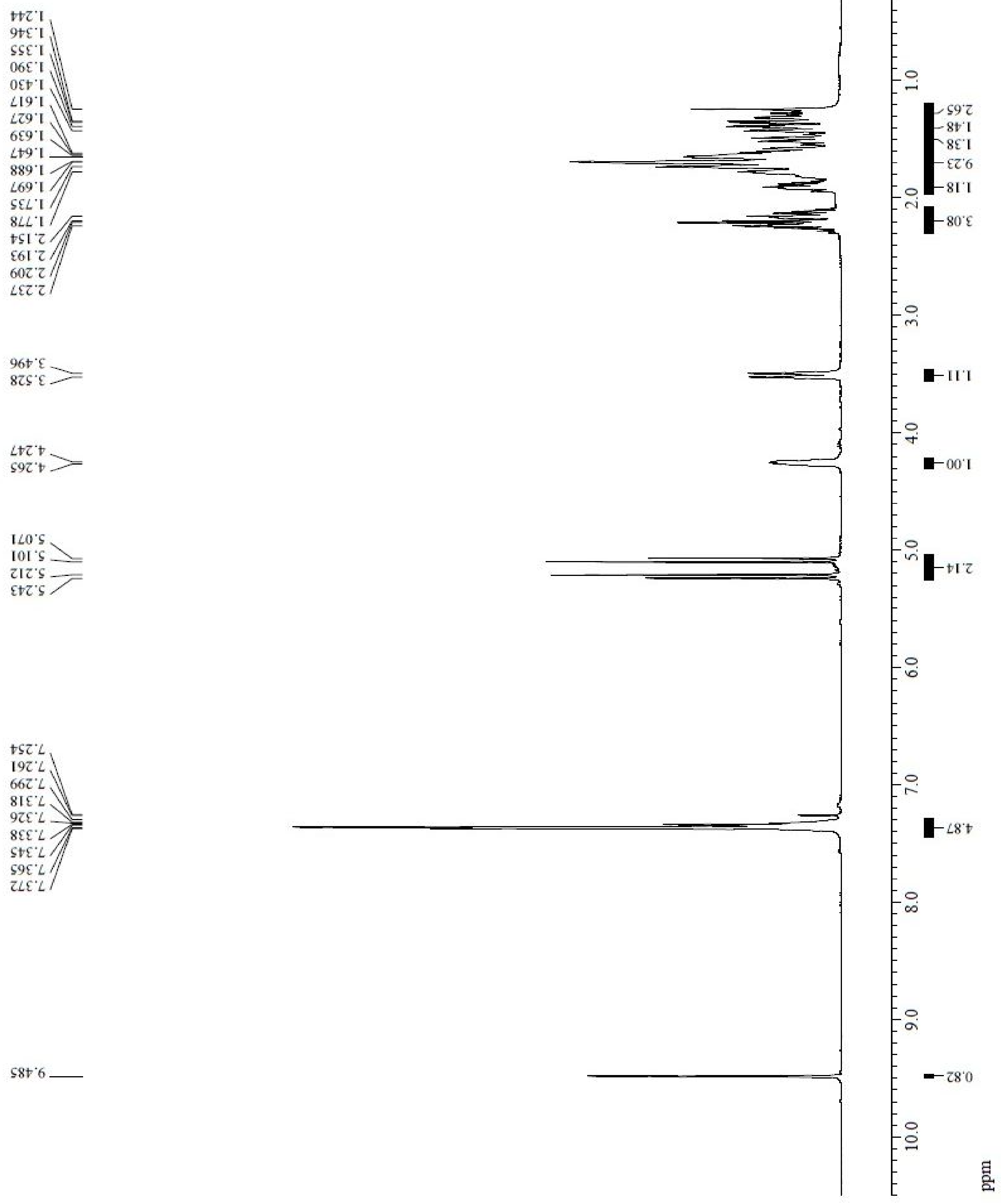

13, $^{13}C\{^{1}H\}$ NMR (101MHz, $CDCl_3$)

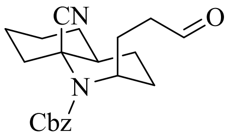

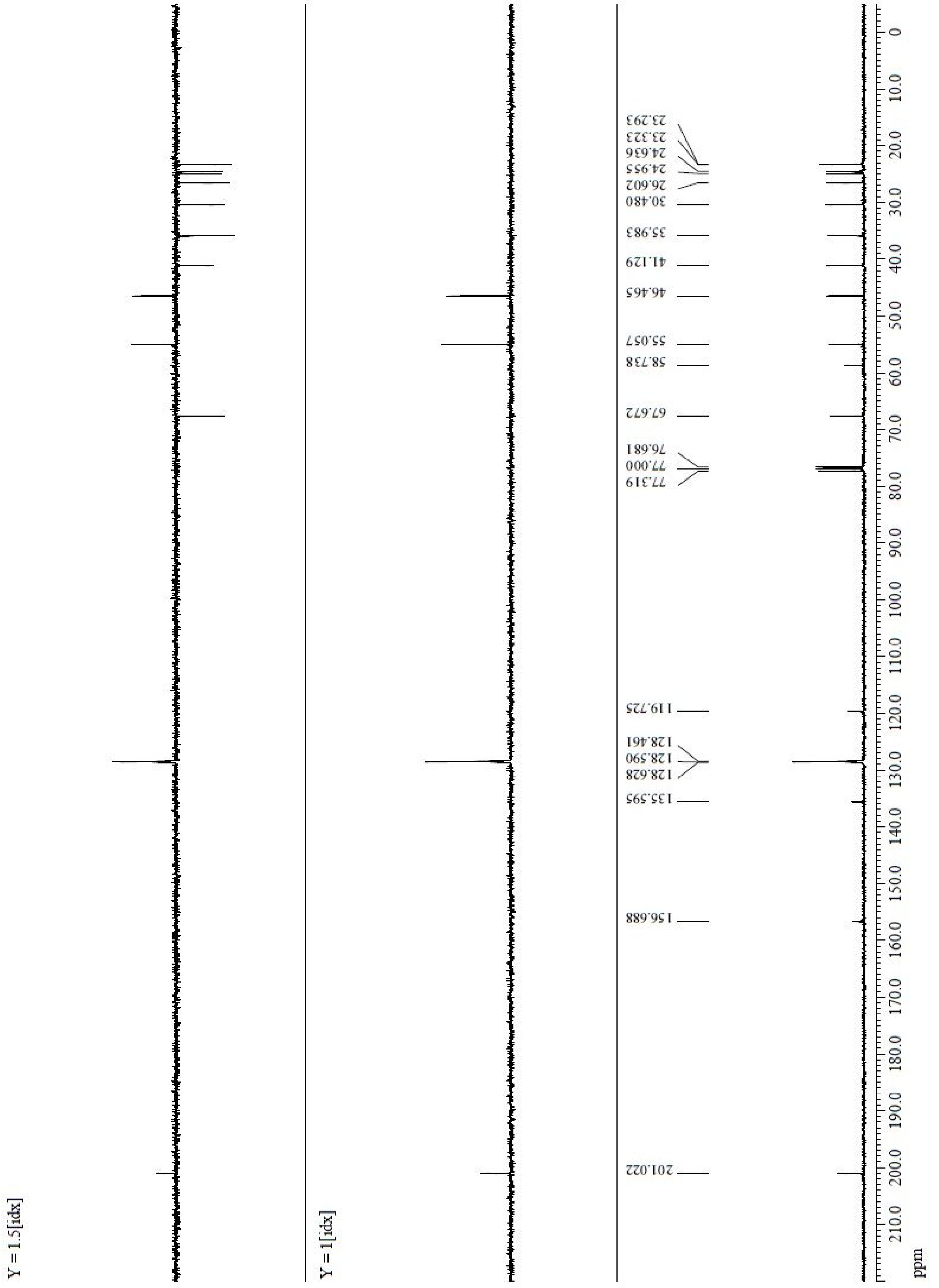

14, ^{1}H-NMR (400MHz, $CDCl_3$)

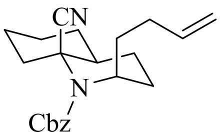

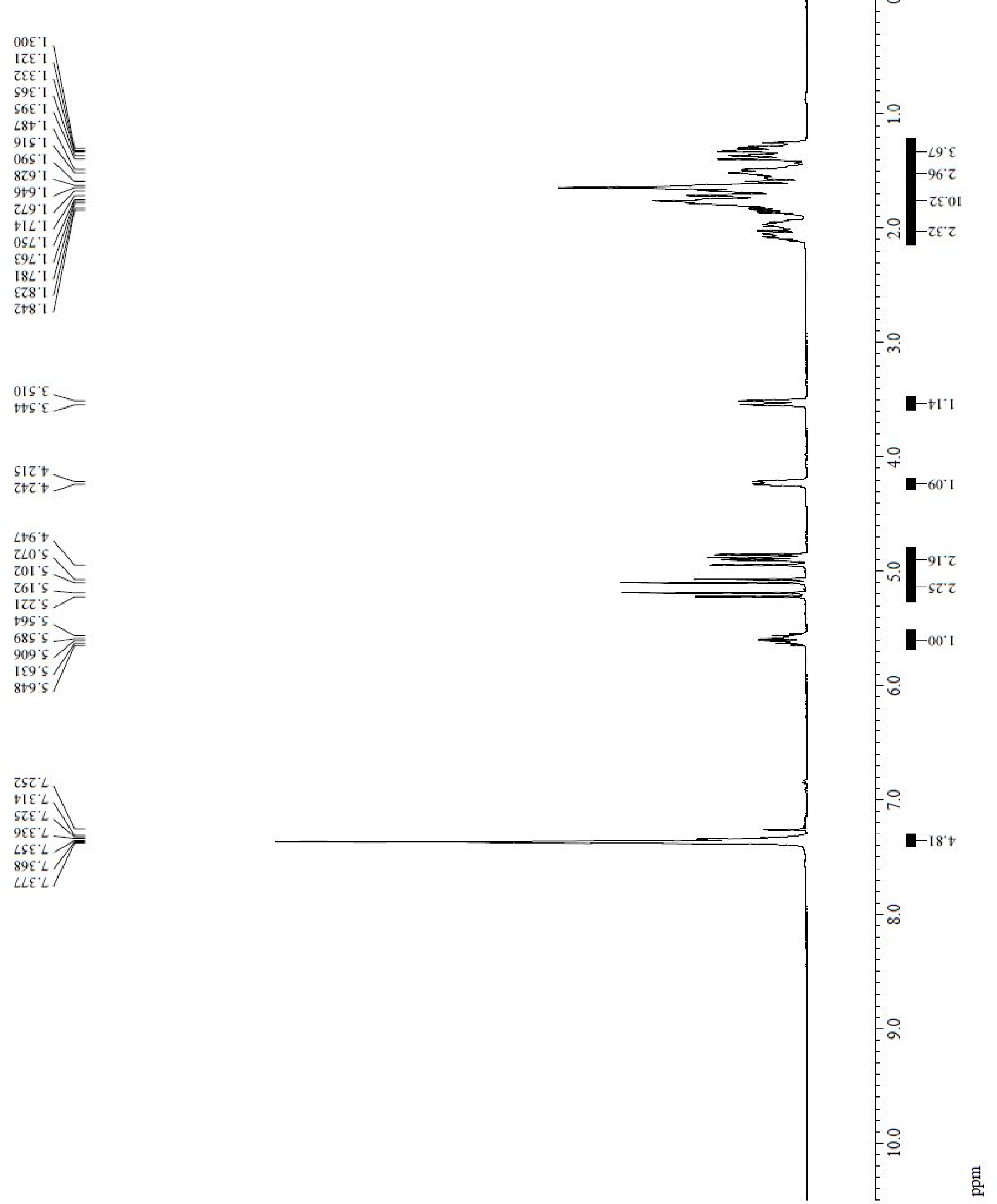

14, $^{13}C\{^{1}H\}$ NMR (101MHz, $CDCl_3$)

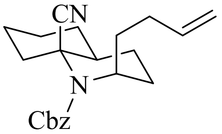

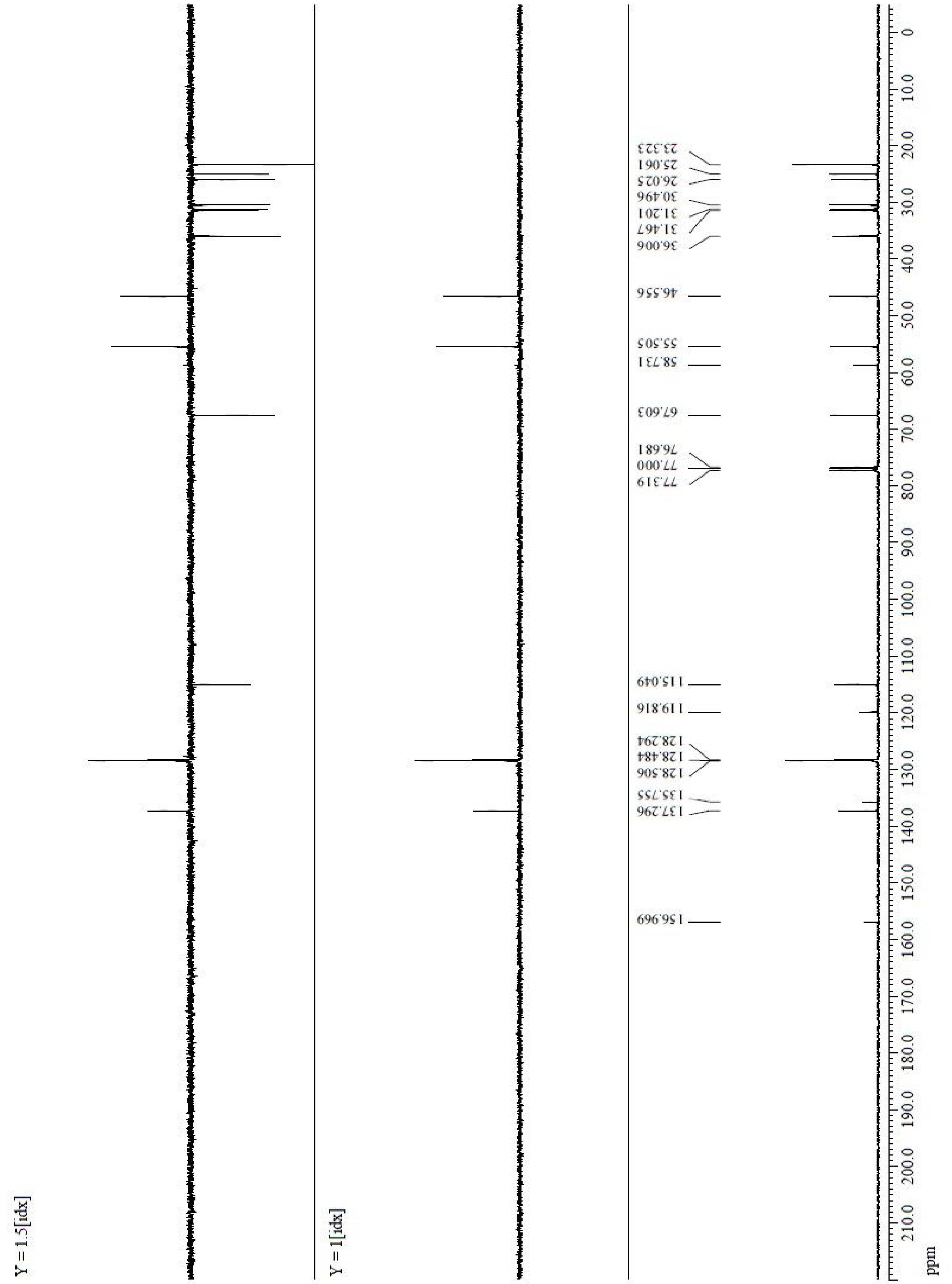

15, ^{1}H-NMR (400MHz, $CDCl_3$)

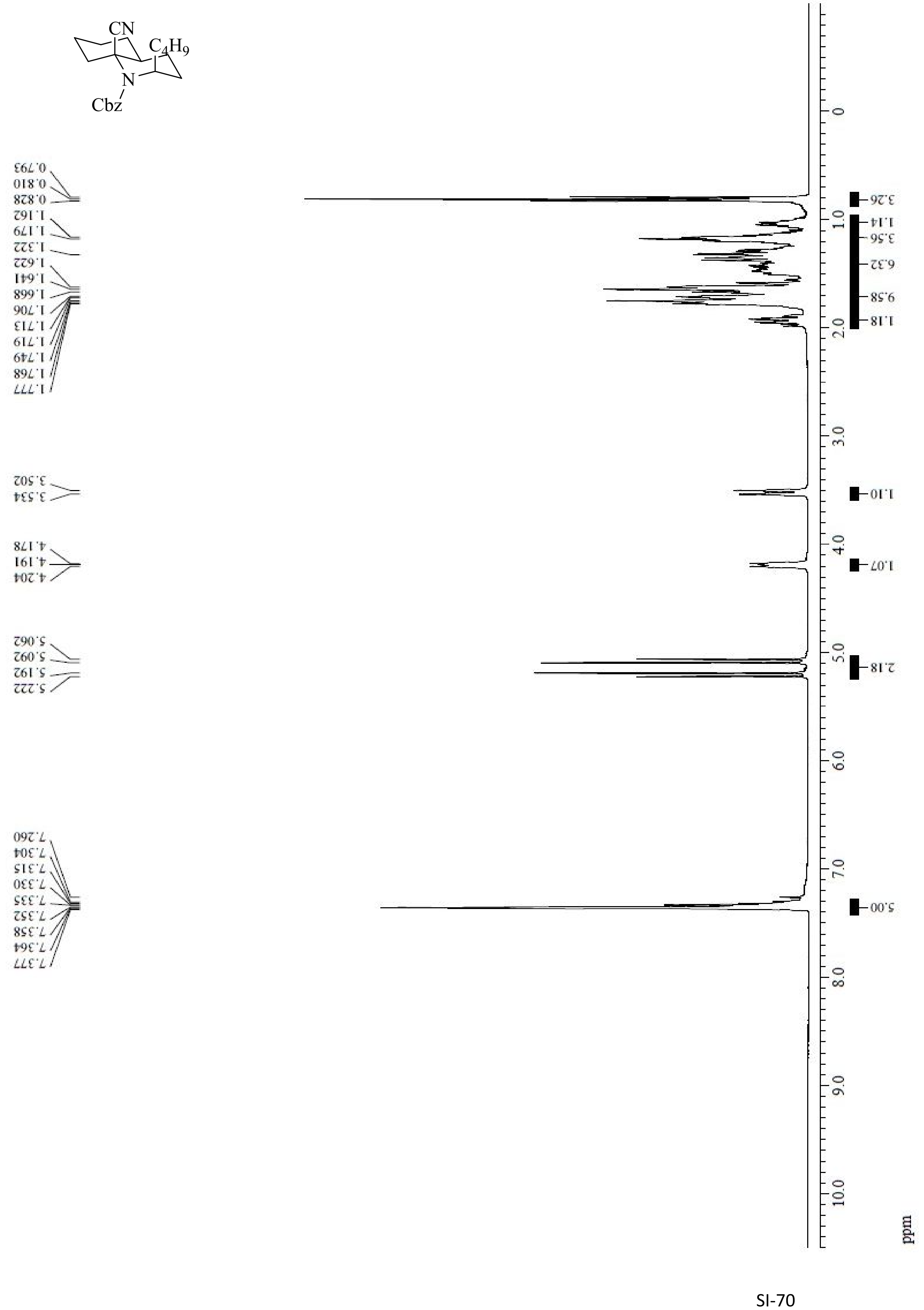

15, $^{13}C\{^{1}H\}$ NMR (101MHz, $CDCl_3$)

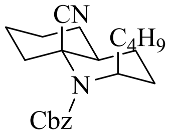

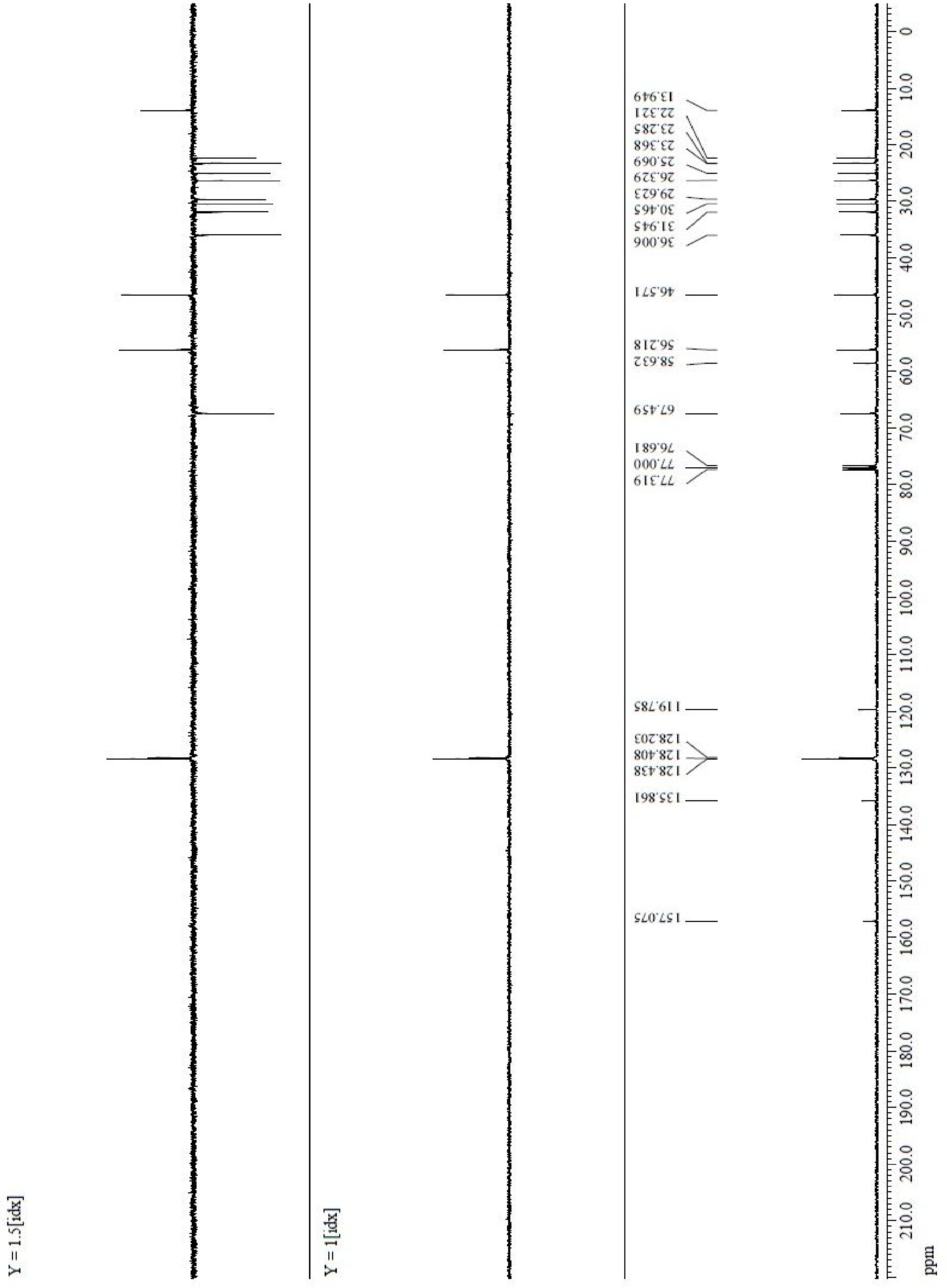

16, ^{1}H-NMR (400MHz, $CDCl_3$)

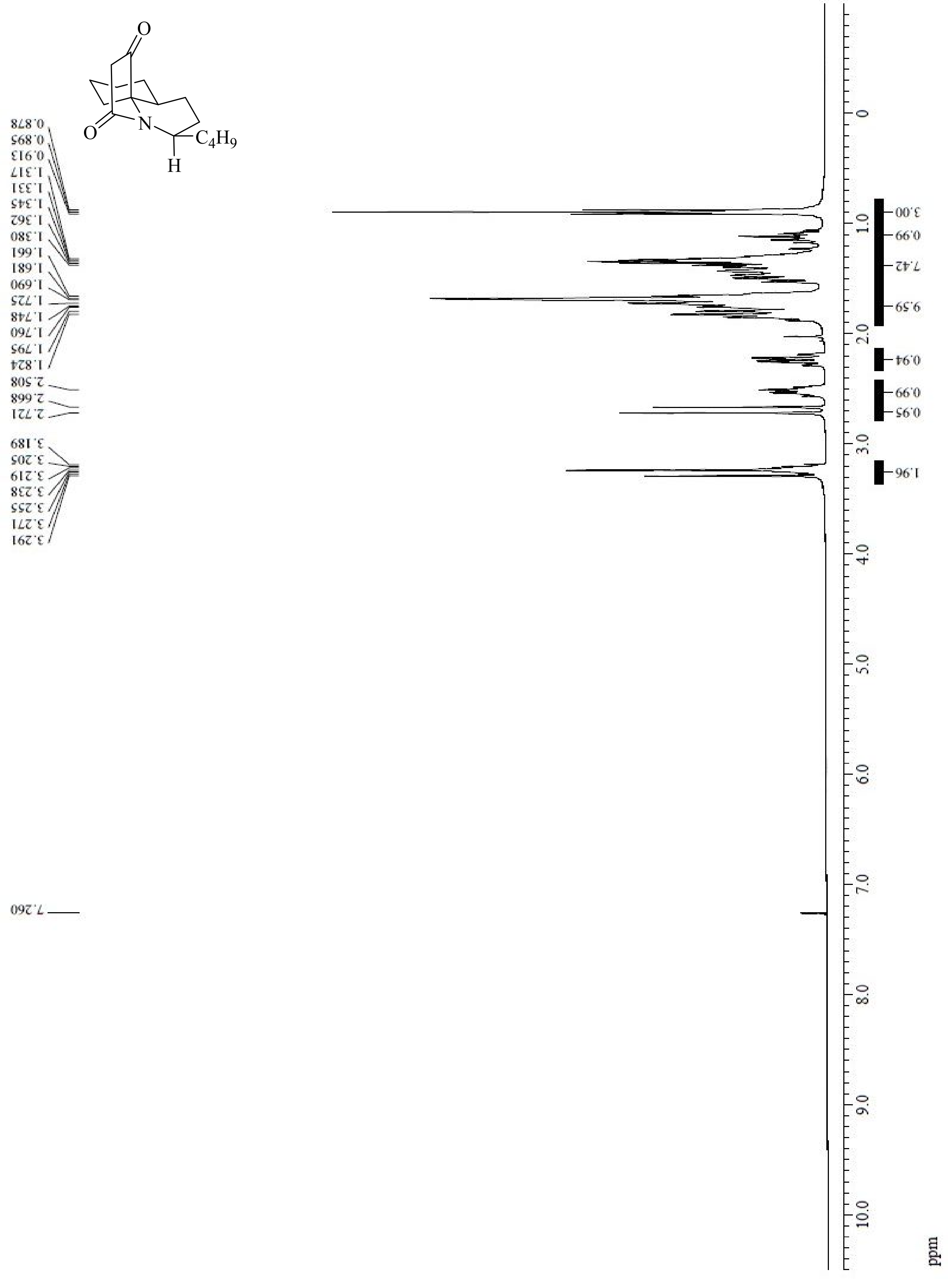

16, $^{13}C\{^{1}H\}$ NMR (101MHz, $CDCl_3$) 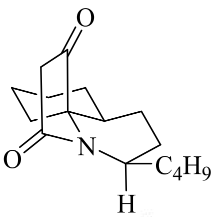

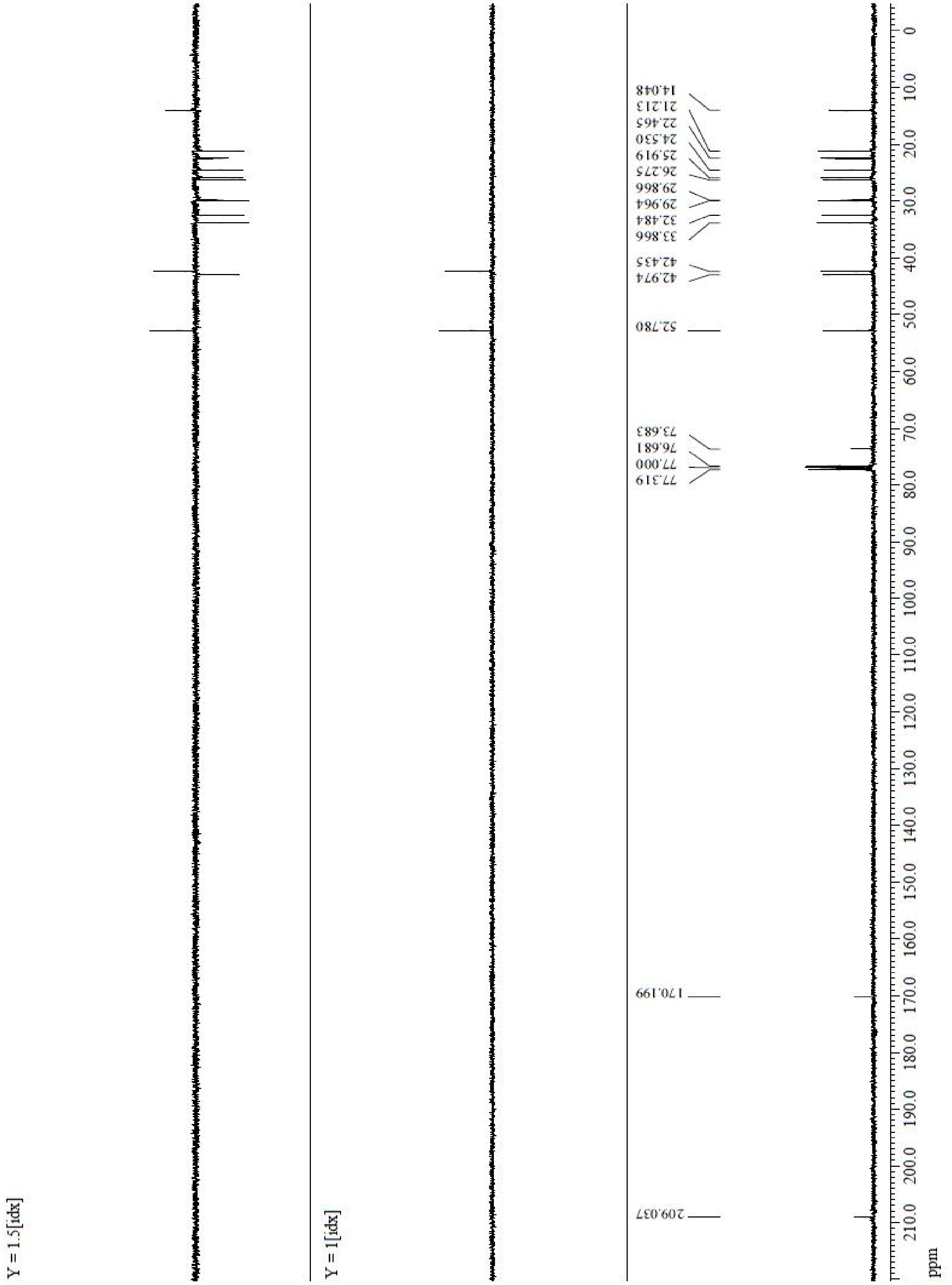

17, ^{1}H-NMR (400MHz, $CDCl_3$)

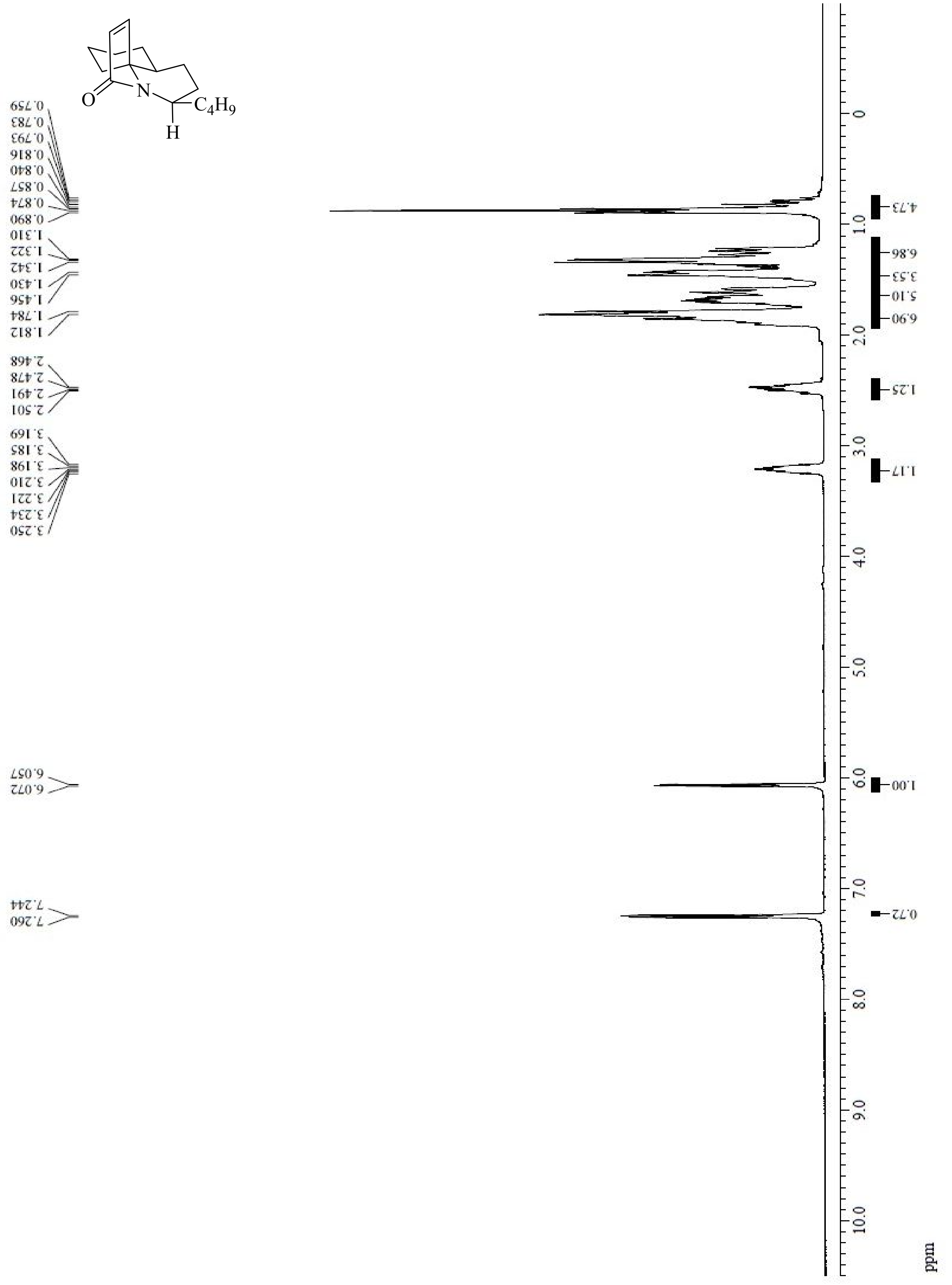

17, $^{13}C\{^{1}H\}$ NMR (101MHz, $CDCl_3$)

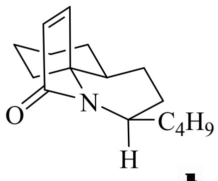

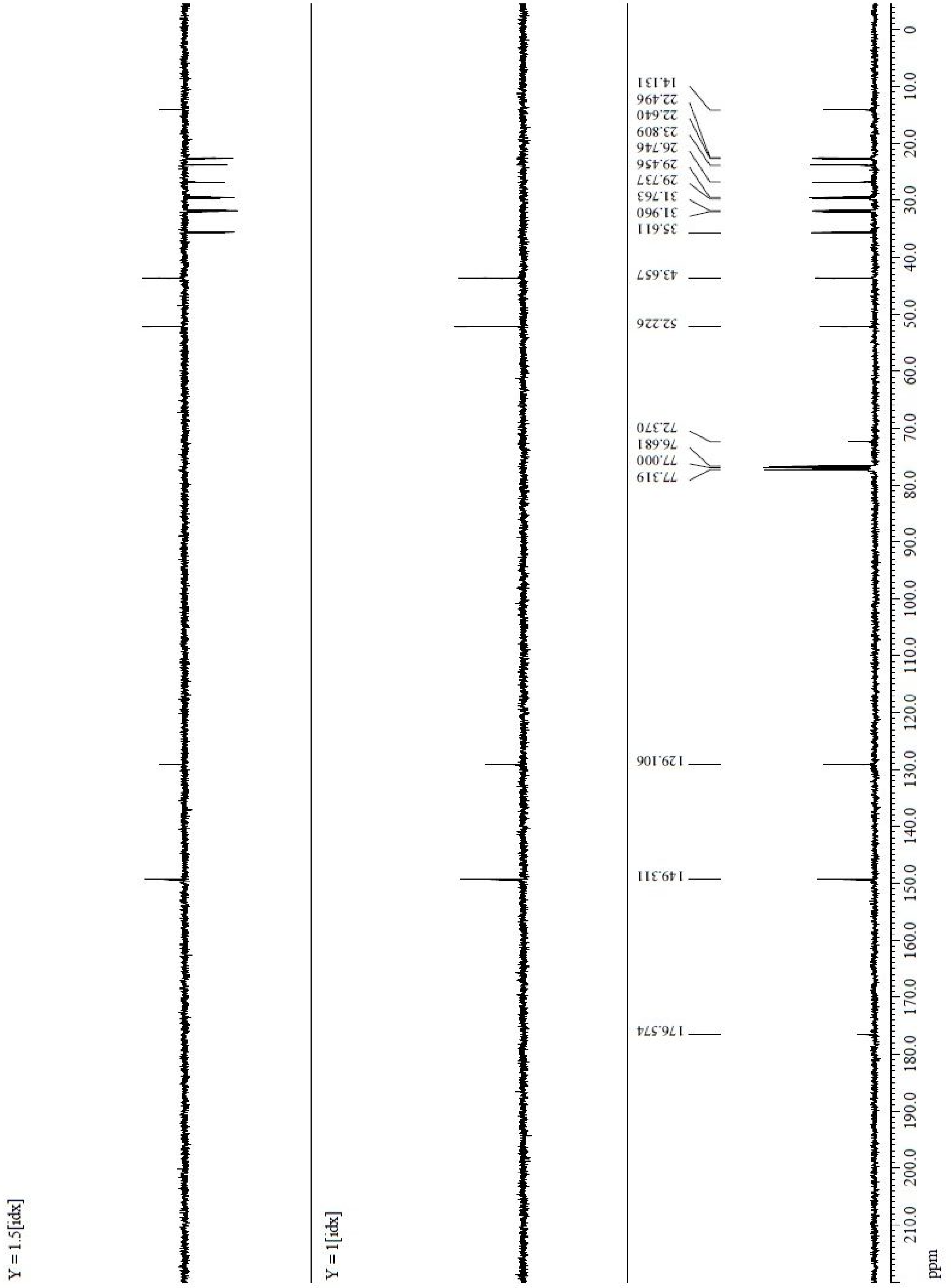

19, ^{1}H-NMR (400MHz, $CDCl_3$)

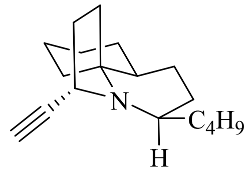

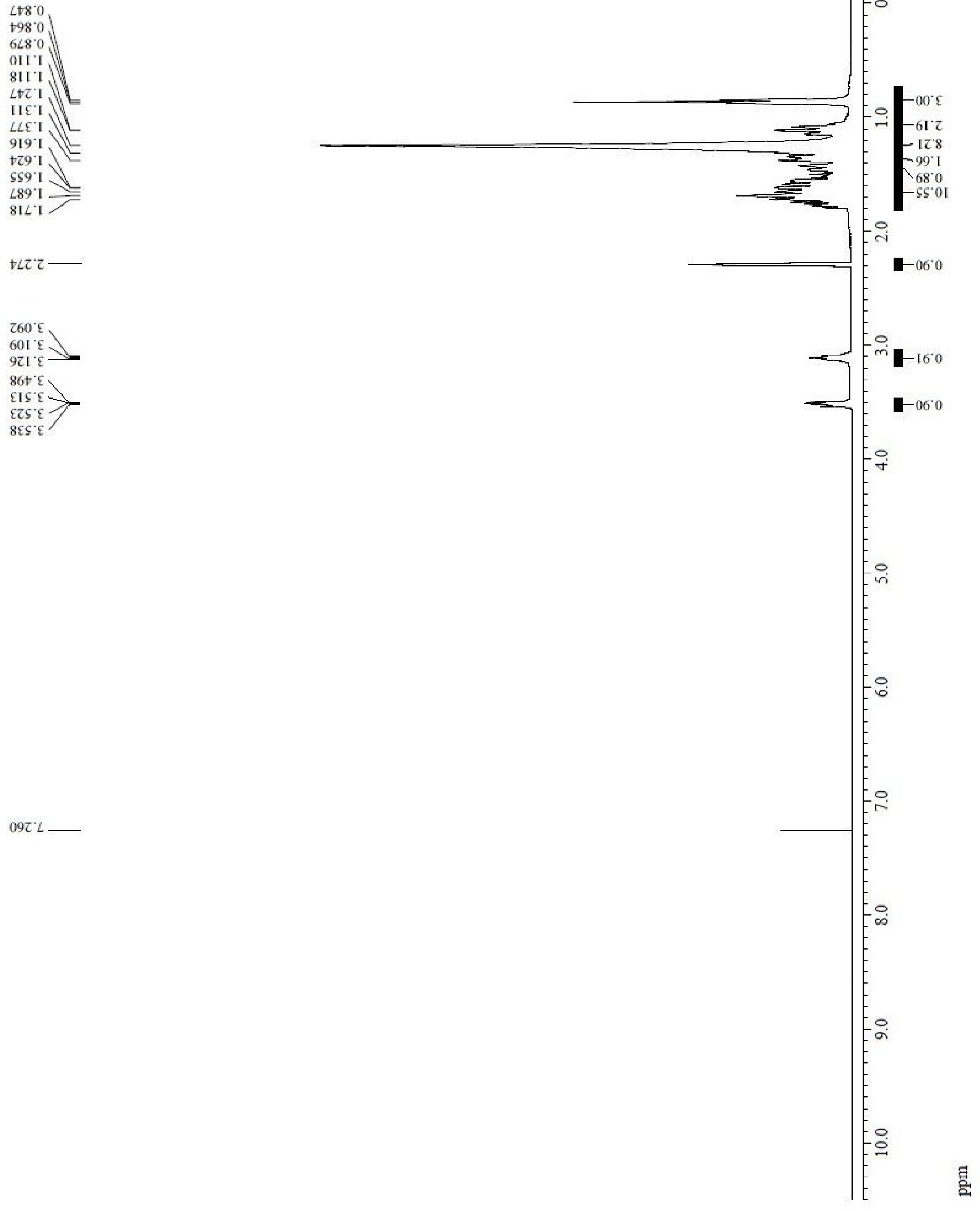

19, $^{13}C\{^{1}H\}$ NMR (101MHz, $CDCl_3$)

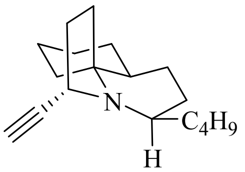

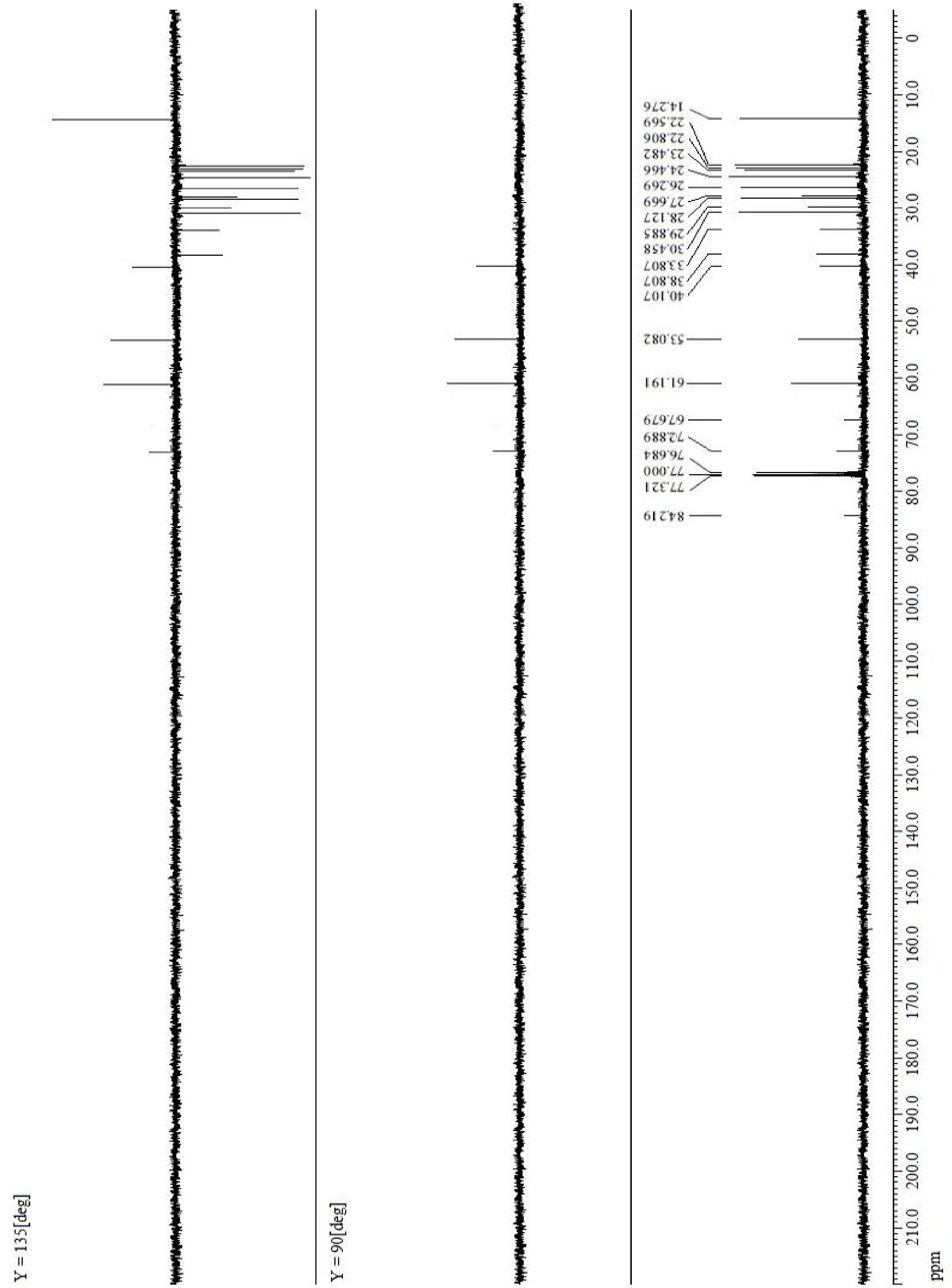

20, ^{1}H-NMR (400MHz, $CDCl_3$)

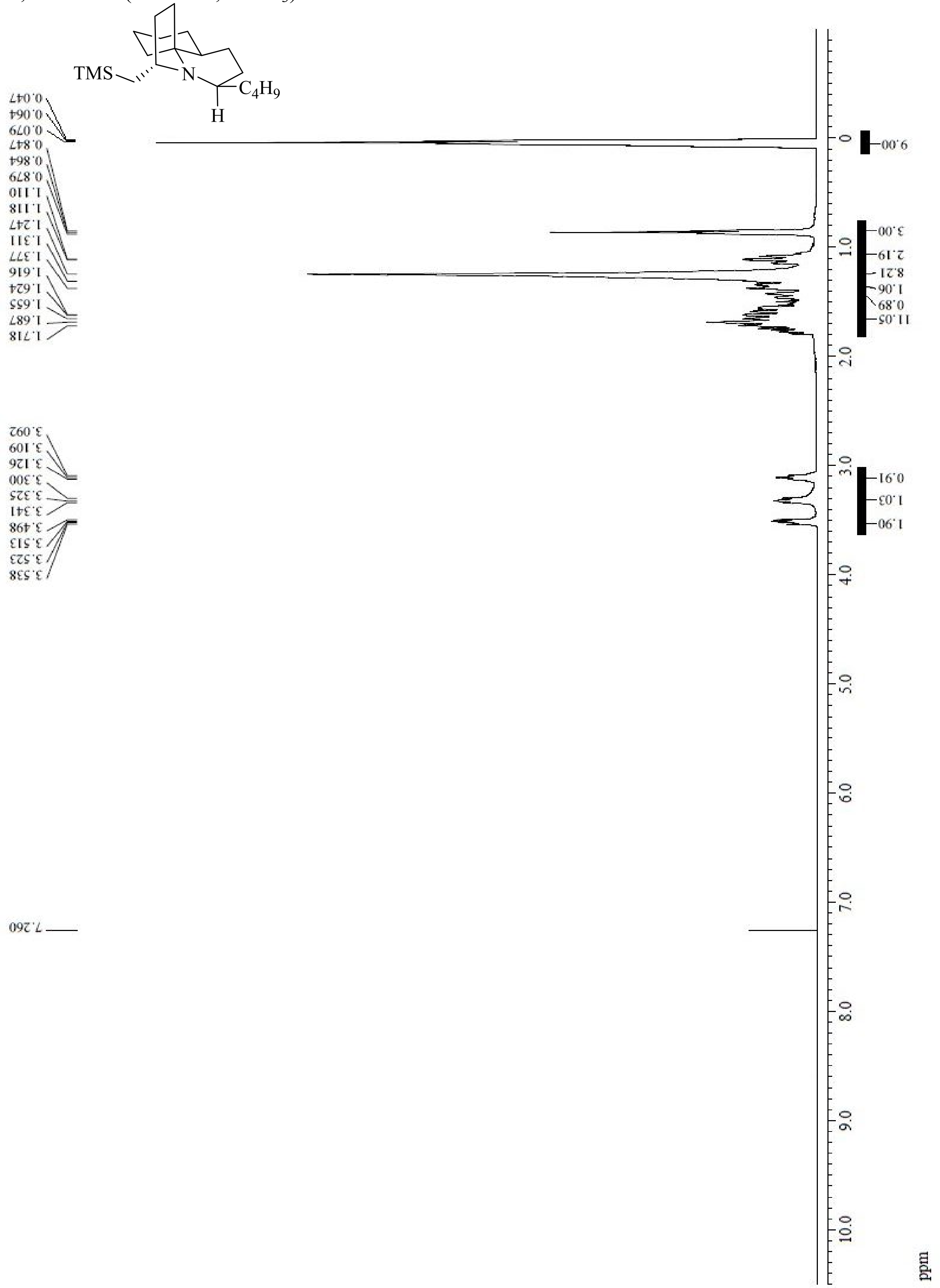

20, $^{13}C\{^{1}H\}$ NMR (101MHz, $CDCl_3$)

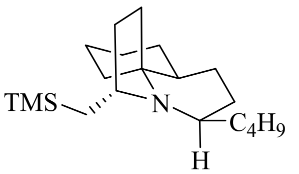

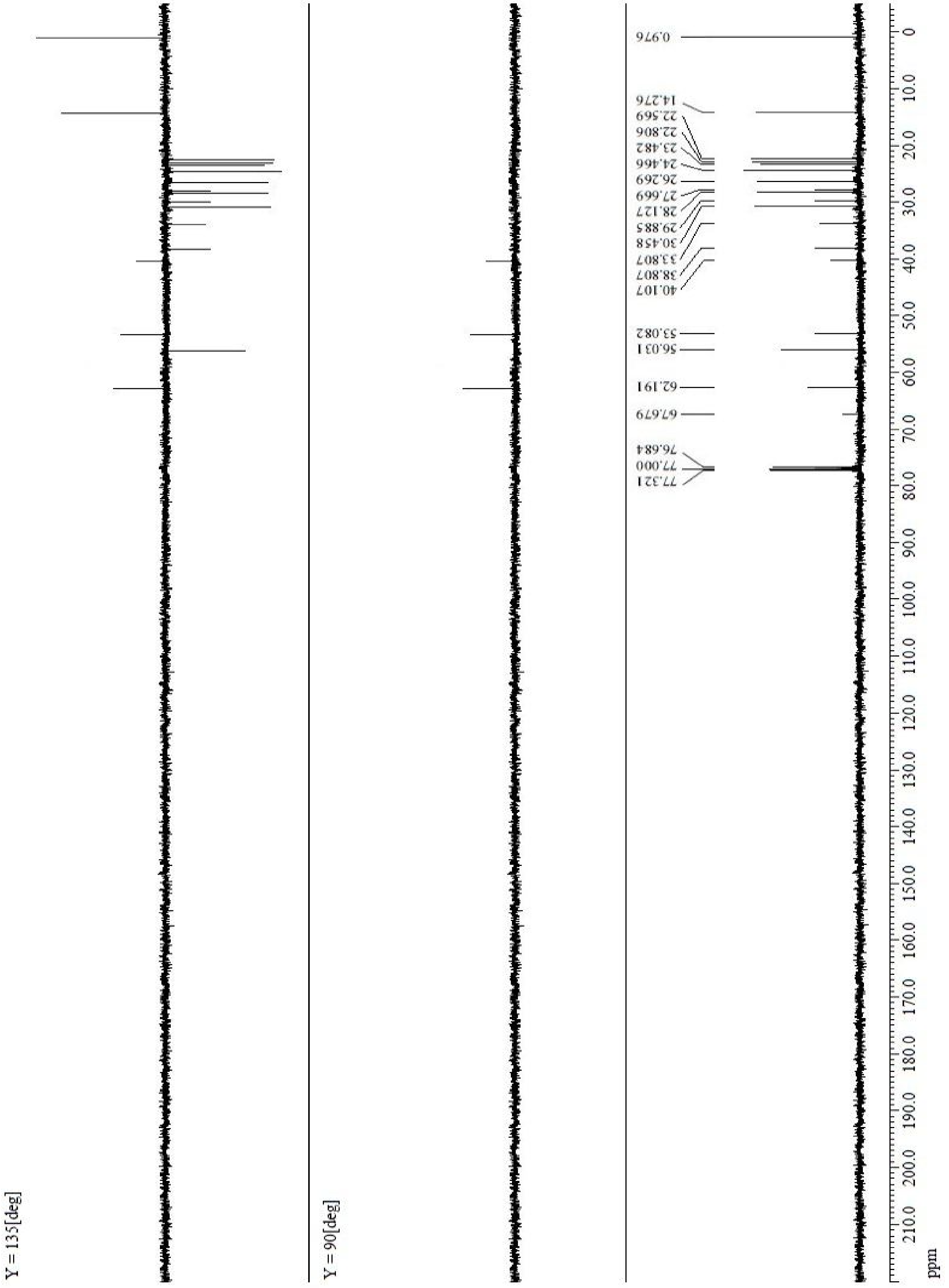

21, ^{1}H-NMR (400MHz, $CDCl_3$)

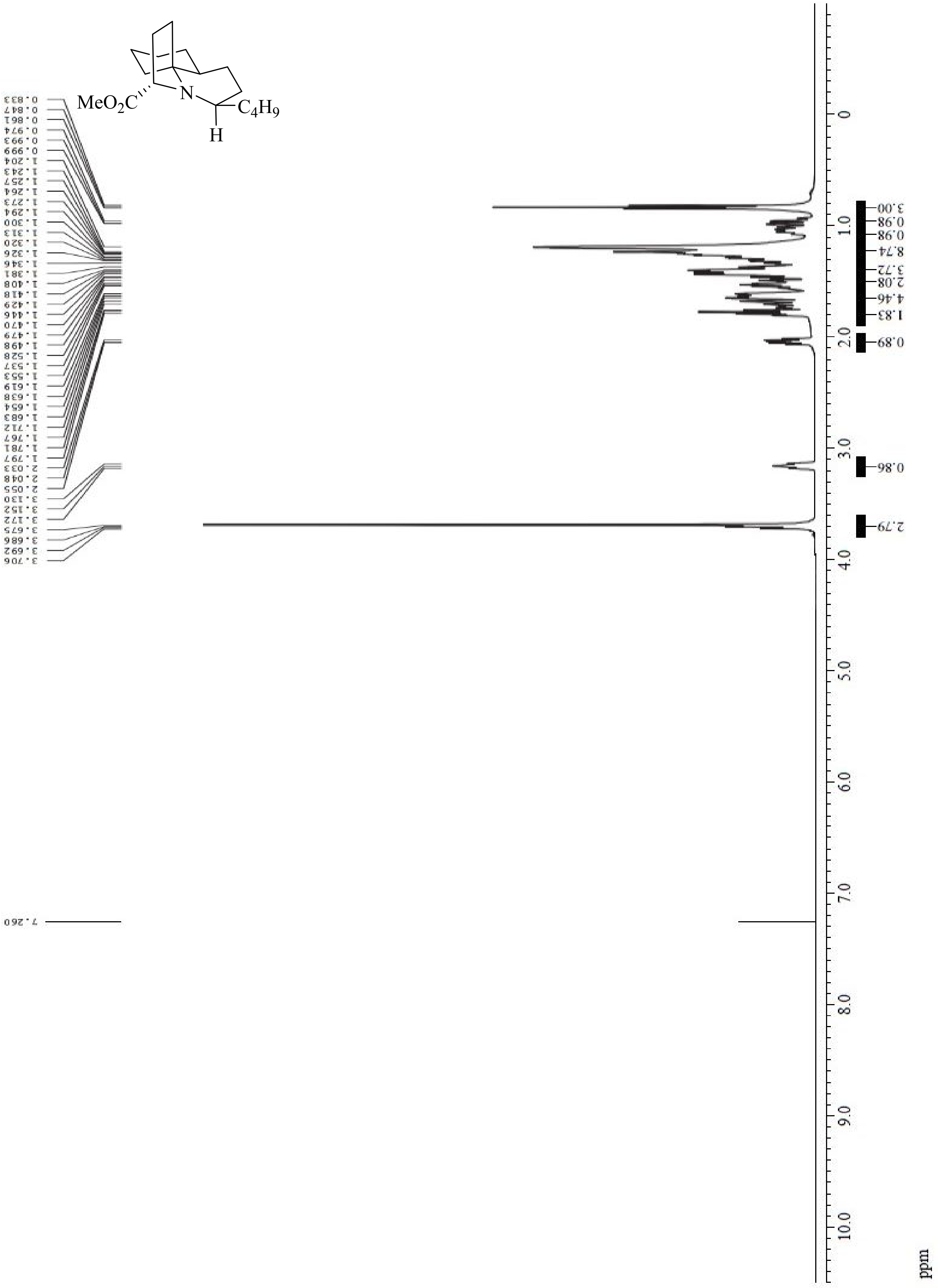

21, $^{13}C\{^{1}H\}$ NMR (101MHz, $CDCl_3$)

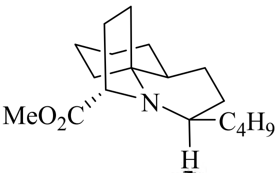

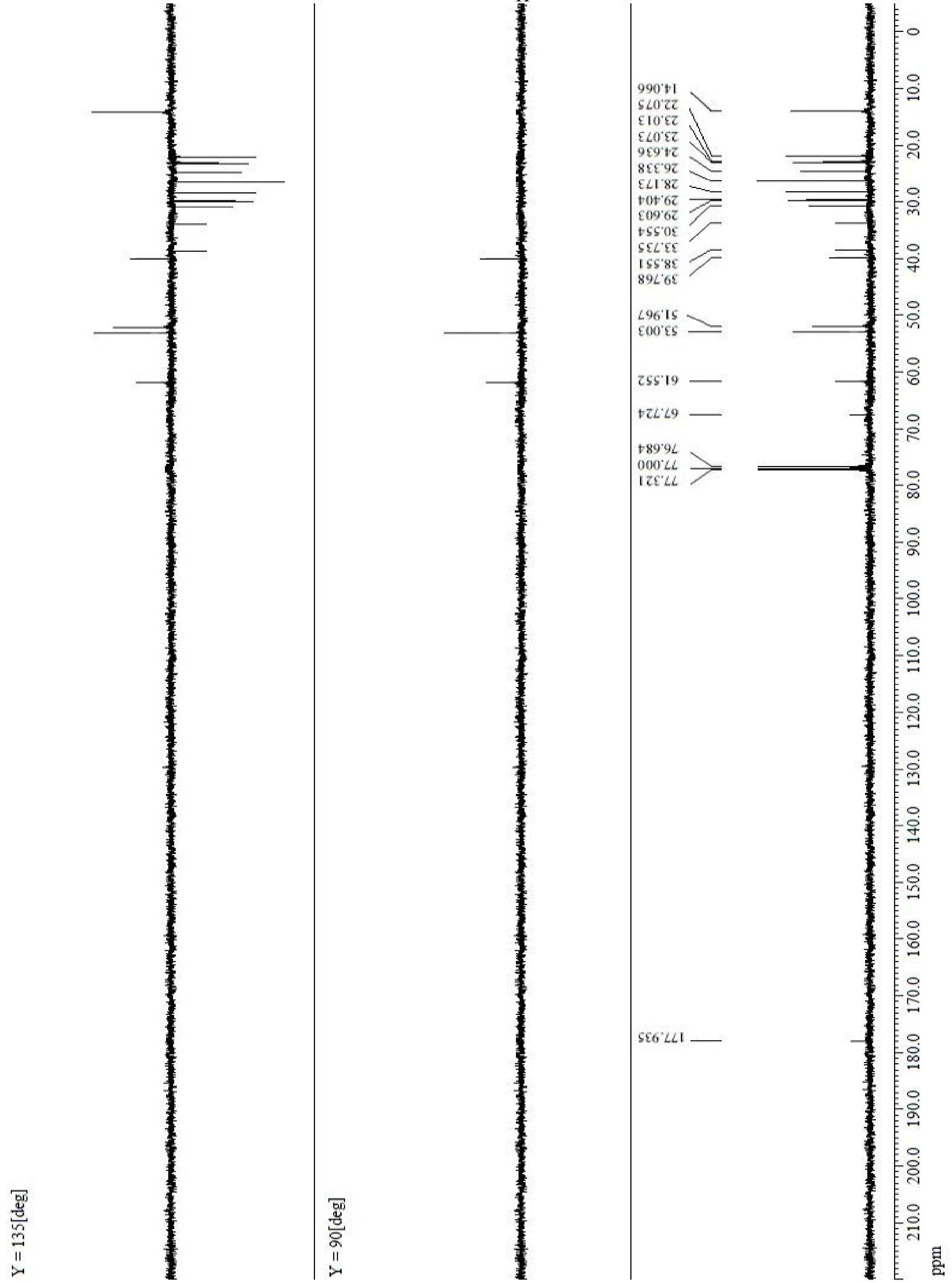

S-1, ^{1}H-NMR (400MHz, $CDCl_3$)

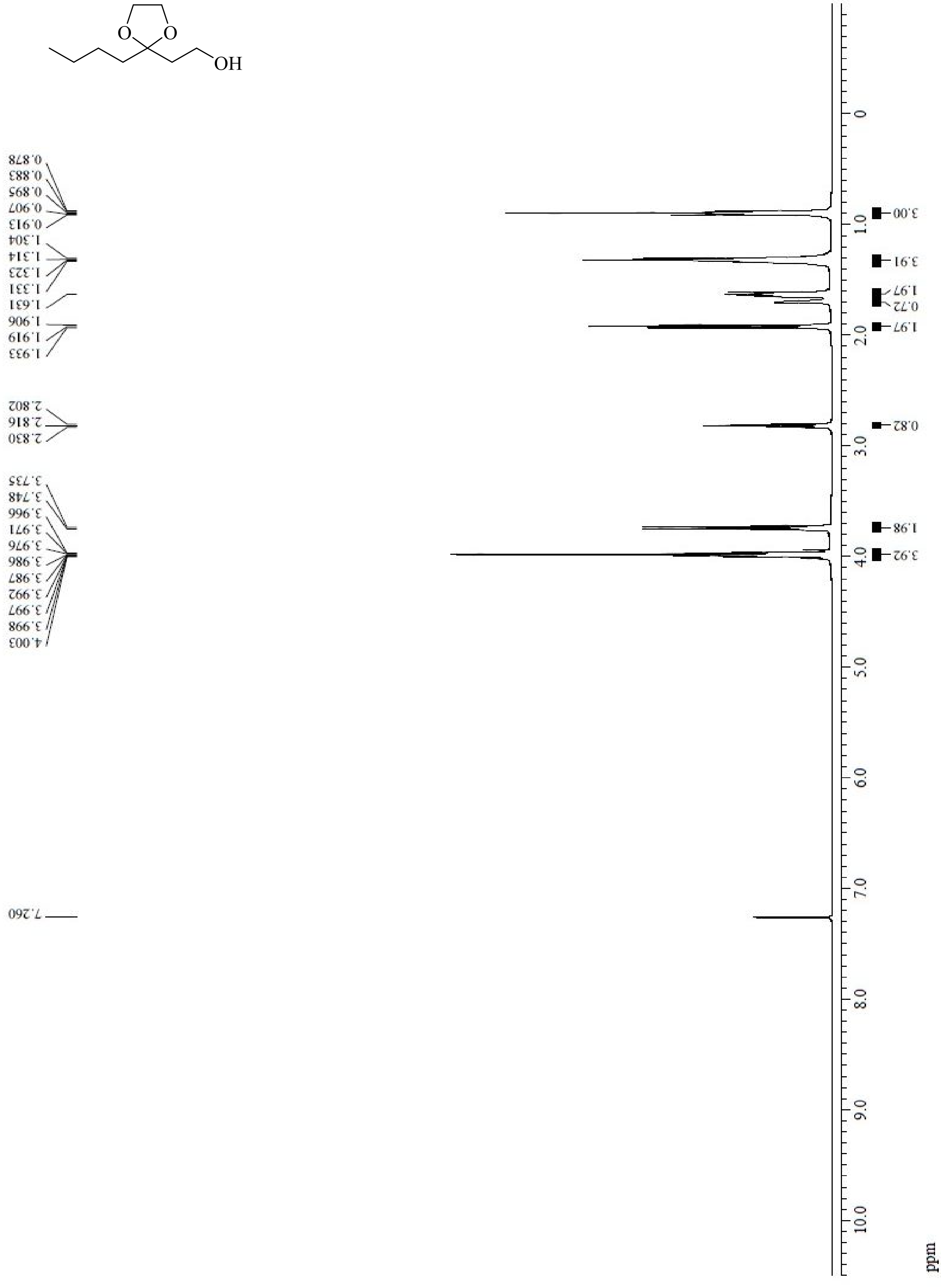

S-1, $^{13}C\{^{1}H\}$ NMR (101MHz, $CDCl_3$)

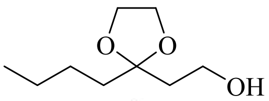

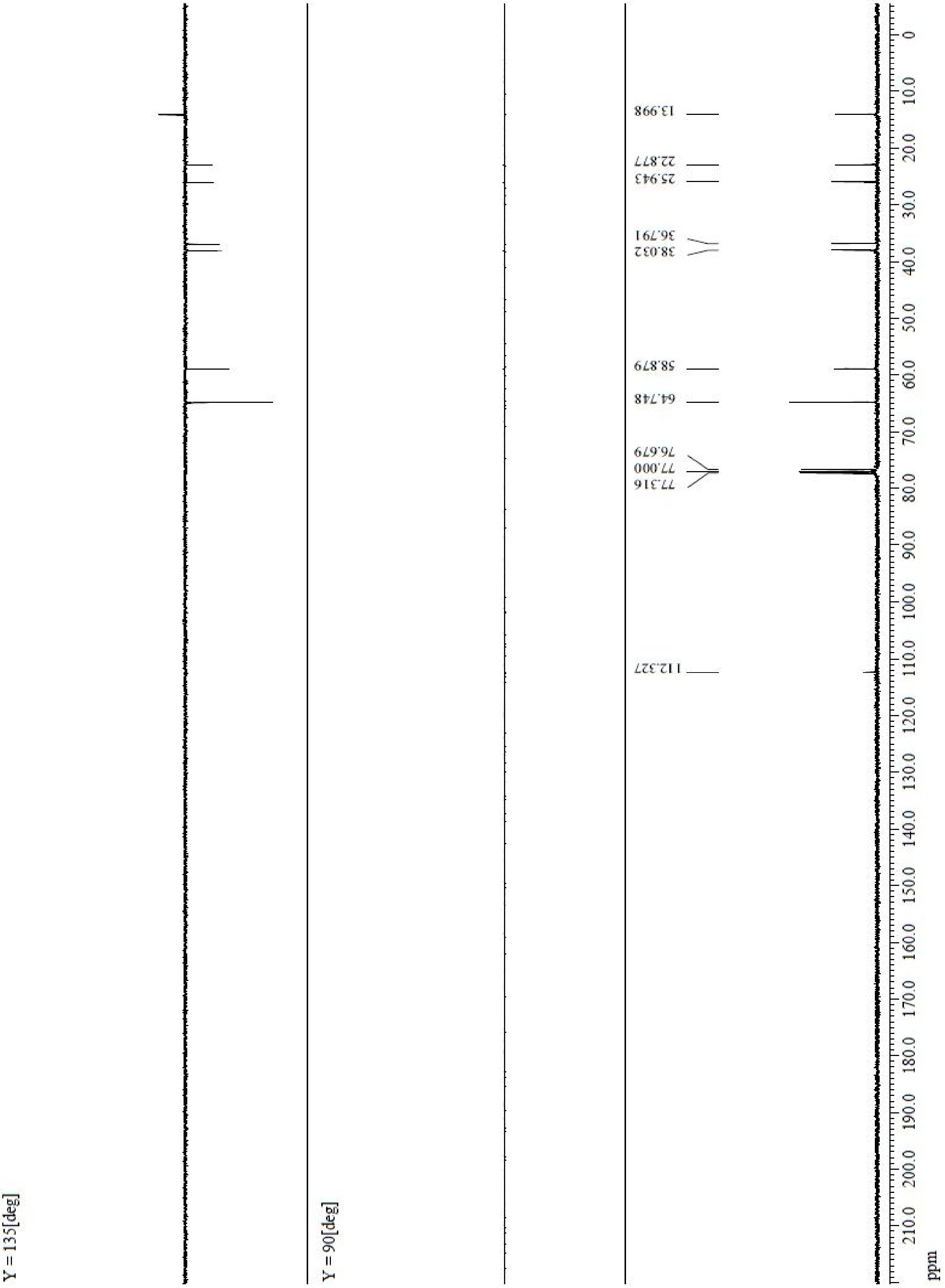

S-2, ^{1}H-NMR (400MHz, $CDCl_3$)

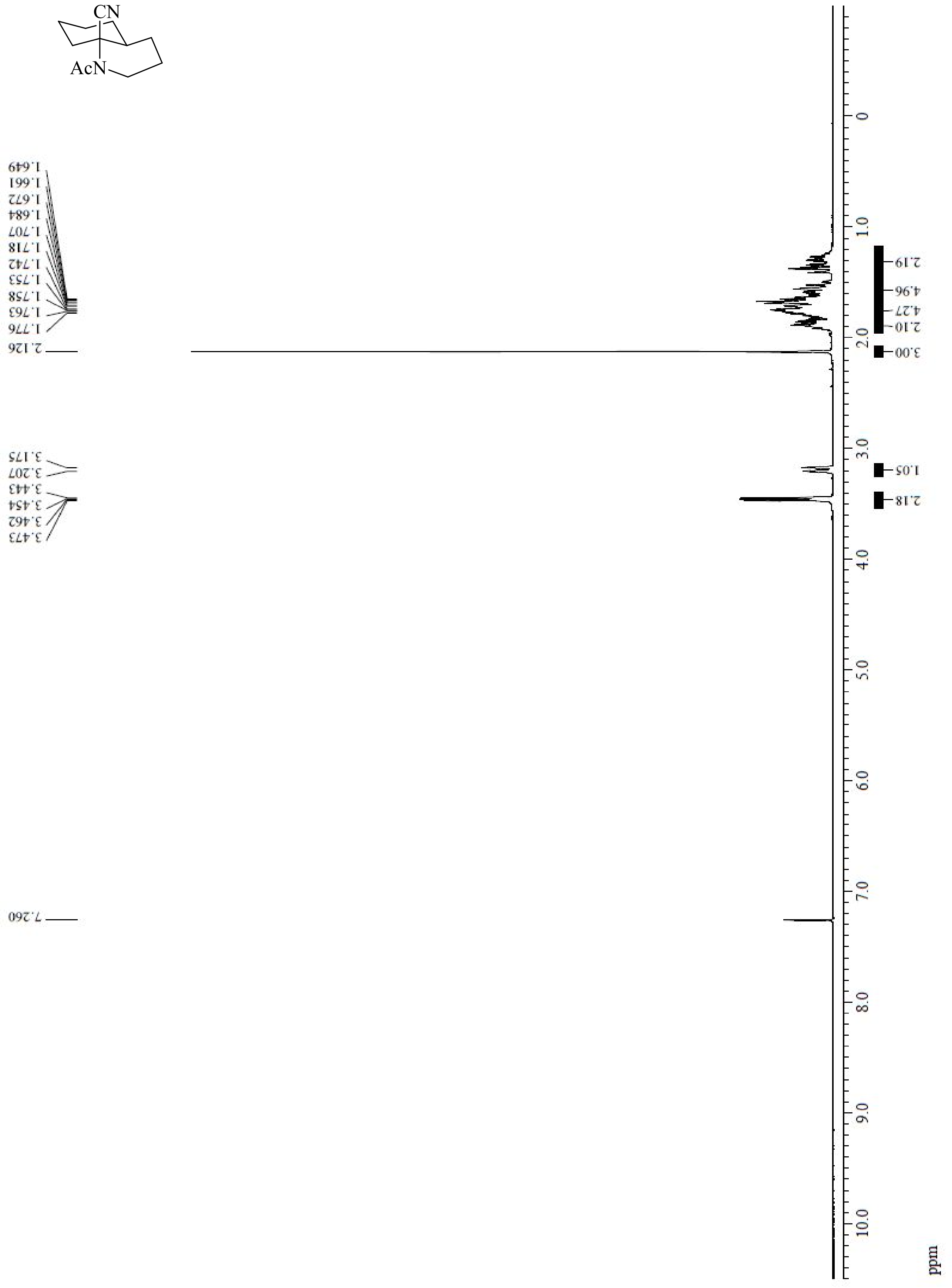

S-2, $^{13}C\{^{1}H\}$ NMR (101MHz, $CDCl_3$)

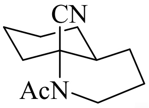

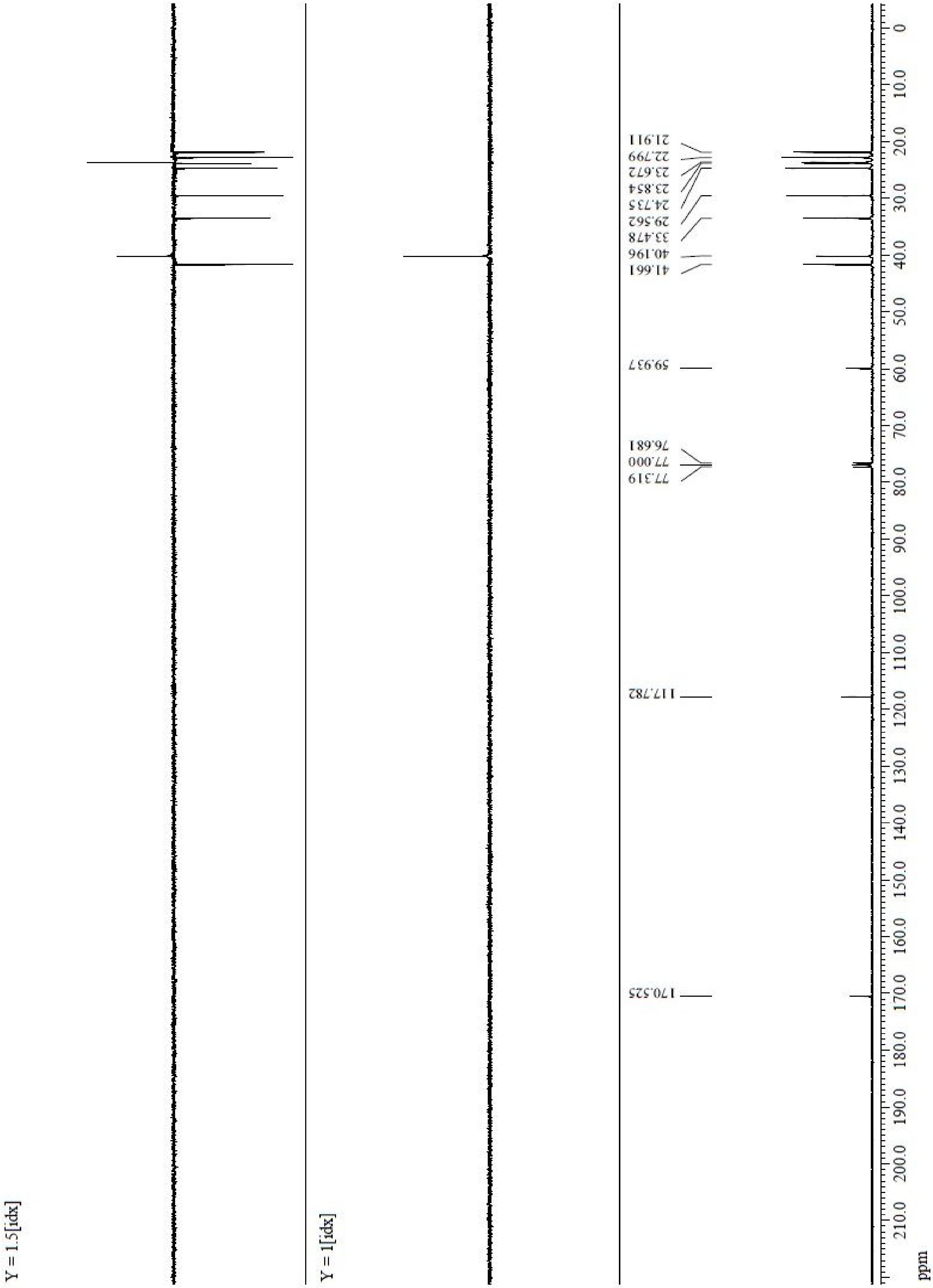

References:

(1) Iio, K.; Ramesh, N. G.; Okajima, A.; Higuchi, K.; Fujioka, H.; Akai, S.; Kita, Y., An Efficient Synthesis of peri-Hydroxy Aromatic Compounds via a Strong-Base-Induced [4+2] Cycloaddition of Homophthalic Anhydrides with Enolizable Enones. *J. Org. Chem.* **2000**, *65*, 89.

(2) The ratio was determined by 1H-NMR of the crude product based on the peak of δ 5.55 in **10** and that of δ 6.74 in **4**.

(3) Wang, Y.-T.; Wu, J.-L.; Chiou, W.-H., Total Synthesis of (±)-Fasicularin through Double Consecutive Epimerizations. *Org. Lett.* **2022**, *24*, 5957.

(4) Moorthy, J. N.; Singhal, N., Facile and Highly Selective Conversion of Nitriles to Amides via Indirect Acid-Catalyzed Hydration Using TFA or AcOH-H_2SO_4. *J. Org. Chem.* **2005**, *70*, 1926.

(5) For the role of HFIP in organic synthesis, see Motiwala, H. F.; Armaly, A. M.; Cacioppo, J. G.; Coombs, T. C.; Koehn, K. R. K.; Norwood, V. M. I. V.; Aubé, J. HFIP in Organic Synthesis. *Chem. Rev.* **2022**, *122*, 12544.

(6) Davis, M. C.; Stasko, D.; Chapman, R. D., Conversion of a Ketone to a Geminal Bisacetamide: Synthesis of 1,1-Bisacetamidocyclohexane. *Synth. Commun.* **2003**, *33*, 2677.

(7) Varma, R. S.; Naicker, K. P., The Urea−Hydrogen Peroxide Complex: Solid-State Oxidative Protocols for Hydroxylated Aldehydes and Ketones (Dakin Reaction), Nitriles, Sulfides, and Nitrogen Heterocycles. *Org. Lett.* **1999**, *1*, 189.

(8) Marce, P.; Lynch, J.; Blacker, A. J.; Williams, J. M. J., A mild hydration of nitriles catalysed by copper(II) acetate. *Chem. Commun.* **2016**, *52*, 1436.

(9) Lee, J.; Kim, M.; Chang, S.; Lee, H.-Y., Anhydrous Hydration of Nitriles to Amides using Aldoximes as the Water Source. *Org. Lett.* **2009**, *11*, 5598.

(10) Maffioli, S. I.; Marzorati, E.; Marazzi, A., Mild and Reversible Dehydration of Primary Amides with $PdCl_2$ in Aqueous Acetonitrile. *Org. Lett.* **2005**, *7*, 5237.

(11) Takayama, S.; Martin, R.; Wu, J.; Laslo, K.; Siuzdak, G.; Wong, C.-H., Chemoenzymatic Preparation of Novel Cyclic Imine Sugars and Rapid Biological Activity Evaluation Using Electrospray Mass Spectrometry and Kinetic Analysis. *J. Am. Chem. Soc.* **1997**, *119*, 8146.

(12) Perry, M. A.; Morin, M. D.; Slafer, B. W.; Rychnovsky, S. D., Total Synthesis of Lepadiformine Alkaloids using N-Boc α-Amino Nitriles as Trianion Synthons. *J. Org. Chem.* **2012**, *77*, 3390.

(13) Nishikawa, K.; Yamauchi, K.; Kikuchi, S.; Ezaki, S.; Koyama, T.; Nokubo, H.; Matsumura, K.; Kodama, T.; Kumagai, M.; Morimoto, Y., Total Syntheses of Lepadiformine Marine Alkaloids with Enantiodivergency, Utilizing $Hg(OTf)_2$-Catalyzed Cycloisomerization Reaction and their Cytotoxic Activities. *Chem. Eur. J.* **2017**, *23*, 9535.

(14) Wu, J.-L.; Chiou, W.-H., Diastereocontrolled Formal Syntheses of (±)-Lepadiformines A, B, and C and the Divergent Synthesis of 2-*epi*-Lepadiformine C through Unexpected Double Consecutive Epimerizations. *J. Org. Chem.* **2020**, *85*, 9051−9063.
